# Supplementary material for: PAMAM-Calix-Dendrimers: Second Generation Synthesis, Fluorescent Properties and Catecholamines Binding
Source: Pharmaceutics. 2022 Dec 8;14(12):2748. doi: 10.3390/pharmaceutics14122748 (PMC9781744; doi:10.3390/pharmaceutics14122748)
Supplement: Supplementary file 1 [file pharmaceutics-14-02748-s001.zip › pharmaceutics-2035199-resub supplementary V2.pdf]

pharmaceutics

Electronic Supplementary Information

PAMAM-calix-dendrimers: second generation synthesis, fluorescent properties and catecholamines binding

Olga Mostovaya, Igor Shiabiev, Dmitry Pysin, Alesia Stanavaya, Viktar Abashkin, Dzmitry Shcharbin, Pavel Padnya, Ivan Stoikov

1. NMR, IR and mass spectra of the synthesized compounds 2

2. Spectral properties of the synthesized compounds 17

3. Complexation investigation 21

3.1. UV-Vis spectra 21

3.2. Fluorescence spectra 28

3.3. ^{1}H and 2D ^{1}H-^{1}H NOESY NMR spectra 38

3.4. DLS data 42

3.5. TEM spectroscopy 62

3.6. Release investigation 66

1. NMR, IR and mass spectra of the synthesized compounds

^{1}H NMR, ^{13}C NMR and ^{1}H–^{1}H NOESY NMR spectra were obtained on the Bruker Avance-400 spectrometer (Bruker Corp., Billerica, MA, USA) (^{13}C{^{1}H} 100 MHz and ^{1}H 400 MHz). Chemical shifts were determined against the signals of residual protons of deuterated solvent ($CDCl_3$, CD_3OD). Concentrations of the compounds were equal to 3–5 mass. % for the most records and 1×10^{-2} M for the ^{1}H–^{1}H NOESY experiments. FTIR ATR spectra were recorded on the Spectrum 400 FT-IR spectrometer (Perkin–Elmer, Seer Green, Llantrisant, UK) with the Diamond KRS-5 attenuated total internal reflectance attachment (resolution 0.5 cm^{-1}, accumulation of 64 scans, recording time 16 s in the wavelength range 400–4000 cm^{-1}). Elemental analysis was performed on the Perkin–Elmer 2400 Series II instrument (Perkin–Elmer, Waltham, MA, USA). Melting points were determined using the Boetius Block apparatus (VEB Kombinat Nagema, Radebeul, Germany). ESI HRMS experiments were performed at Agilent 6550 iFunnel Q-TOF LC/MS (Agilent Technologies, Santa Clara, CA, USA), equipped with Agilent 1290 Infinity II LC.

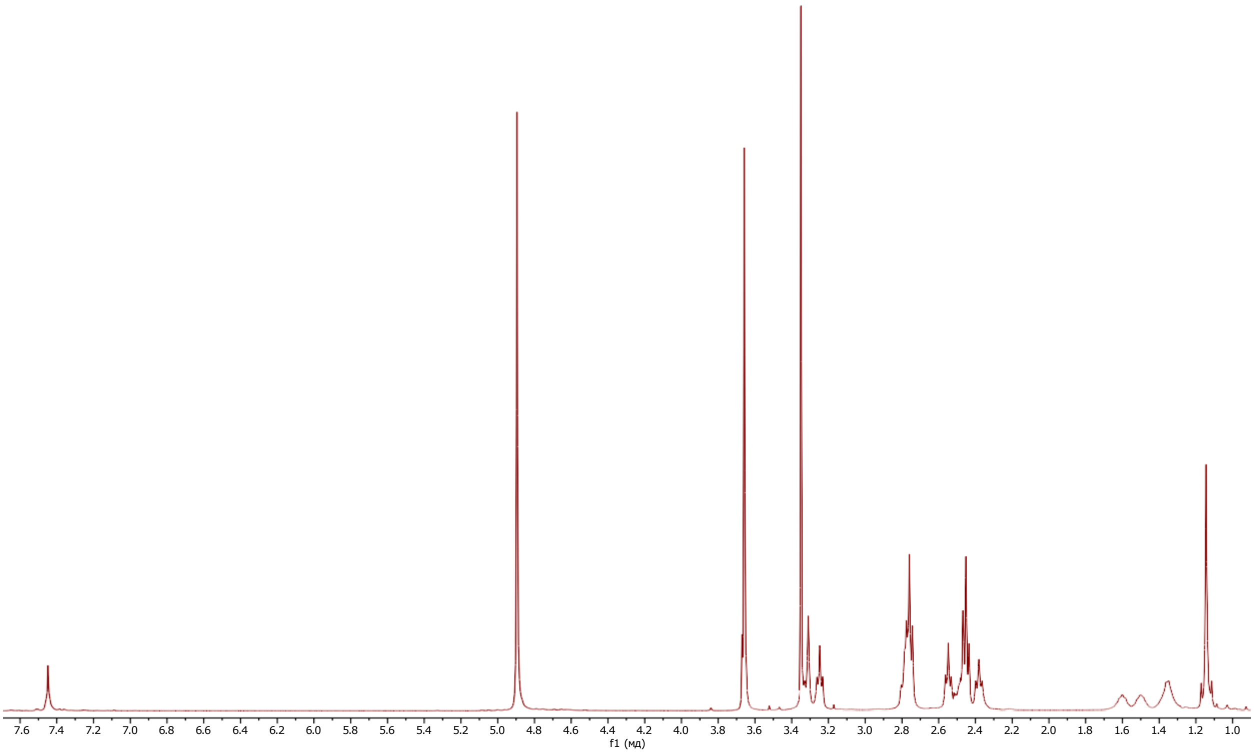

Figure S1. ^{1}H NMR spectrum of **G1.5-cone**, CD_3OD, 298 K, 400 MHz

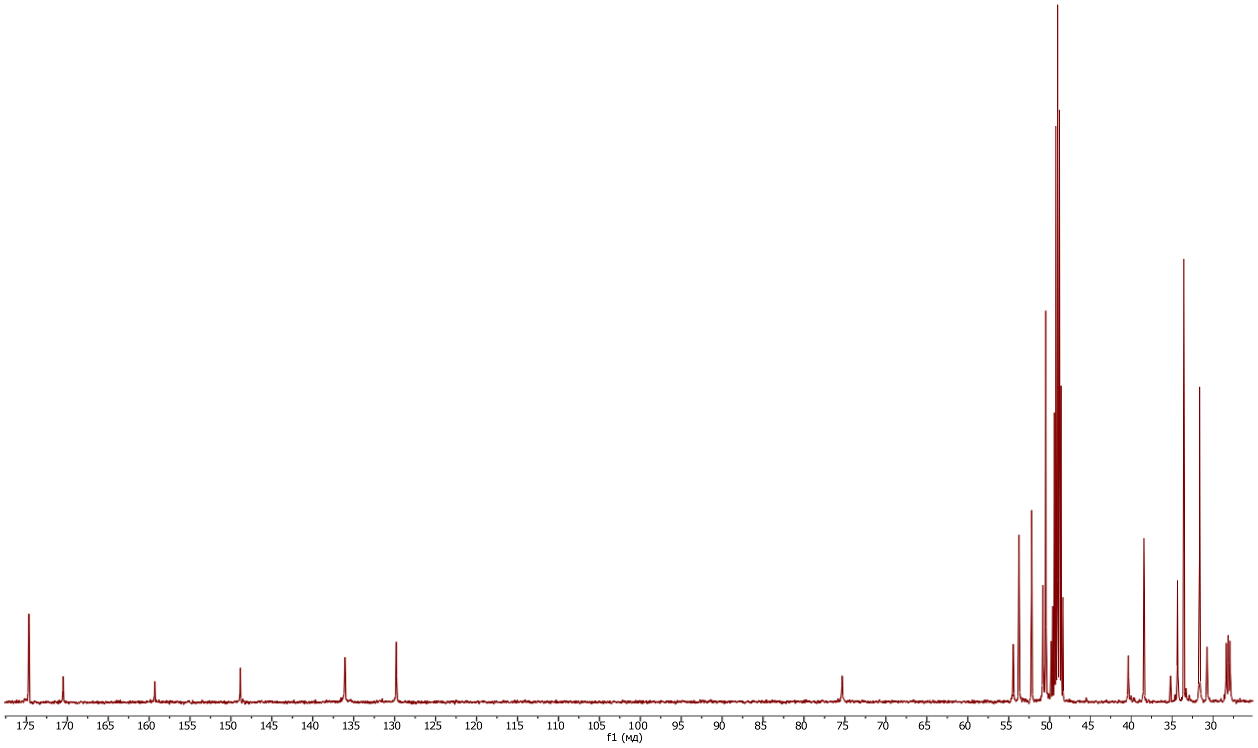

Figure S2. $^{13}C\{^{1}H\}$ NMR spectrum of **G1.5-cone**, CD_3OD, 298 K, 100 MHz

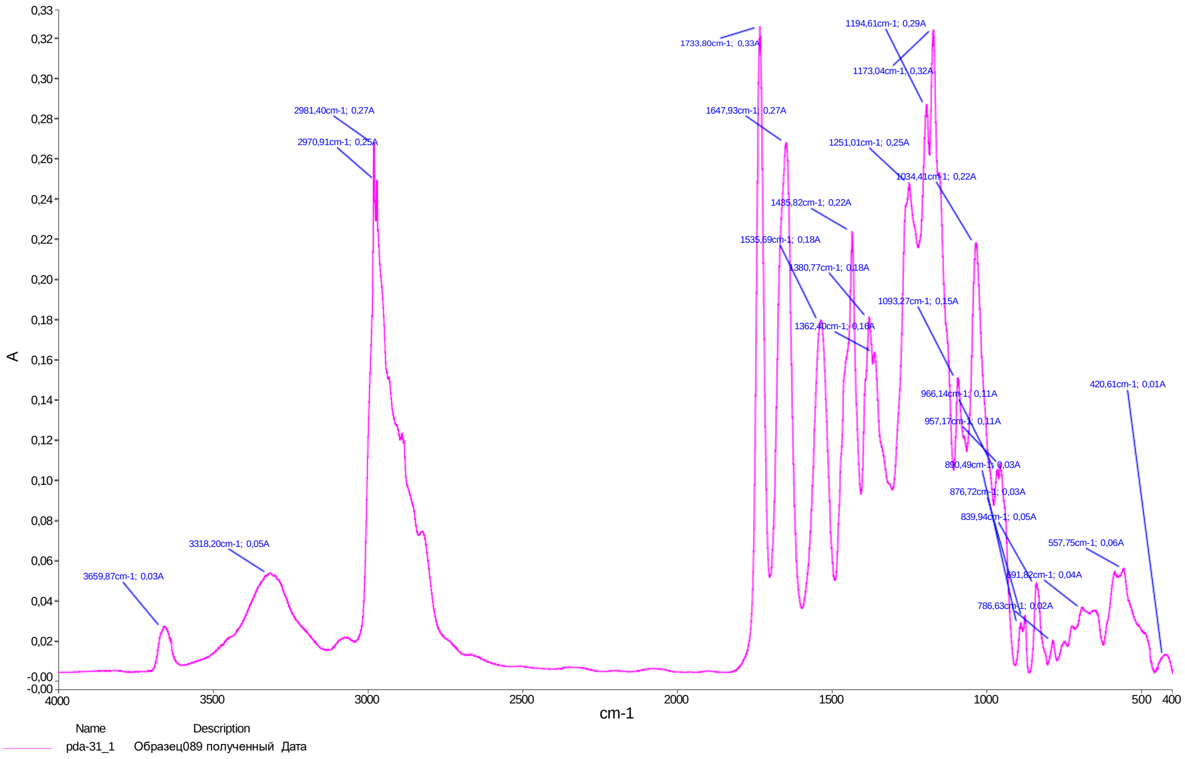

Figure S3. FTIR-ATR spectrum of **G1.5-cone**

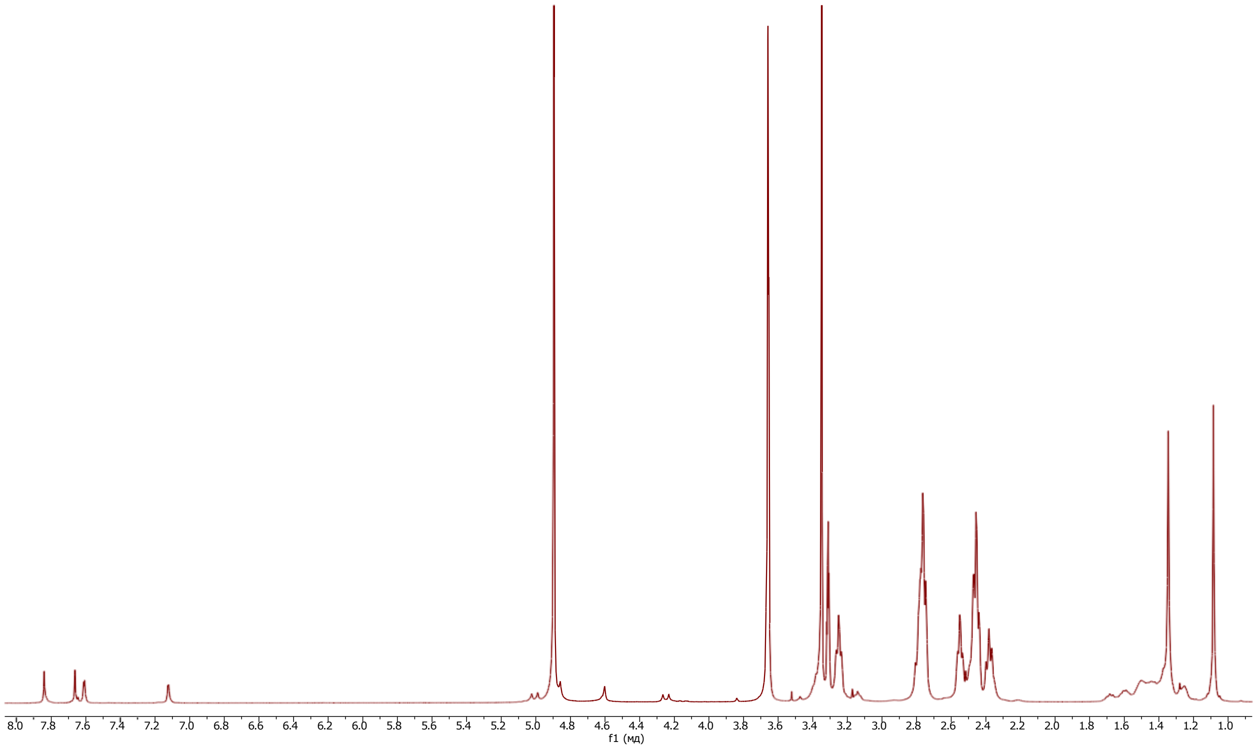

Figure S4. ^{1}H NMR spectrum of **G1.5-paco**, CD_3OD, 298 K, 400 MHz

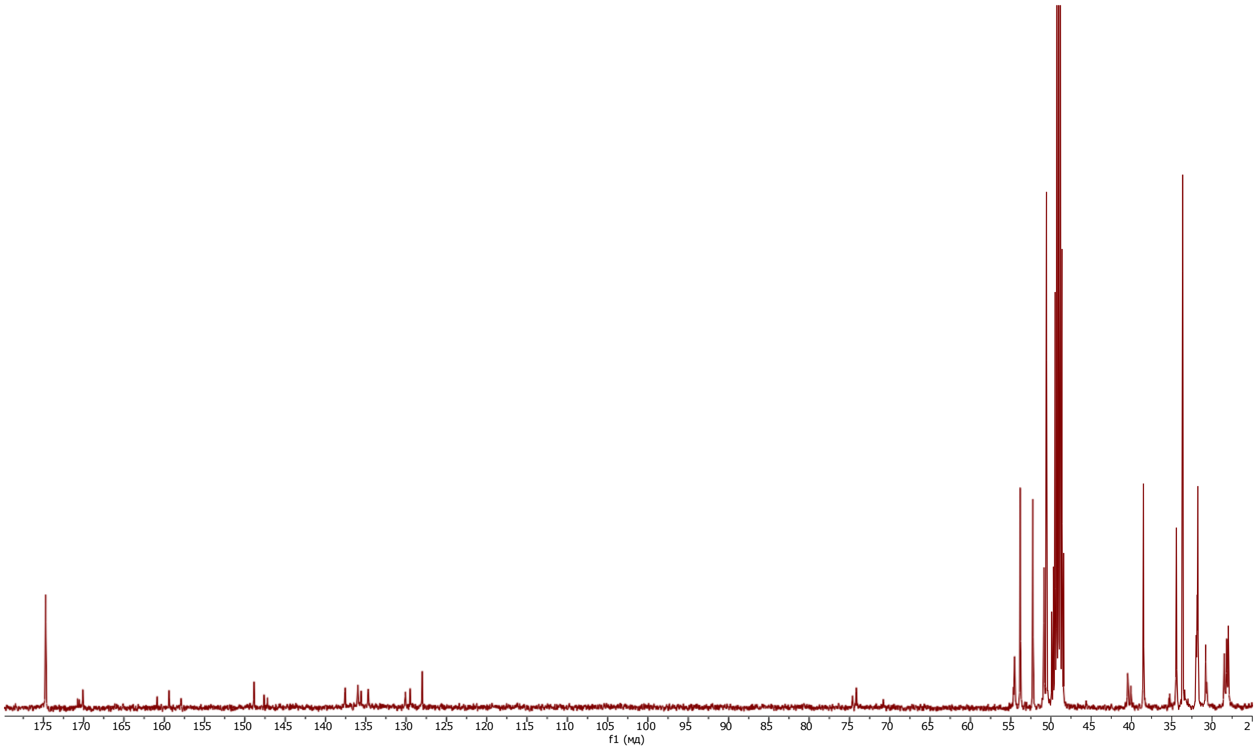

Figure S5. $^{13}C\{^{1}H\}$ NMR spectrum of **G1.5-paco**, CD_3OD, 298 K, 100 MHz

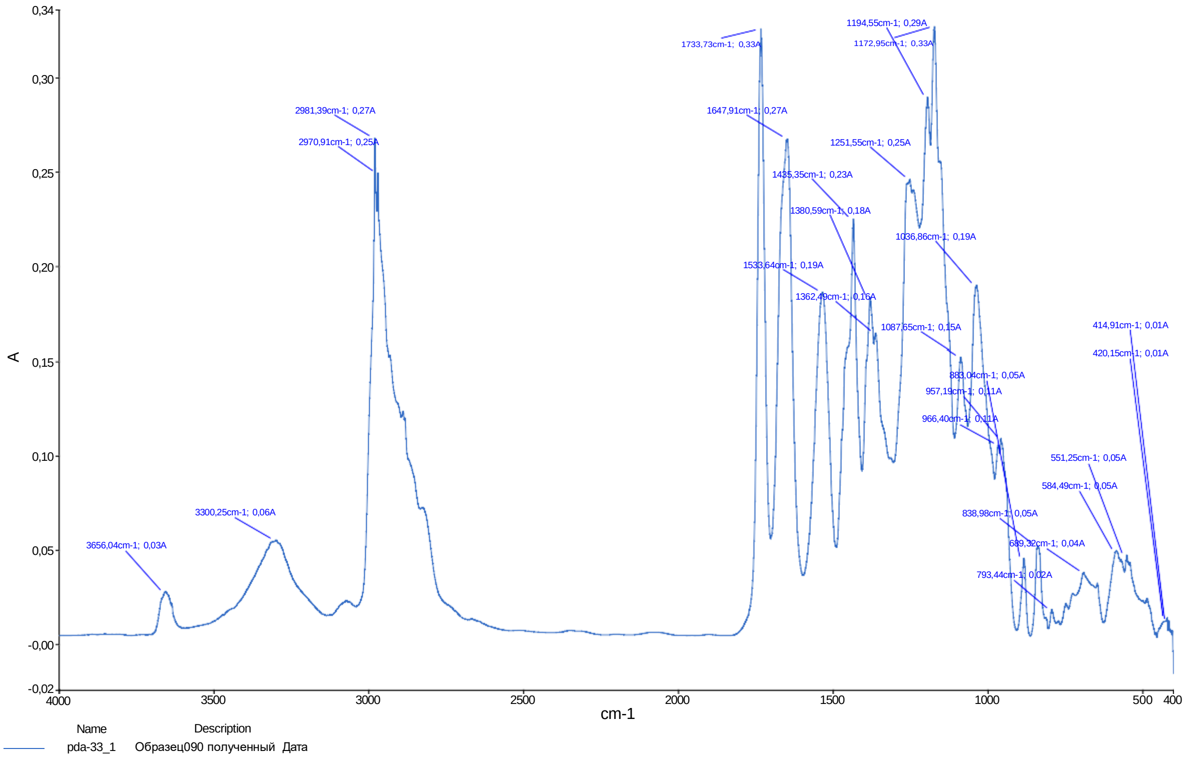

Figure S6. FTIR-ATR spectrum of **G1.5-paco**

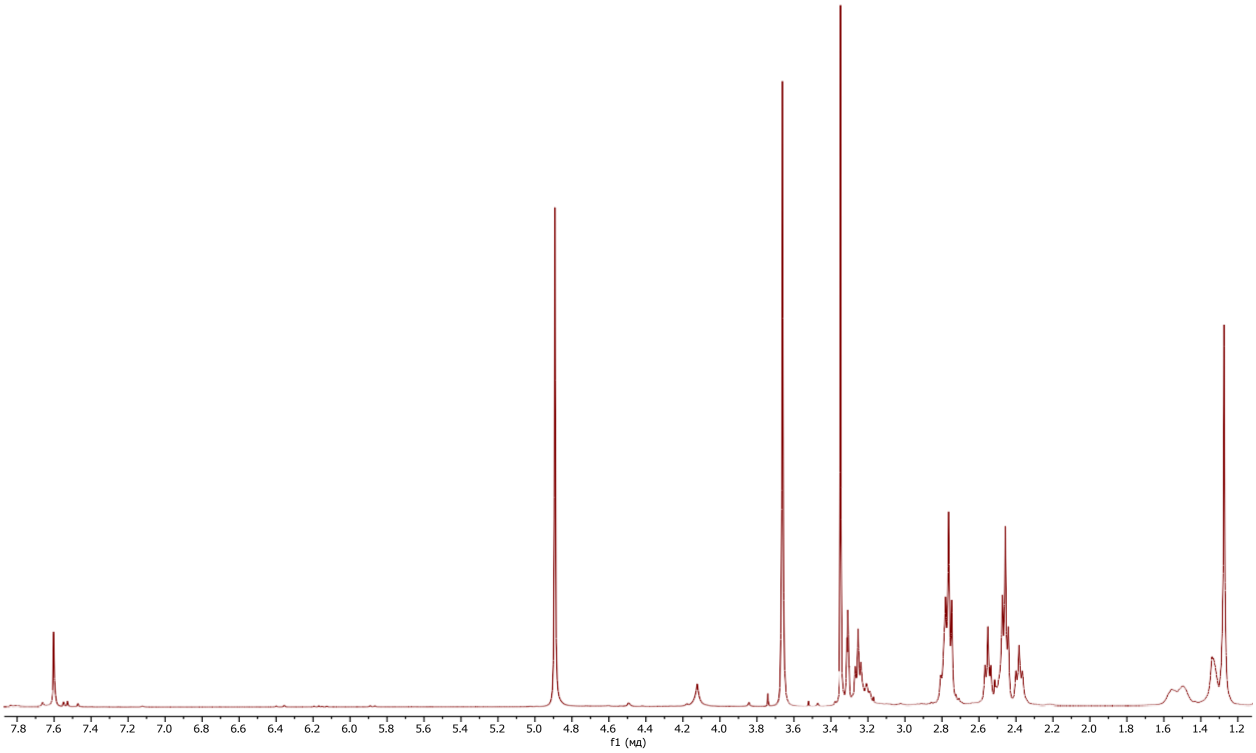

Figure S7. ^{1}H NMR spectrum of **G1.5-alt**, CD_3OD, 298 K, 400 MHz

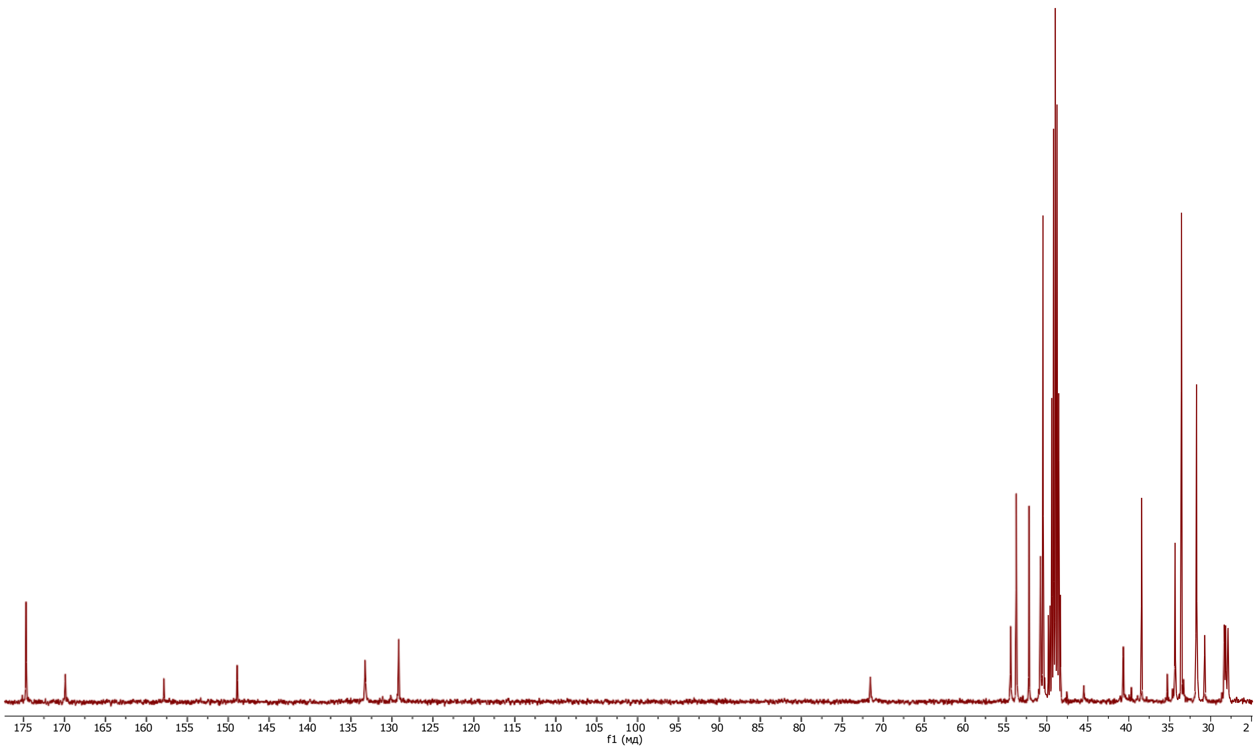

Figure S8. $^{13}C\{^{1}H\}$ NMR spectrum of **G1.5-alt**, CD_3OD, 298 K, 100 MHz

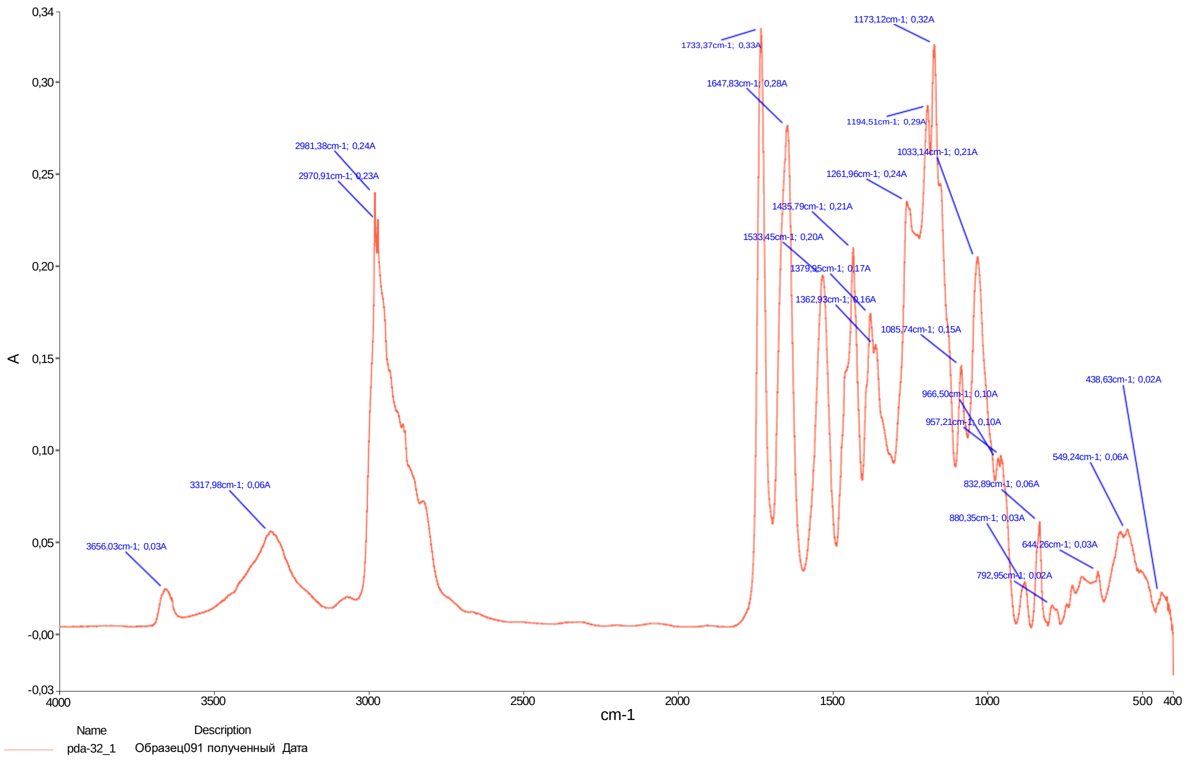

Figure S9. FTIR-ATR spectrum of **G1.5-alt**

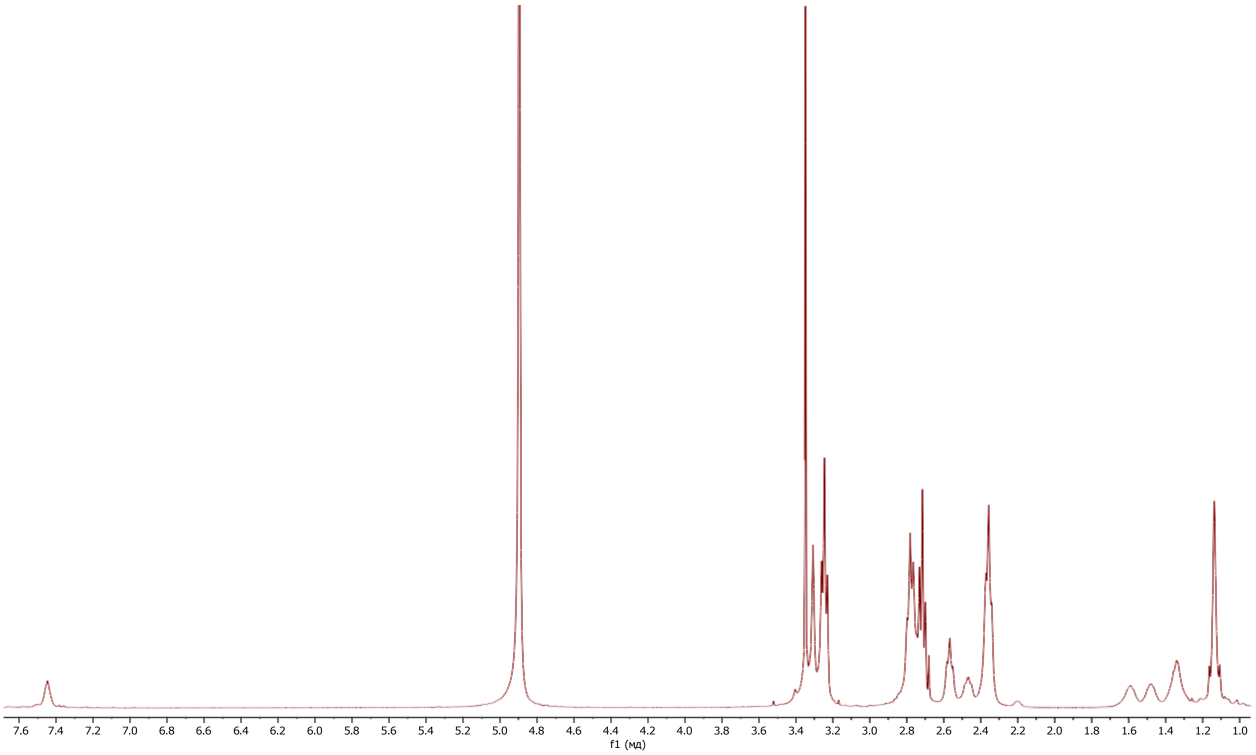

Figure S10. ^{1}H NMR spectrum of **G2-cone**, CD_3OD, 298 K, 400 MHz

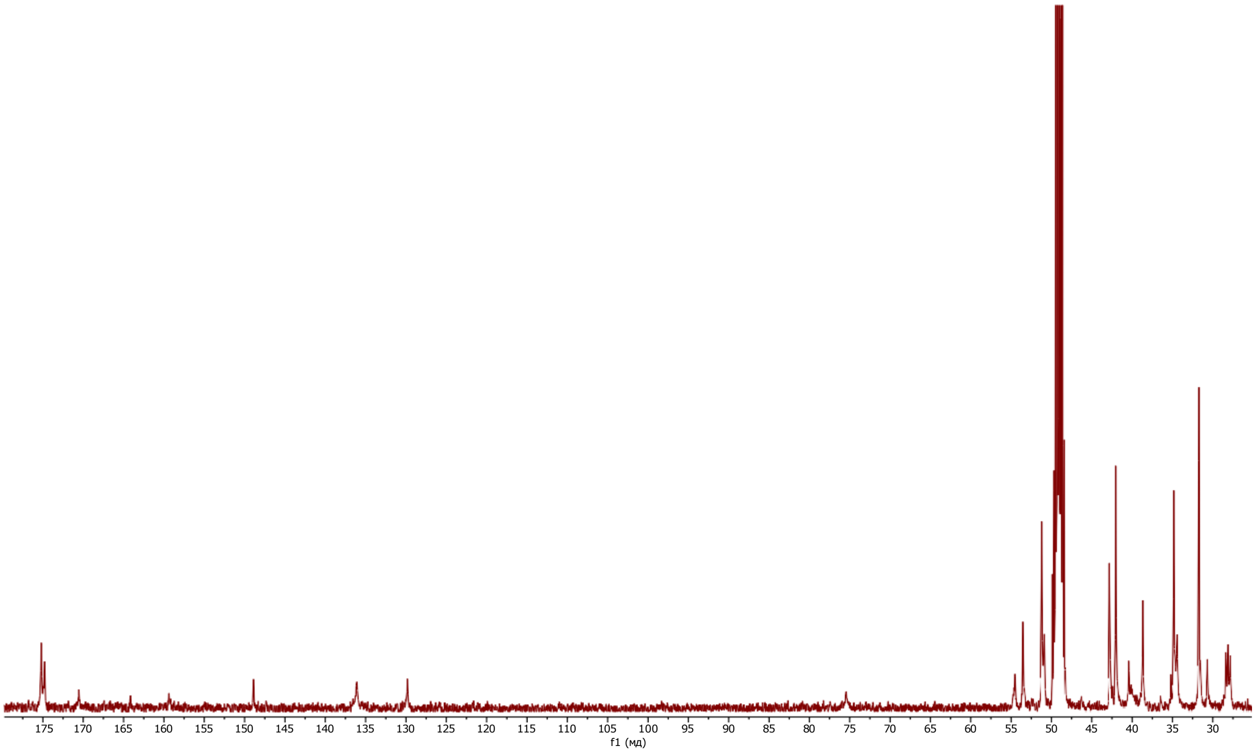

Figure S11. $^{13}C\{^{1}H\}$ NMR spectrum of **G2-cone**, CD_3OD, 298 K, 100 MHz

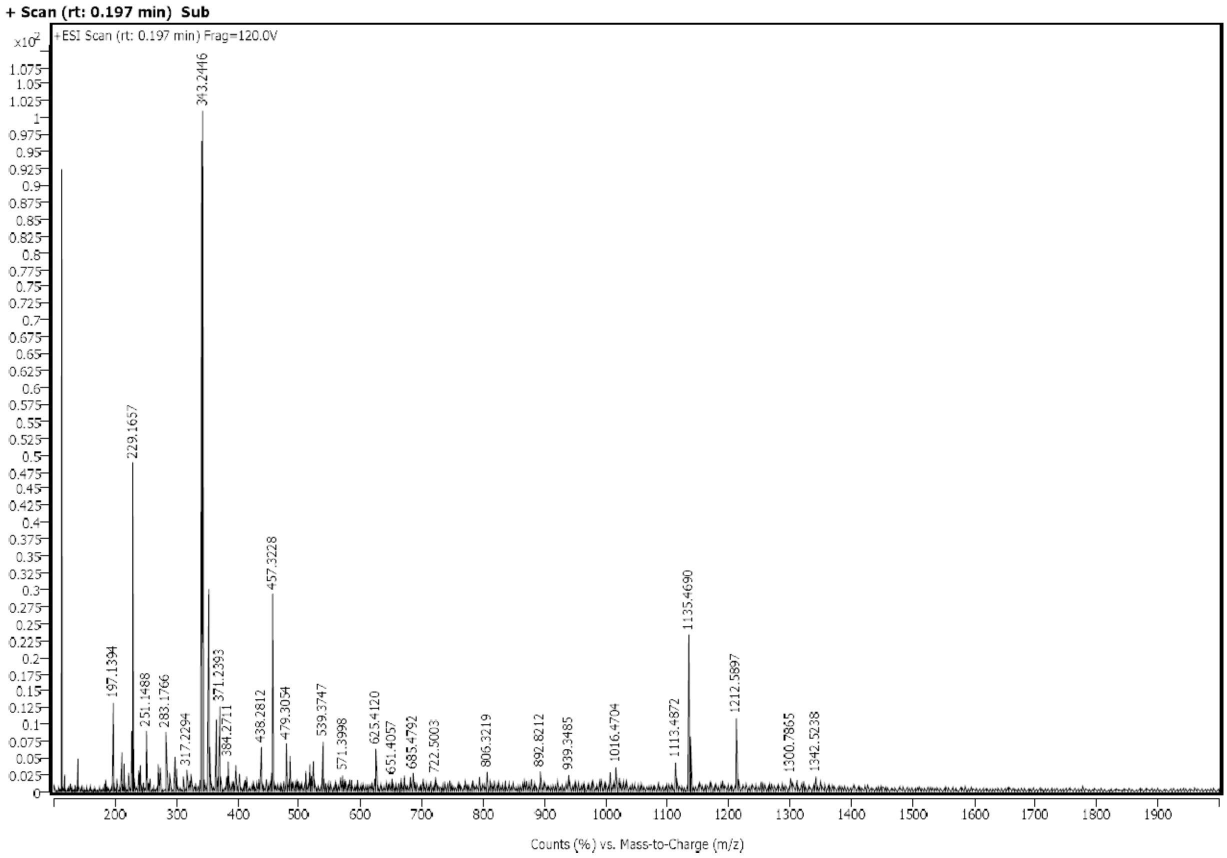

Figure S12. Mass spectrum (HRESI) of **G2-cone**

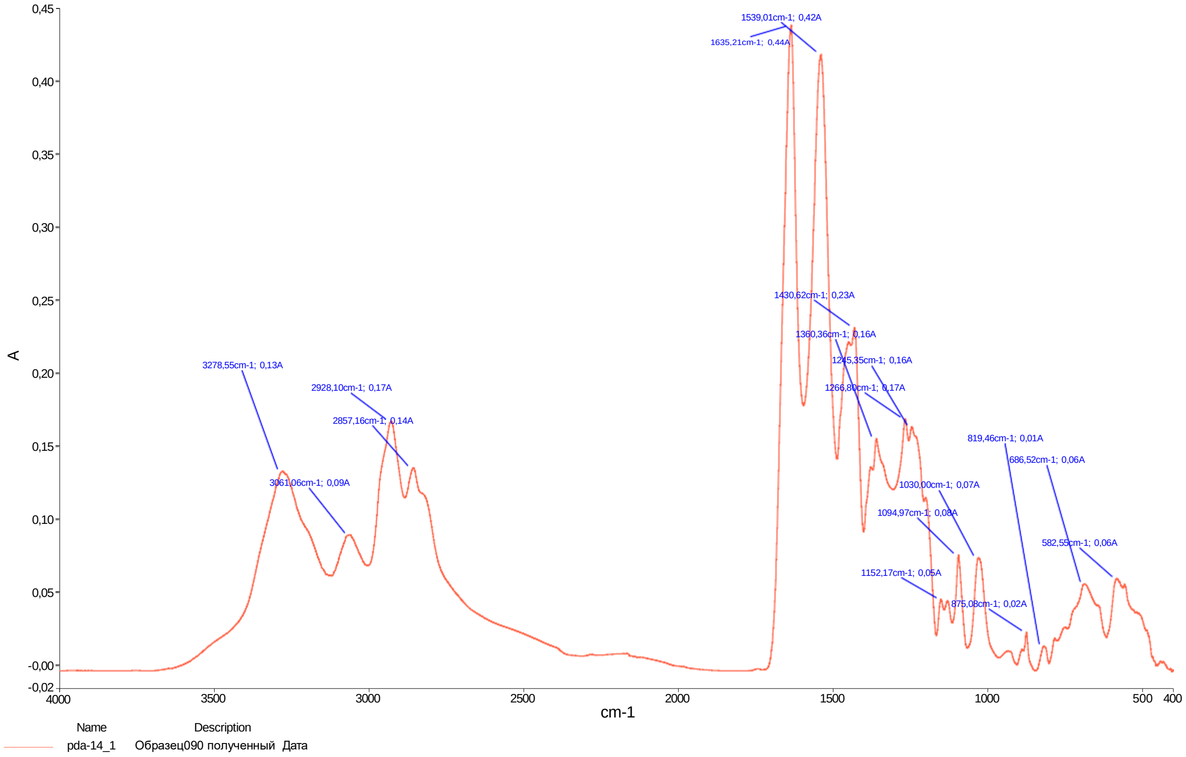

Figure S13. FTIR-ATR spectrum of **G2-cone**

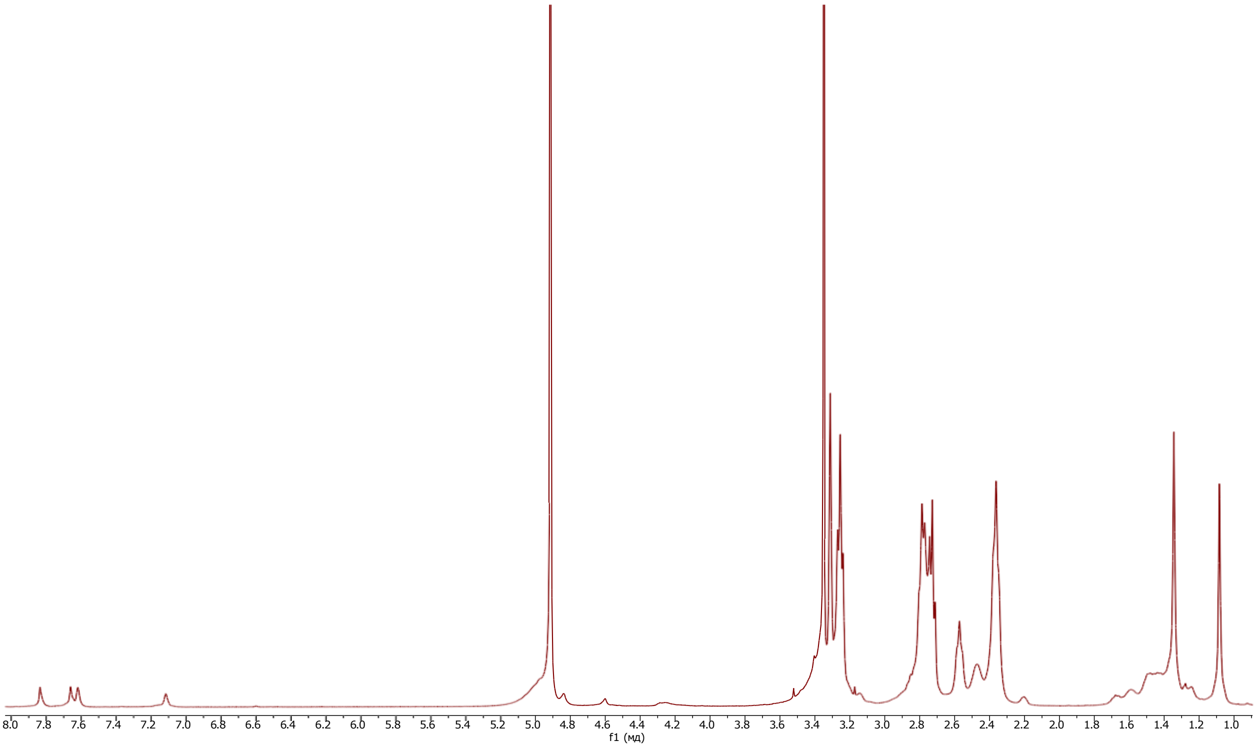

Figure S14. ^{1}H NMR spectrum of **G2-paco**, CD_3OD, 298 K, 400 MHz

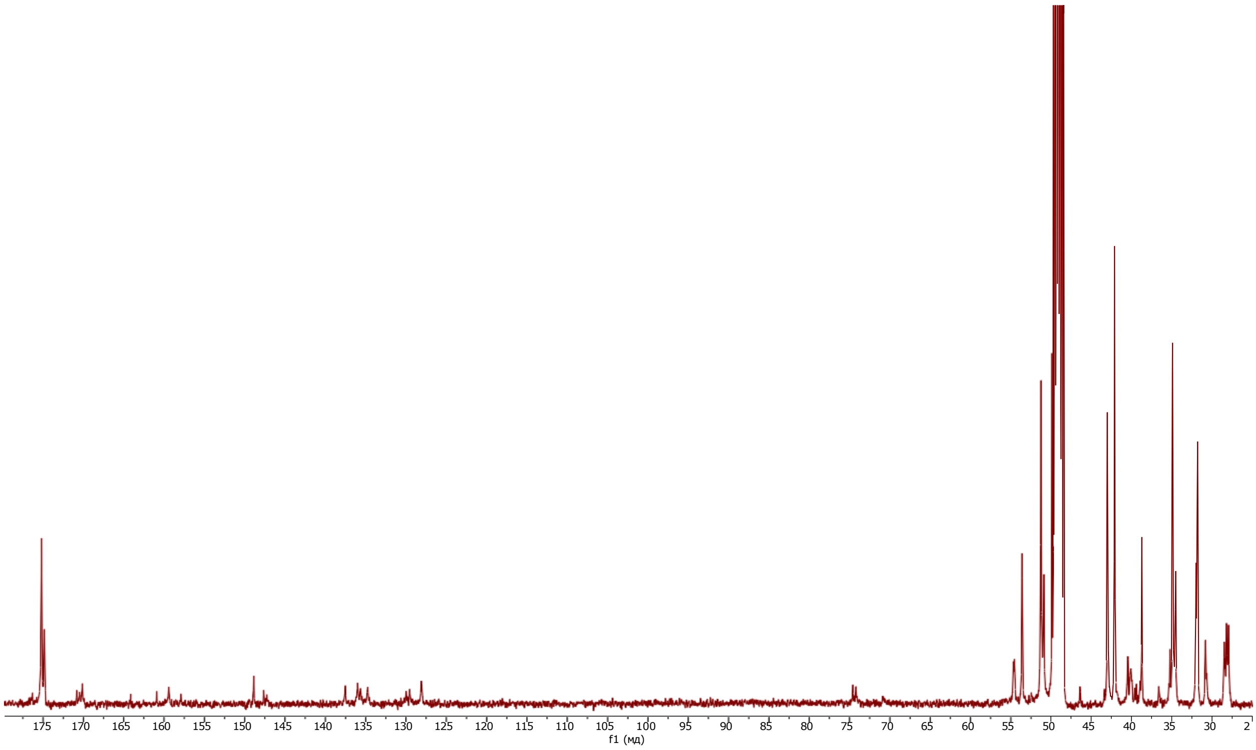

Figure S15. $^{13}C\{^{1}H\}$ NMR spectrum of **G2-paco**, CD_3OD, 298 K, 100 MHz

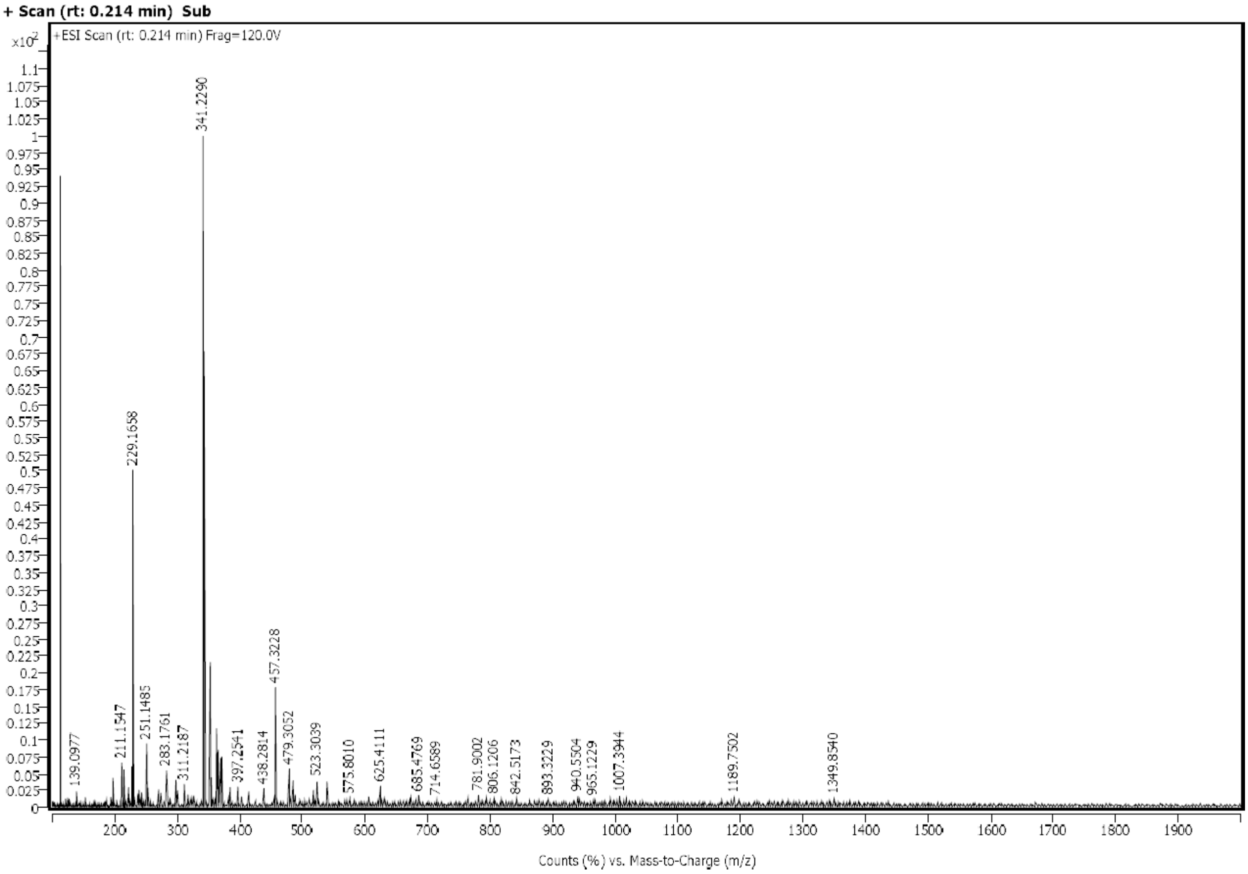

Figure S16. Mass spectrum (HRESI) of **G2-paco**

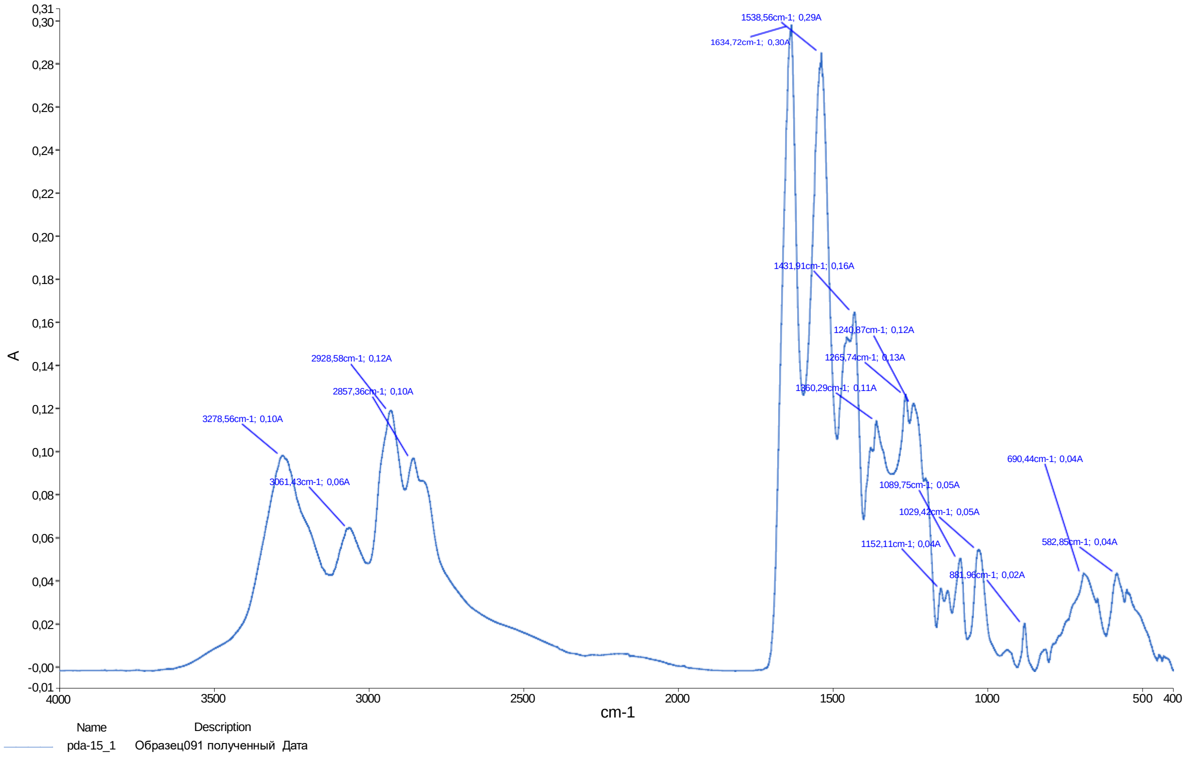

Figure S17. FTIR-ATR spectrum of **G2-paco**

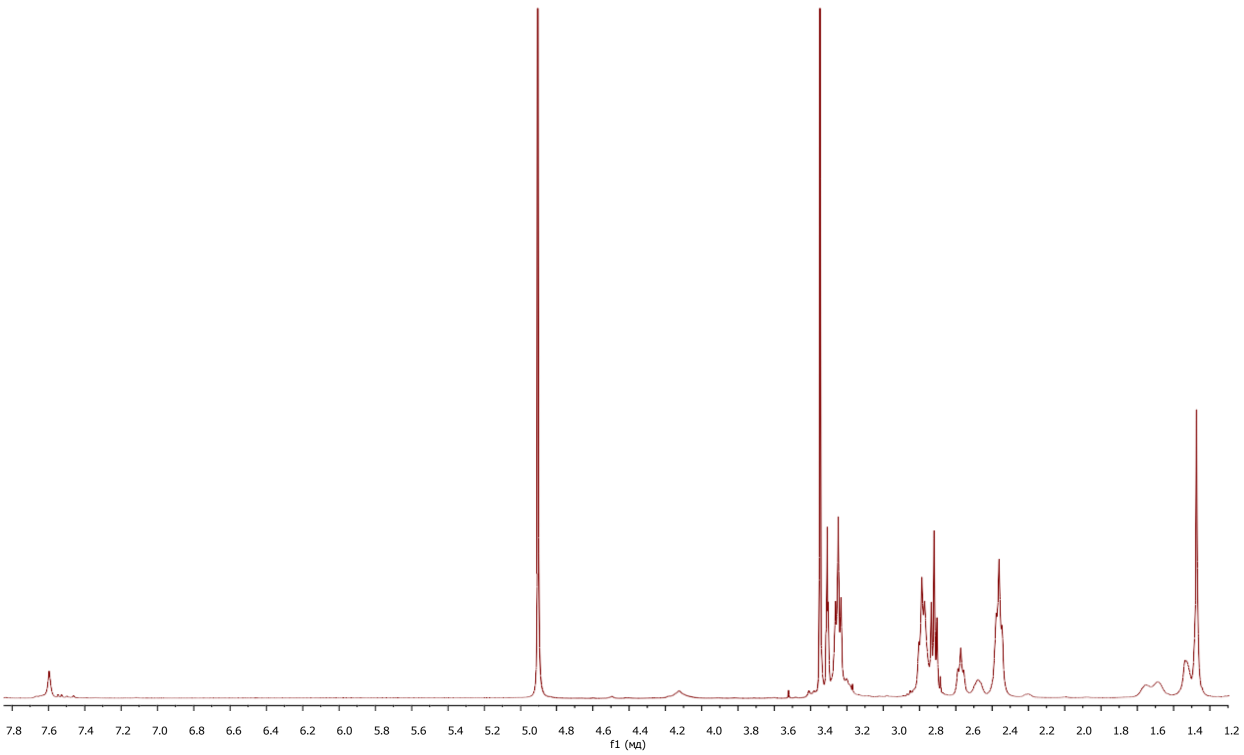

Figure S18. ^{1}H NMR spectrum of **G2-alt**, CD_3OD, 298 K, 400 MHz

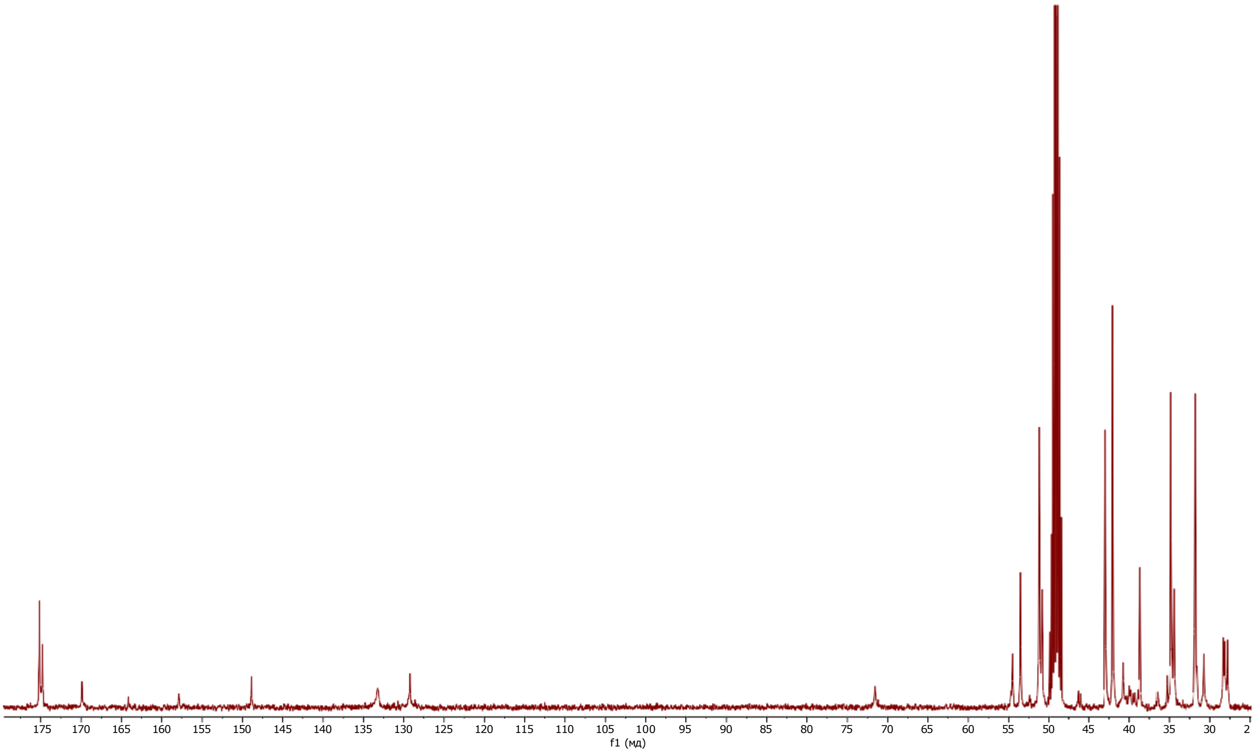

Figure S19. $^{13}C\{^{1}H\}$ NMR spectrum of **G2-alt**, CD_3OD, 298 K, 100 MHz

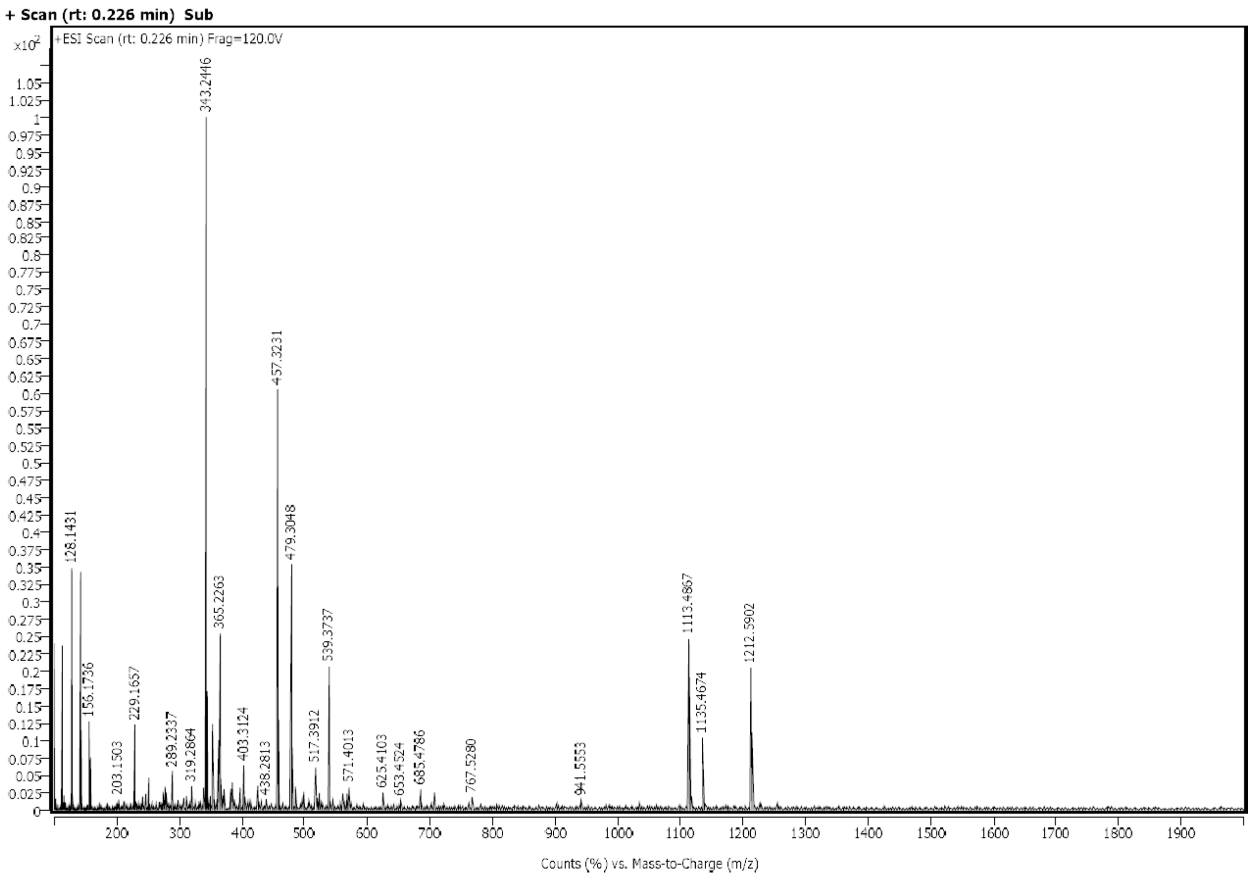

Figure S20. Mass spectrum (HRESI) of **G2-alt**

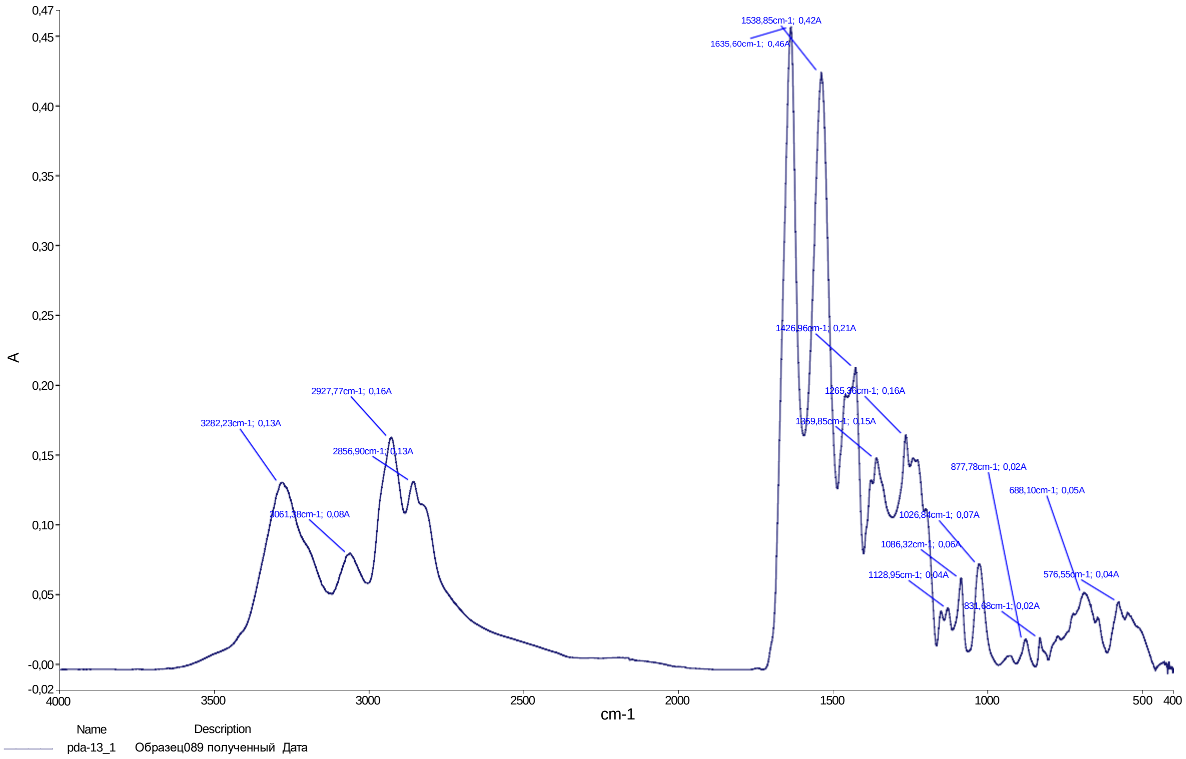

Figure S21. FTIR-ATR spectrum of **G2-alt**

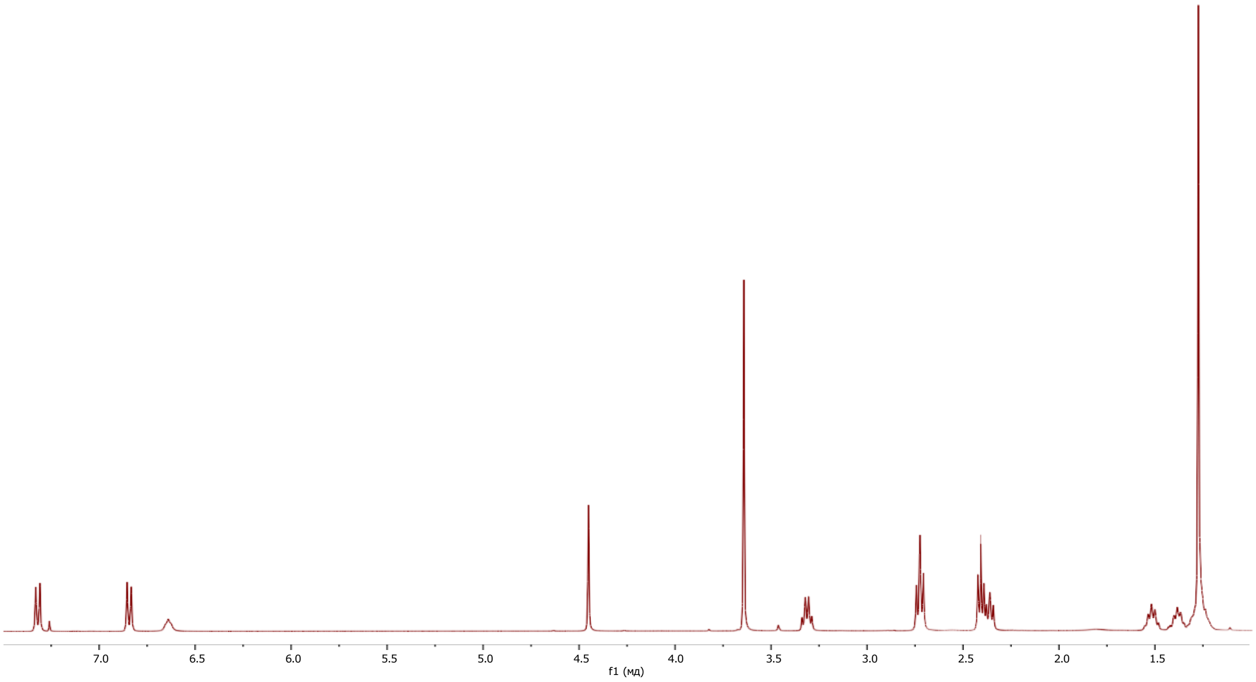

Figure S22. ^{1}H NMR spectrum of **G0.5-monomer**, $CDCl_3$, 298 K, 400 MHz

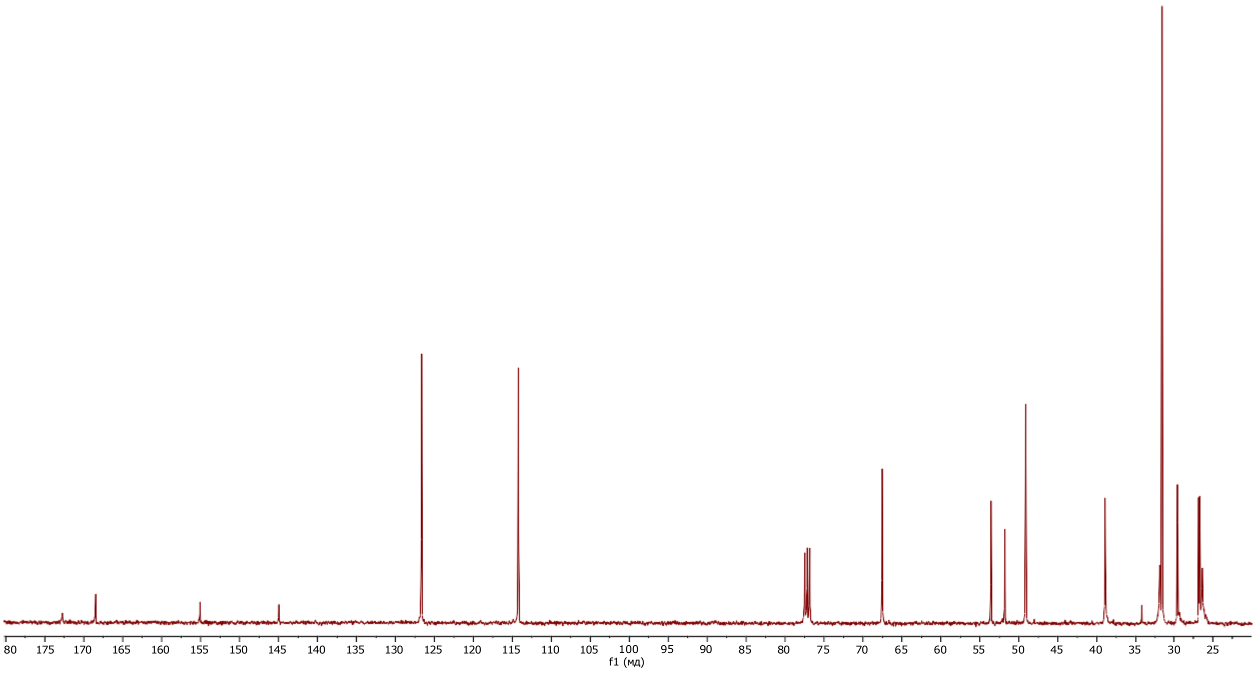

Figure S23. $^{13}C\{^{1}H\}$ NMR spectrum of **G0.5-monomer**, $CDCl_3$, 298 K, 100 MHz

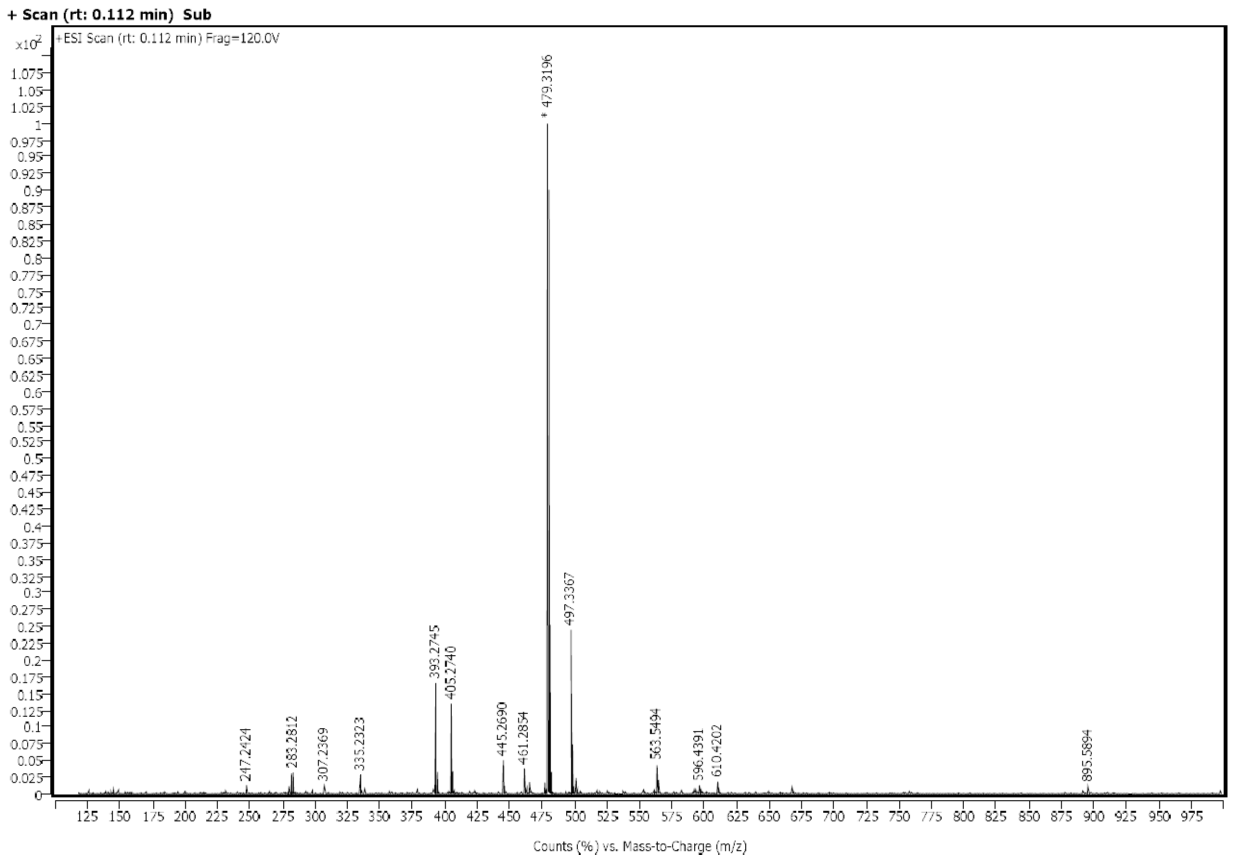

Figure S24. Mass spectrum (HRESI) of **G0.5-monomer**

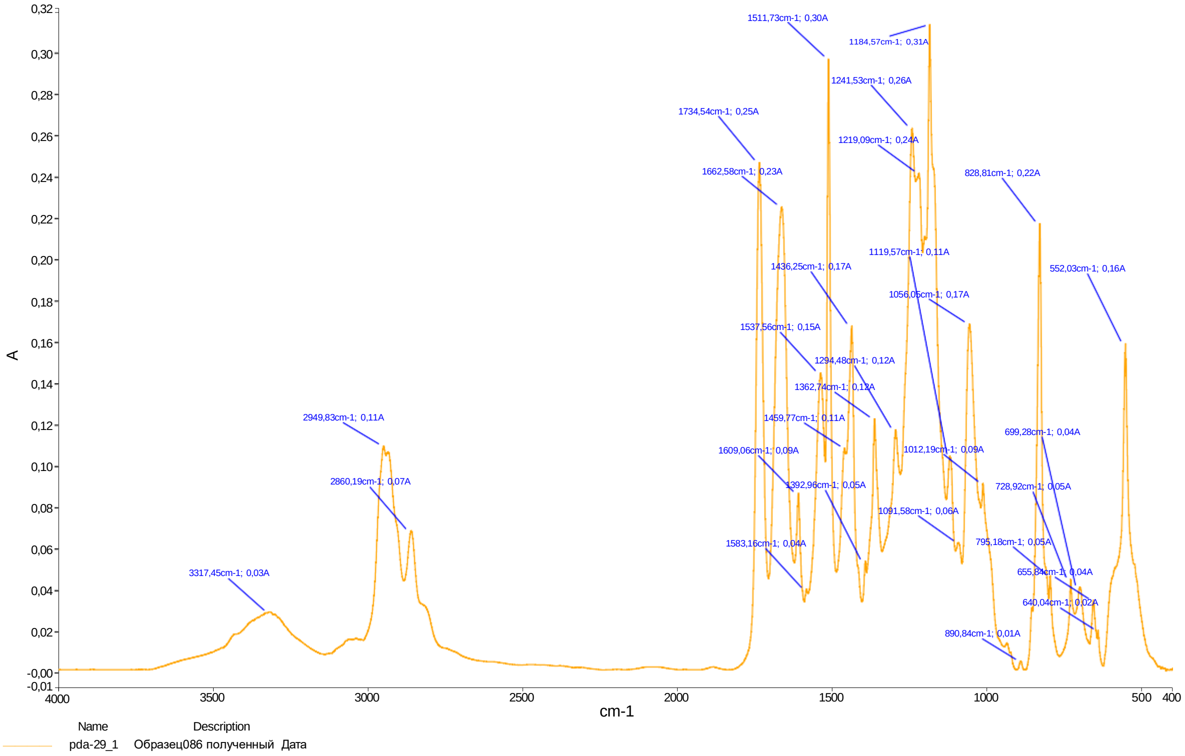

Figure S25. FTIR-ATR spectrum of **G0.5-monomer**

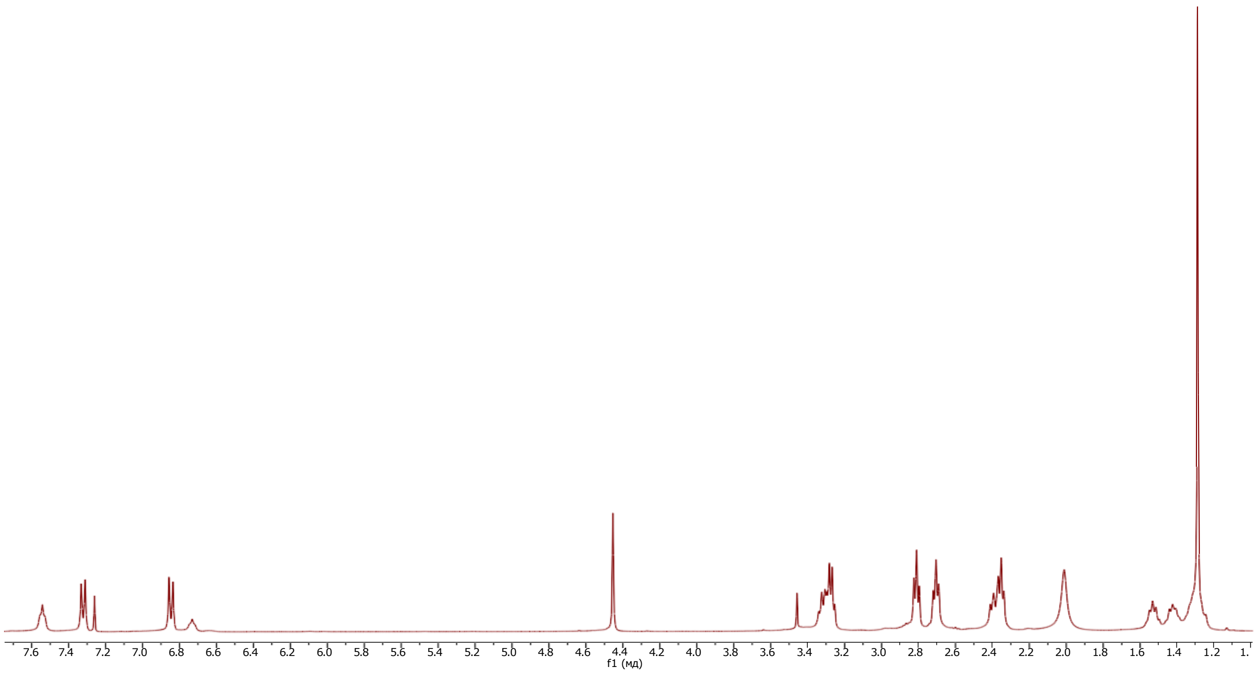

Figure S26. ^{1}H NMR spectrum of **G1-monomer**, $CDCl_3$, 298 K, 400 MHz

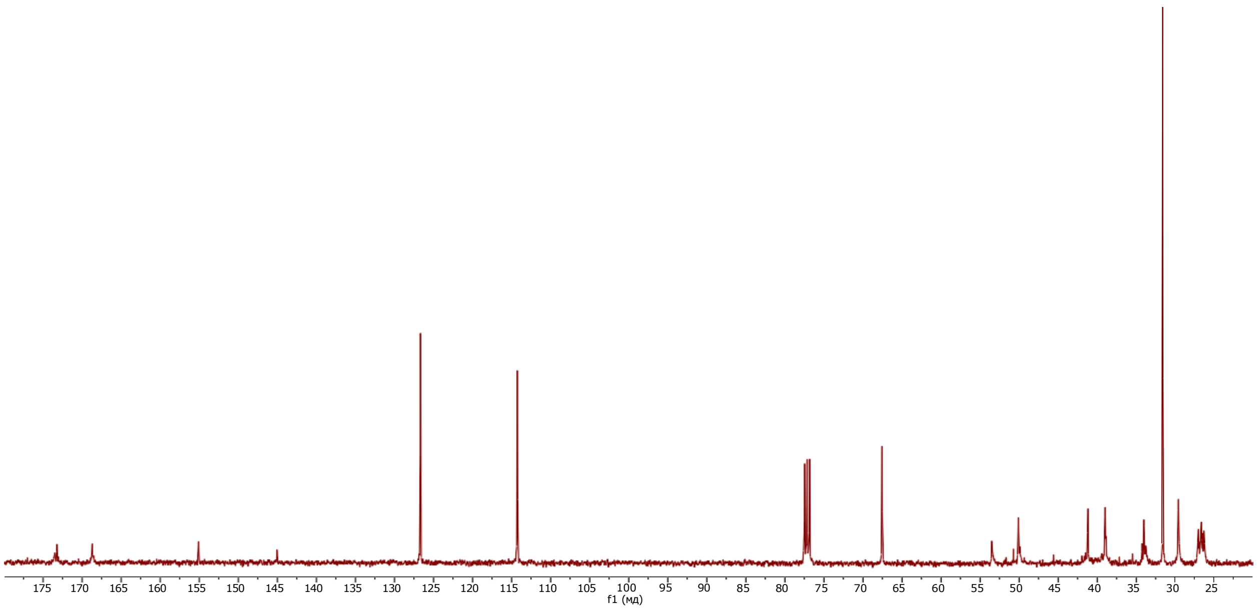

Figure S27. $^{13}C\{^{1}H\}$ NMR spectrum of **G1-monomer**, $CDCl_3$, 298 K, 100 MHz

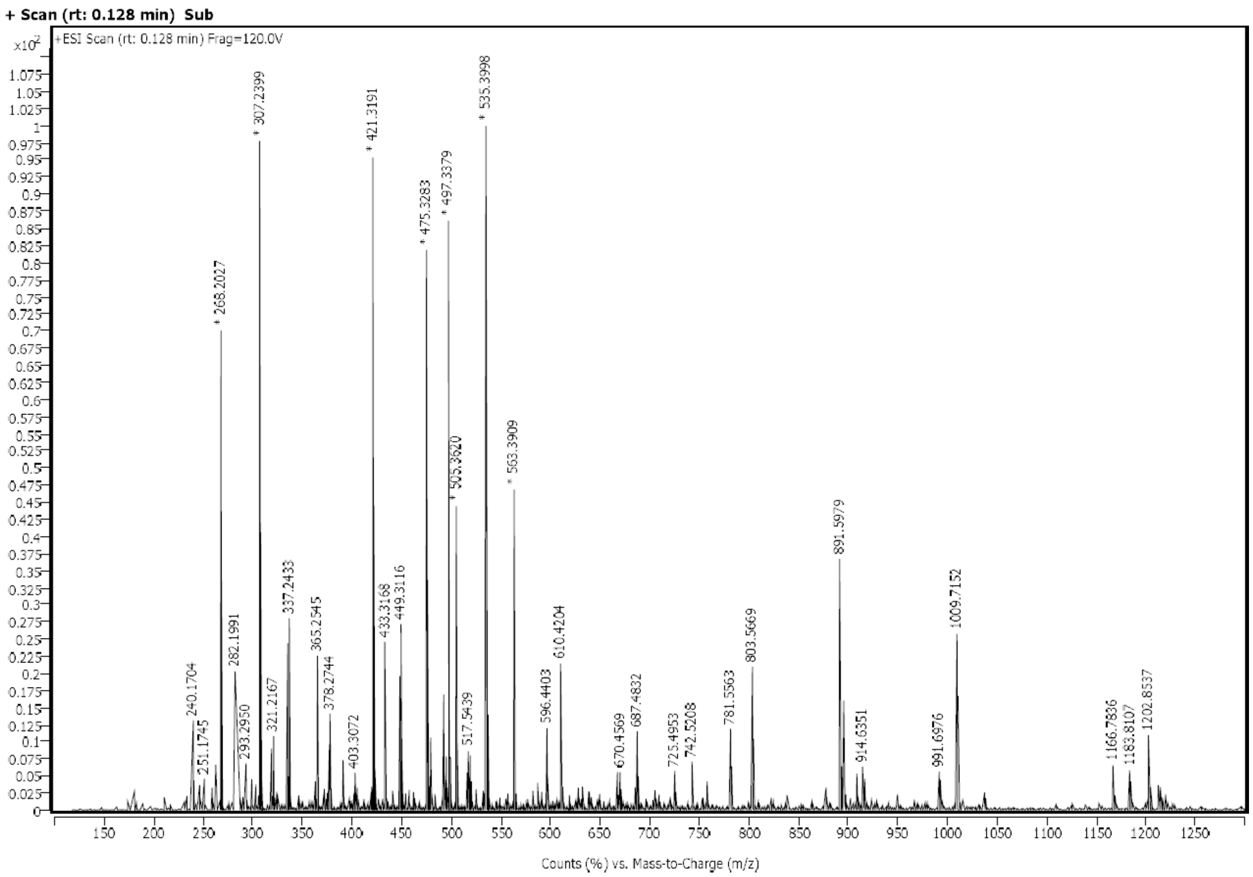

Figure S28. Mass spectrum (HRESI) of **G1-monomer**

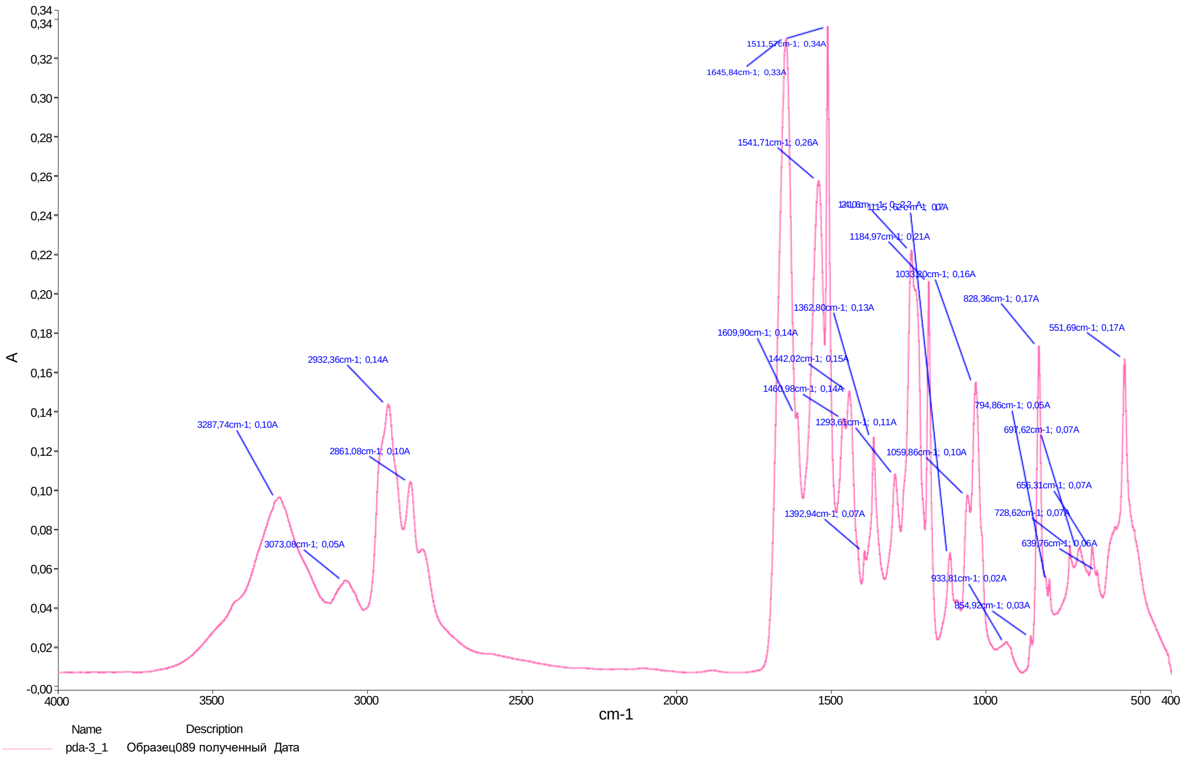

FFigure S29. FTIR-ATR spectrum of **G1-monomer**

2. Spectral properties of the synthesized compounds

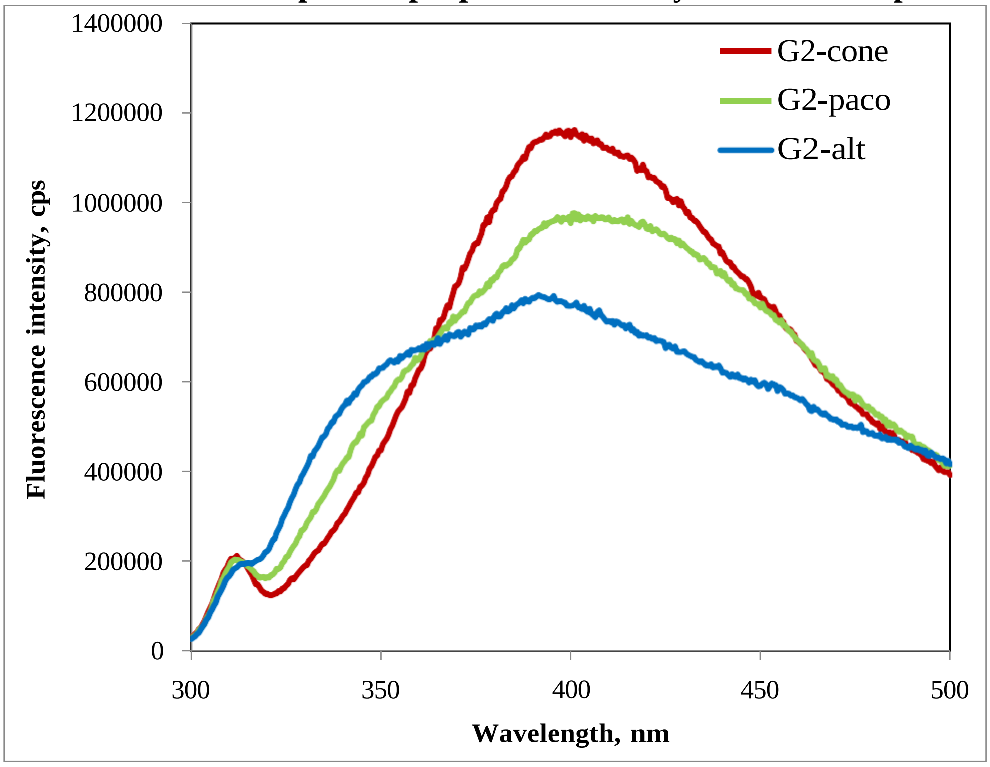

Figure S30. Fluorescence spectra of 5×10^{-5} M **G2** PAMAM-calix-dendrimers in water.

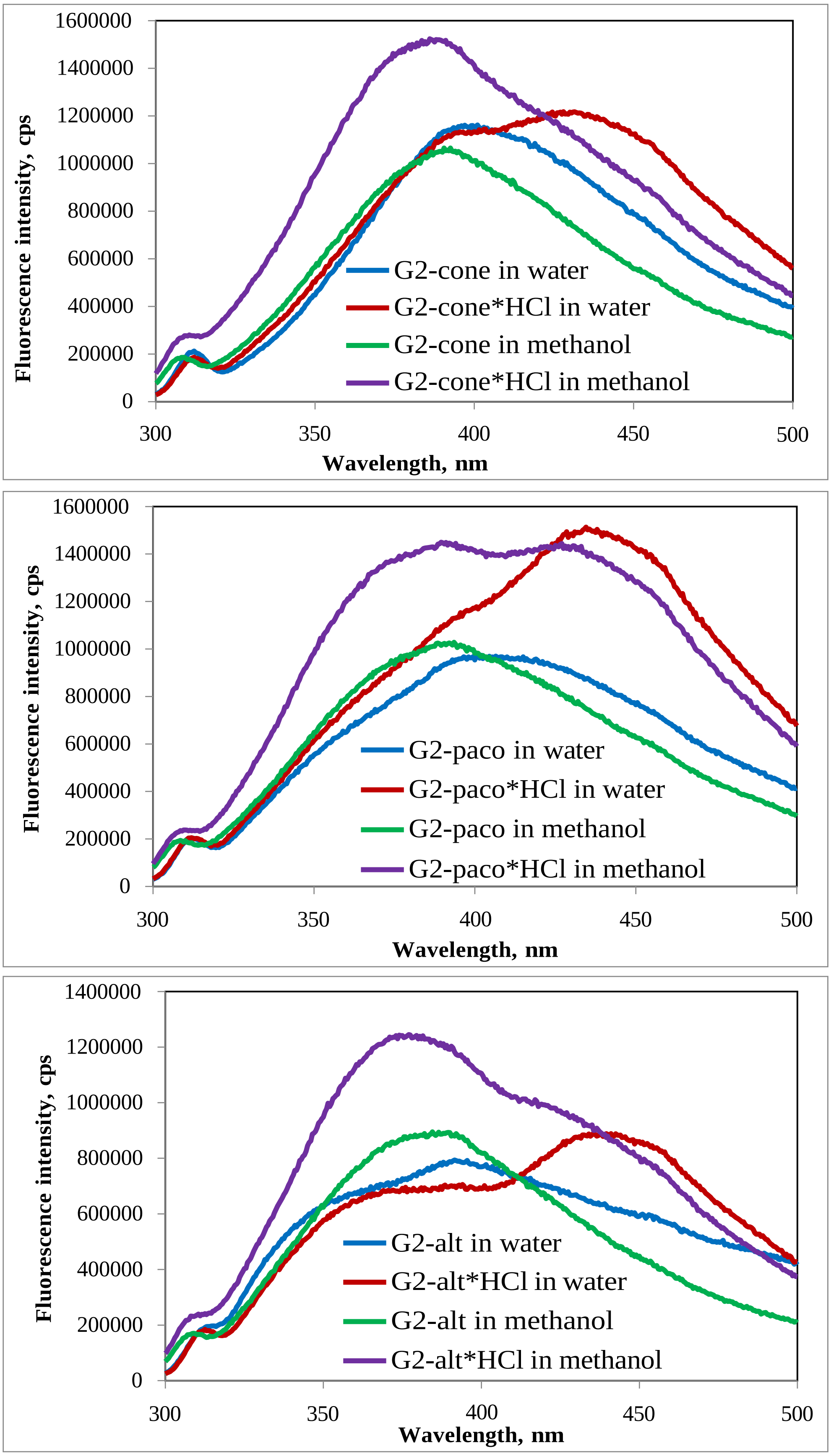

Figure S31. Fluorescence spectra of 5×10^{-5} M **G2** PAMAM-calix-dendrimers in different solvents.

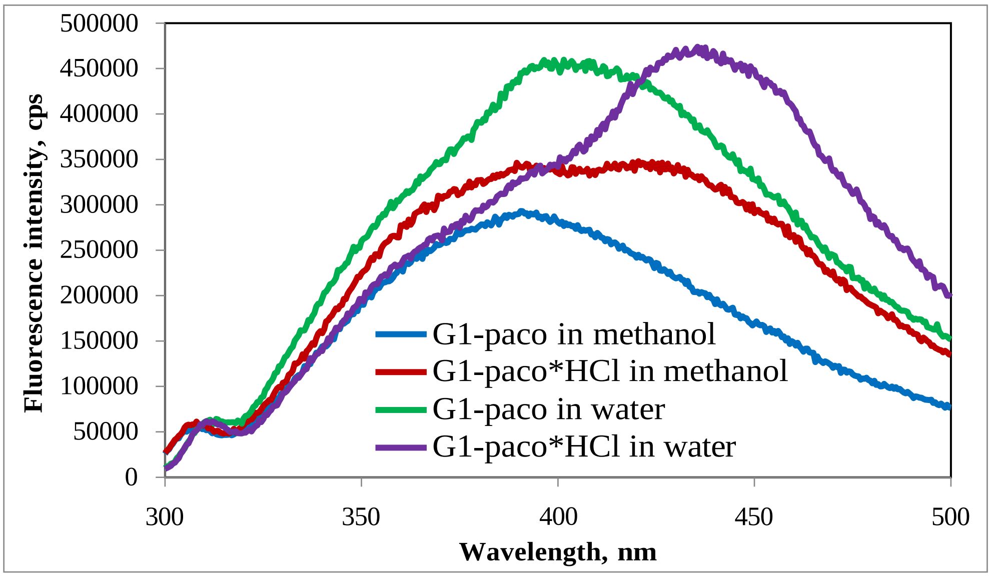

Figure S32. Fluorescence spectra of 5×10^{-5} M **G1-paco** in different solvents.

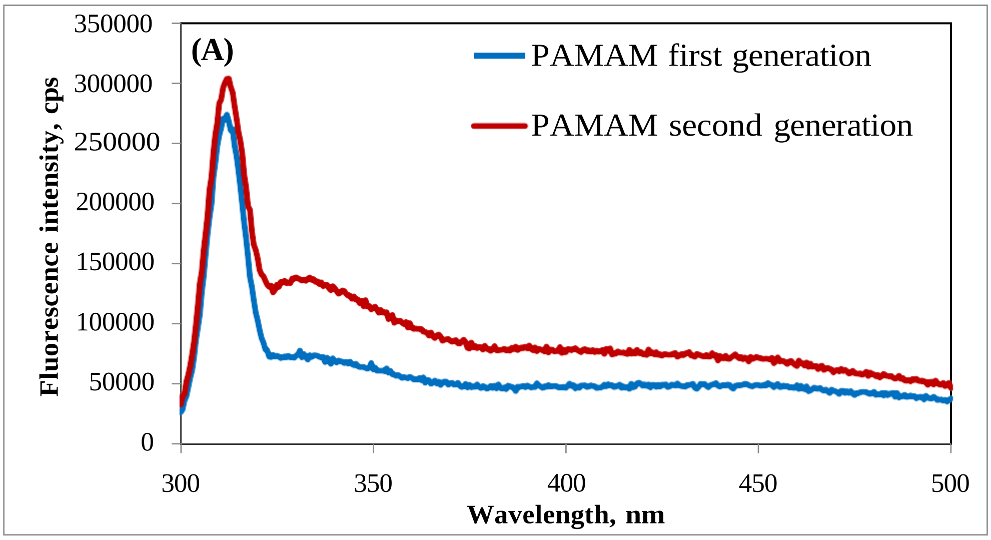

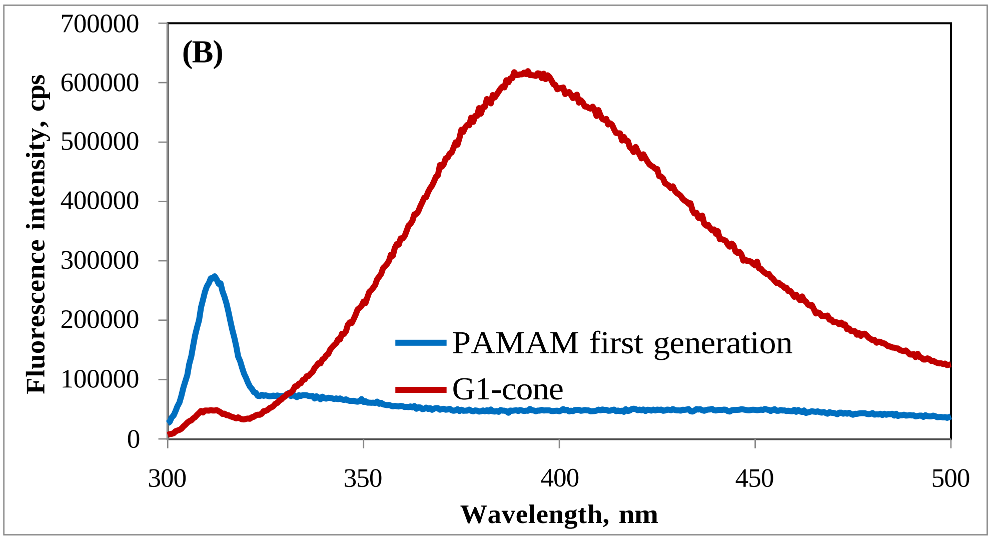

Figure S33. Fluorescence spectra of: (A) PAMAM dendrimers in water (5×10^{-5} M); (B) comparison of spectra of 5×10^{-5} M PAMAM dendrimer first generation with **G1-cone** in water.

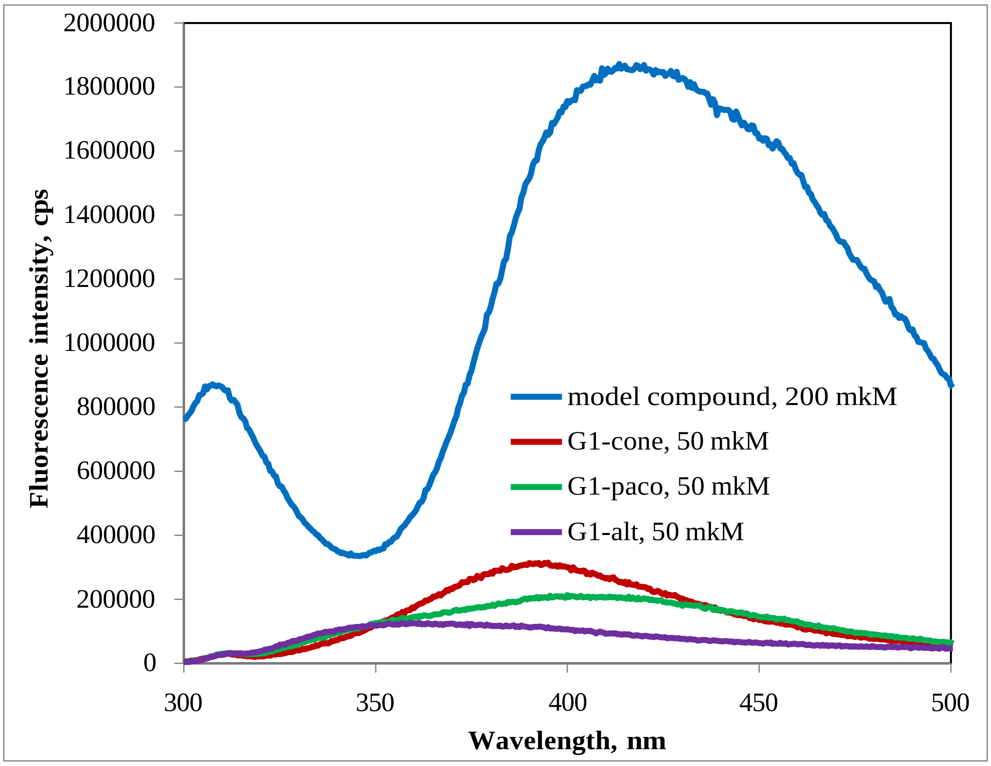

Figure S34. Fluorescence spectra of model compound and **G1** in water.

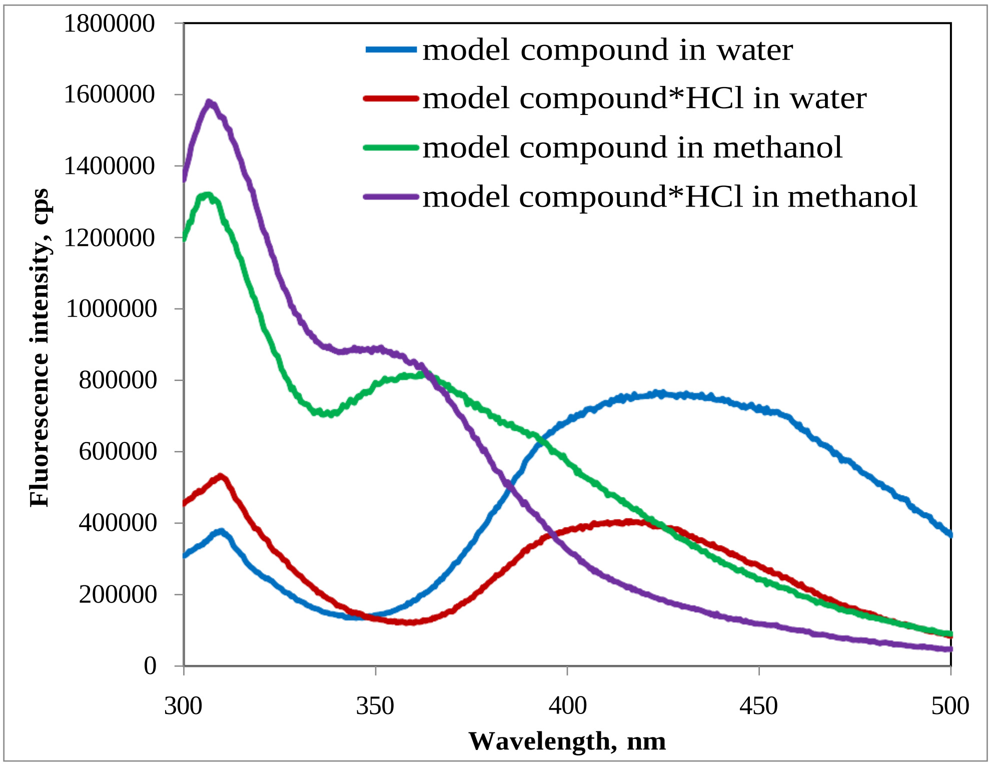

Figure S35. Fluorescence spectra of 200 mkM model compound in different solvents.

3. Complexation investigation

3.1. UV-Vis spectra

UV-Vis spectra were recorded on the Shimadzu UV-3600 spectrophotometer (Kyoto, Japan) using 10 mm quartz cuvette at 293 K. UV-Vis spectra were registered in 50 mM phosphate buffer (pH = 7.4) in 1 h after preparation of the solutions. The concentration of the catecholamines was 50 µM. Concentrations of the **G1** and **G2** dendrimers were 5 µM, concentration of the **G1-monomer** was 20 µM.

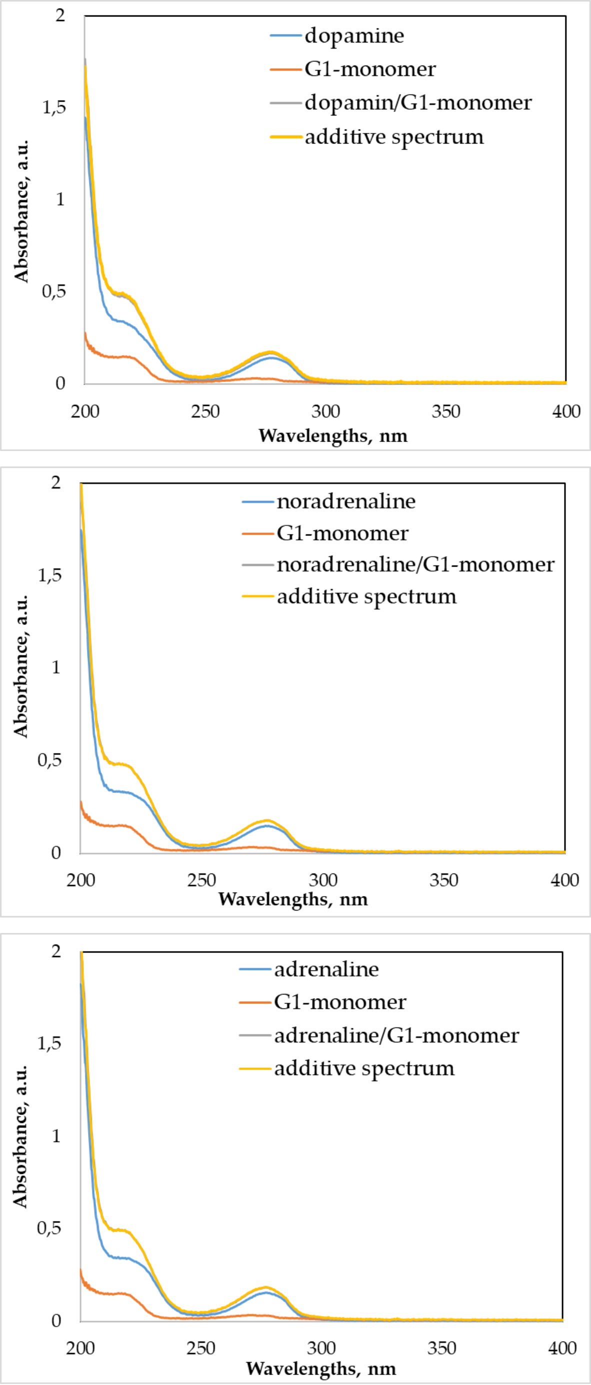

Figure S36. UV-Vis spectra of 50 mkM catecholamines, 20 mkM **G1-monomer** and their mixtures in 50 mM phosphate buffer (pH 7.4).

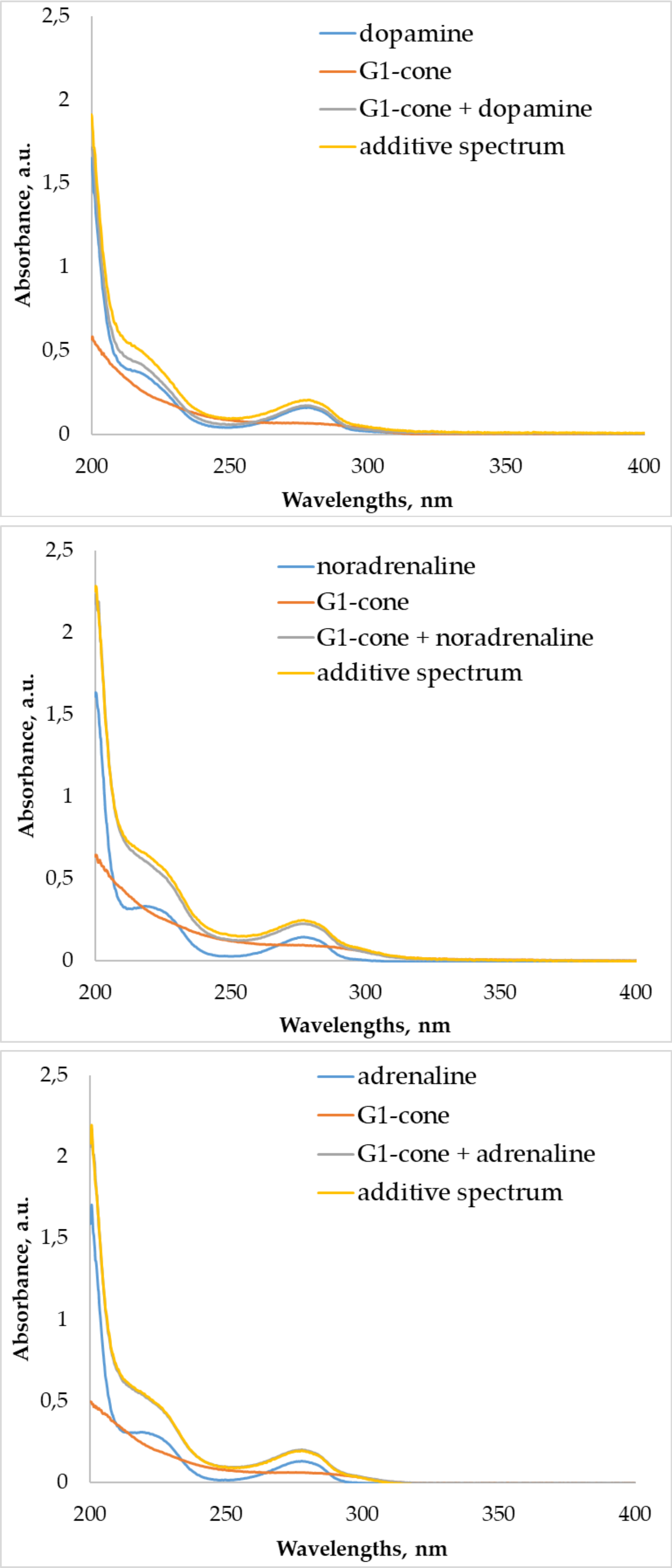

Figure S37. UV-vis spectra of **G1-cone** (5 μM), catecholamines (50 μM) and their mixtures in phosphate buffer, pH = 7.4, 50 mM.

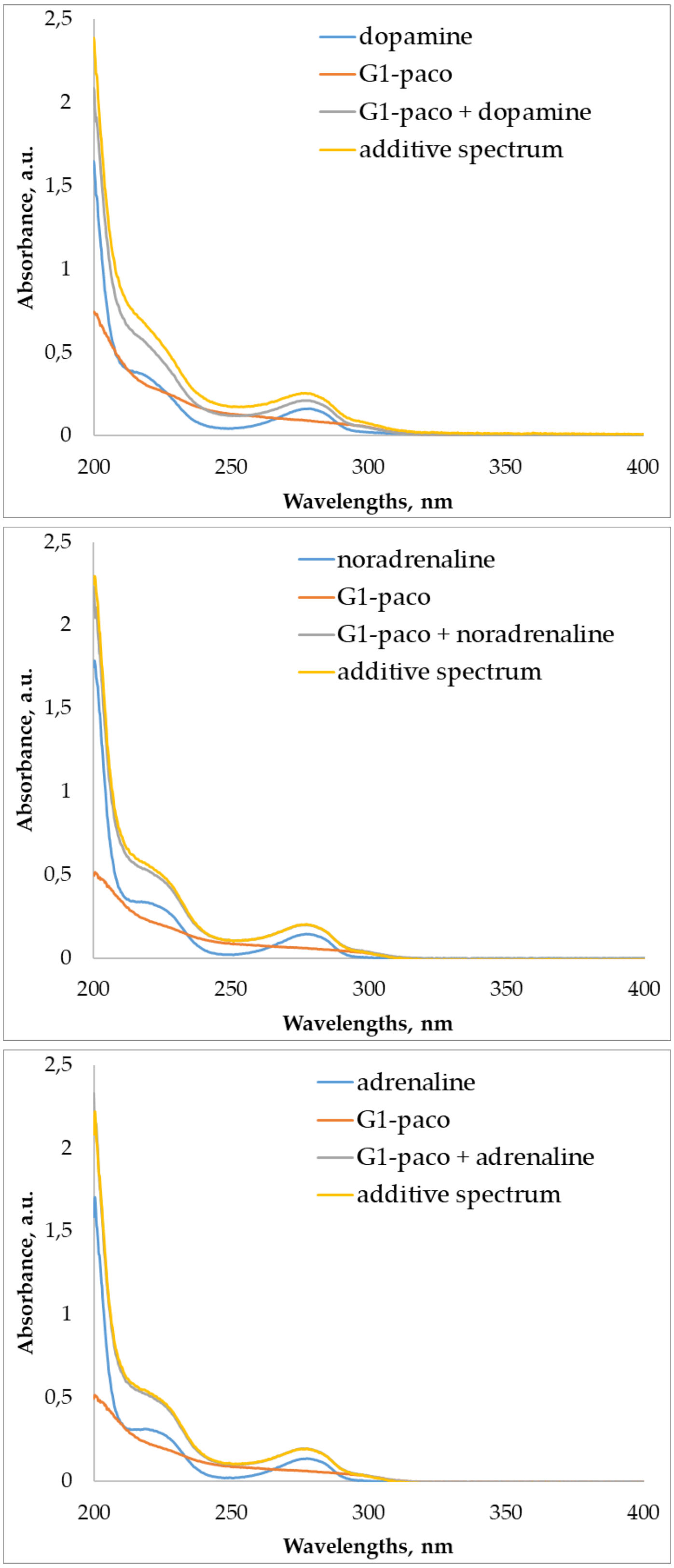

Figure S38. UV-vis spectra of **G1-paco** (5 µM), catecholamines (50 µM) and their mixtures in phosphate buffer, pH = 7.4, 50 mM.

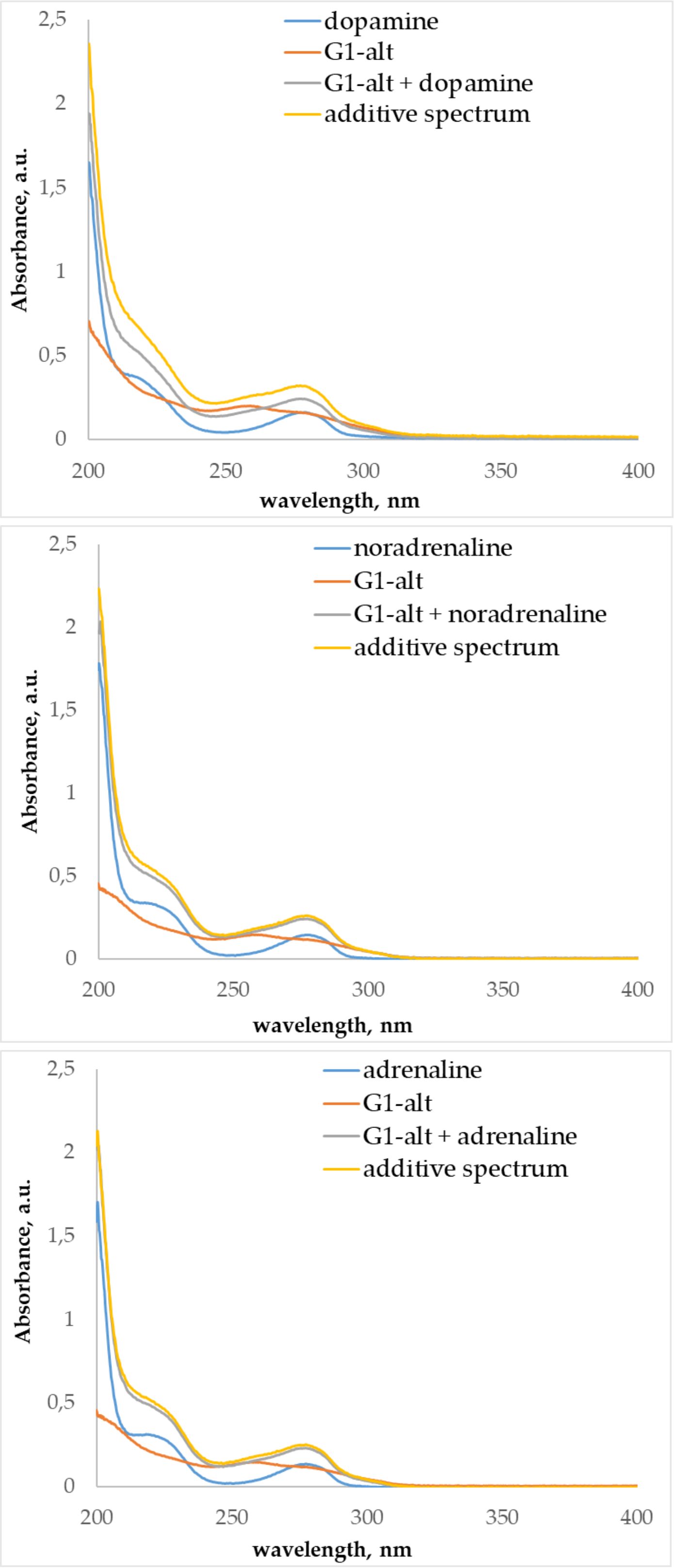

Figure S39. UV-vis spectra of **G1-alt** (5 µM), catecholamines (50 µM) and their mixtures in phosphate buffer, pH = 7.4, 50 mM.

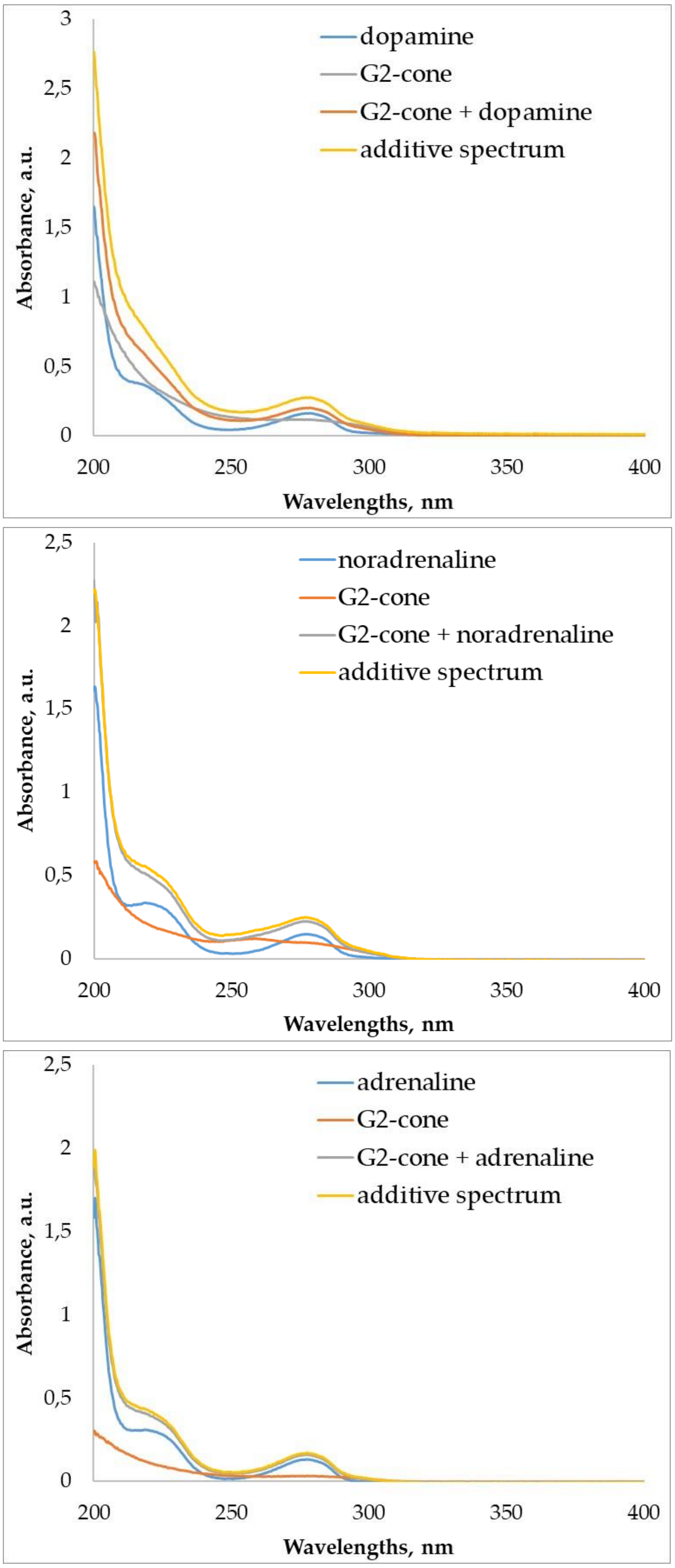

Figure S40. UV-vis spectra of **G2-cone** (5 µM), catecholamines (50 µM) and their mixtures in phosphate buffer, pH = 7.4, 50 mM.

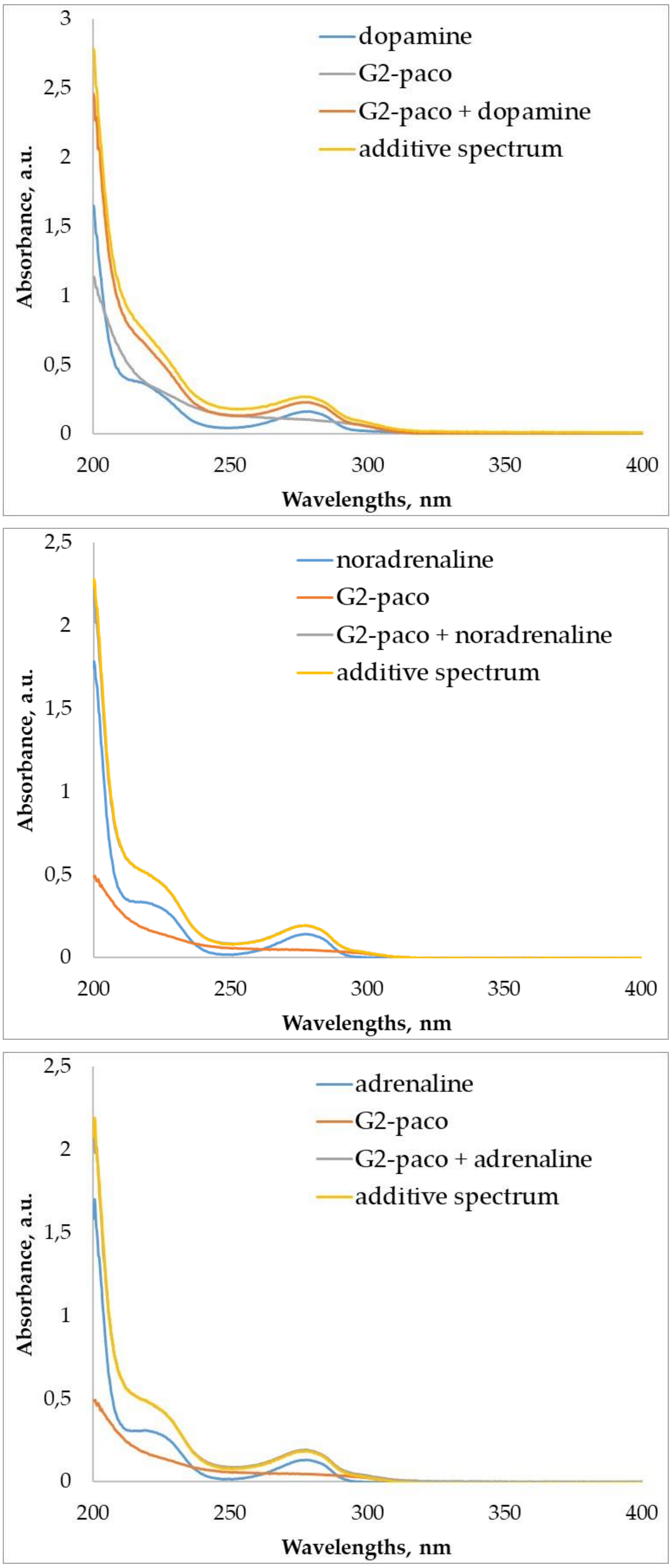

Figure S41. UV-vis spectra of **G2-paco** (5 µM), catecholamines (50 µM) and their mixtures in phosphate buffer, pH = 7.4, 50 mM.

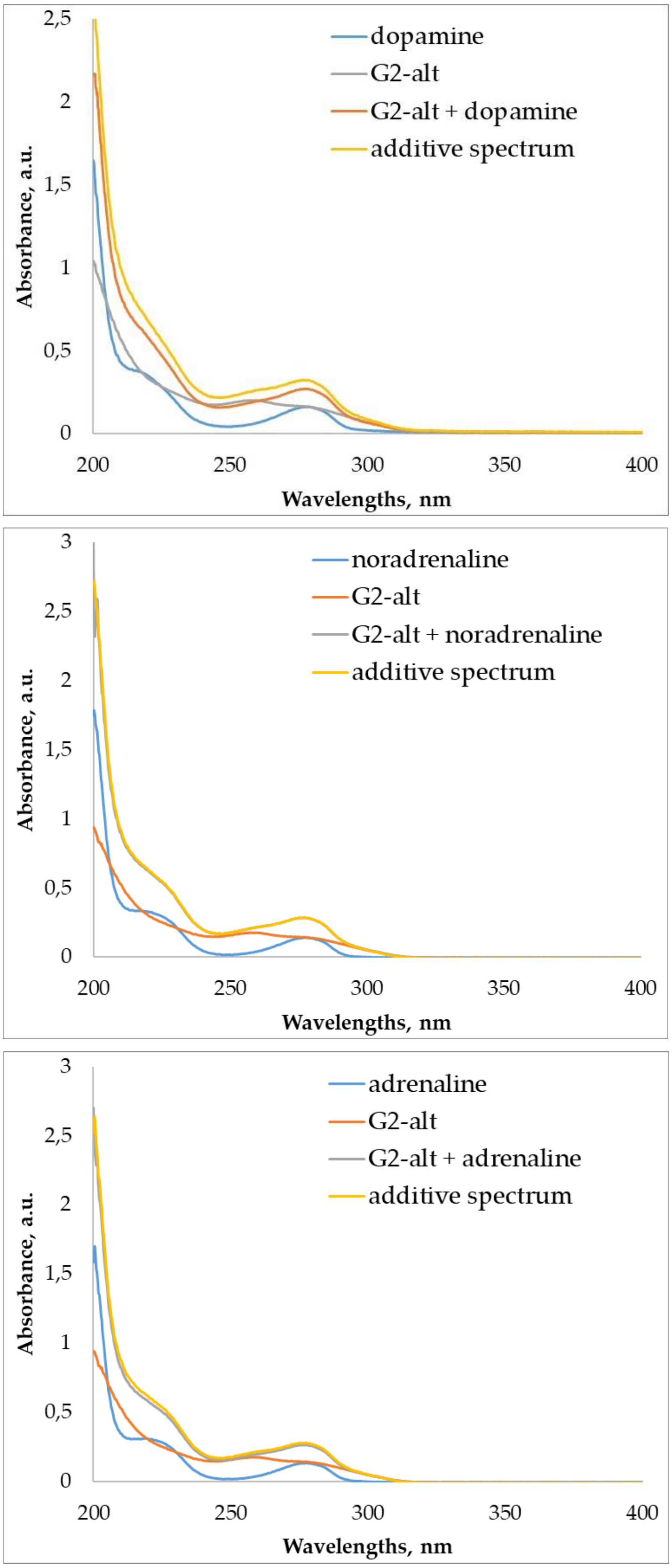

Figure S42. UV-vis spectra of **G2-alt** (5 µM), catecholamines (50 µM) and their mixtures in phosphate buffer, pH = 7.4, 50 mM.

3.2. Fluorescence spectra

Fluorescence spectra were recorded on the Fluorolog 3 luminescent spectrometer (Horiba Jobin Yvon, Longjumeau, France). The excitation wavelength was selected at 280 nm. The emission scan range was 300–550 nm. Excitation and emission slits were 7 nm. Quartz cuvettes with an optical path length of 10 mm were used. The cuvette was placed at the front face position to avoid inner filter effect. Fluorescence spectra were automatically corrected by the Fluorescence software. Spectra were recorded at 293 K in 50 mM phosphate buffer (pH = 7.4). The concentration of the catecholamines was 10 µM. The concentration of the **G1** and **G2** dendrimers was ranged from 0 to 90 µM. Concentration of the **G1-monomer** was 100 µM.

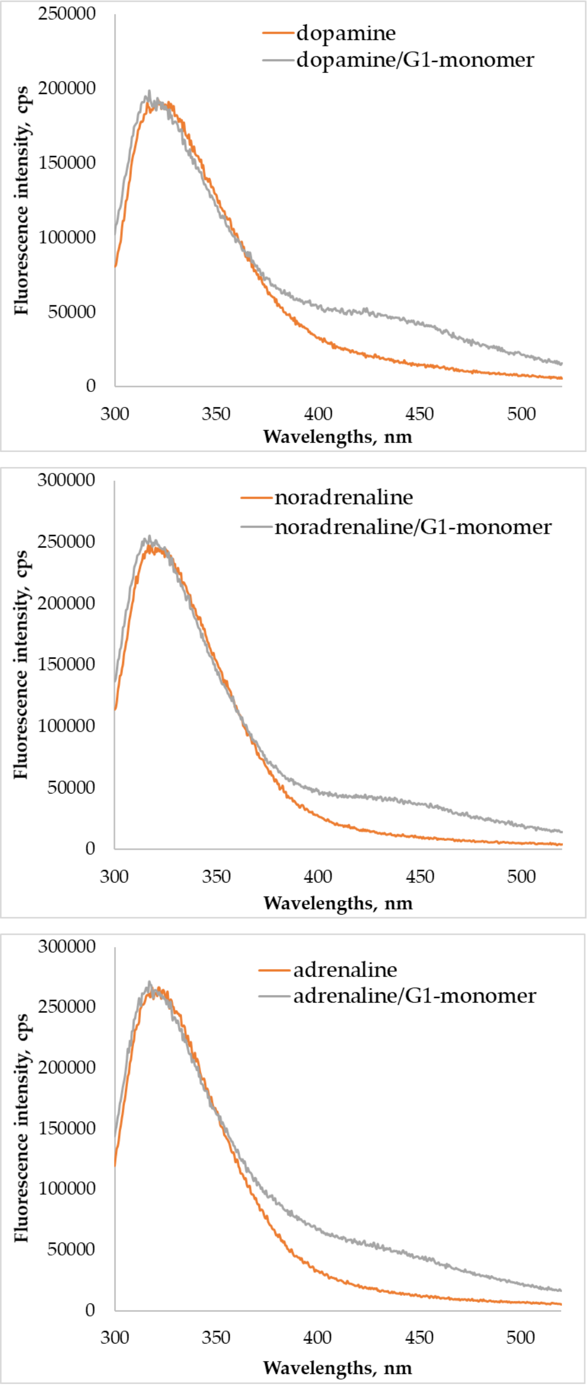

Figure S43. Fluorescence spectra of 10 mkM catecholamines in the presence of 100 mkM **G1-monomer** in 50 mM phosphate buffer (pH 7.4).

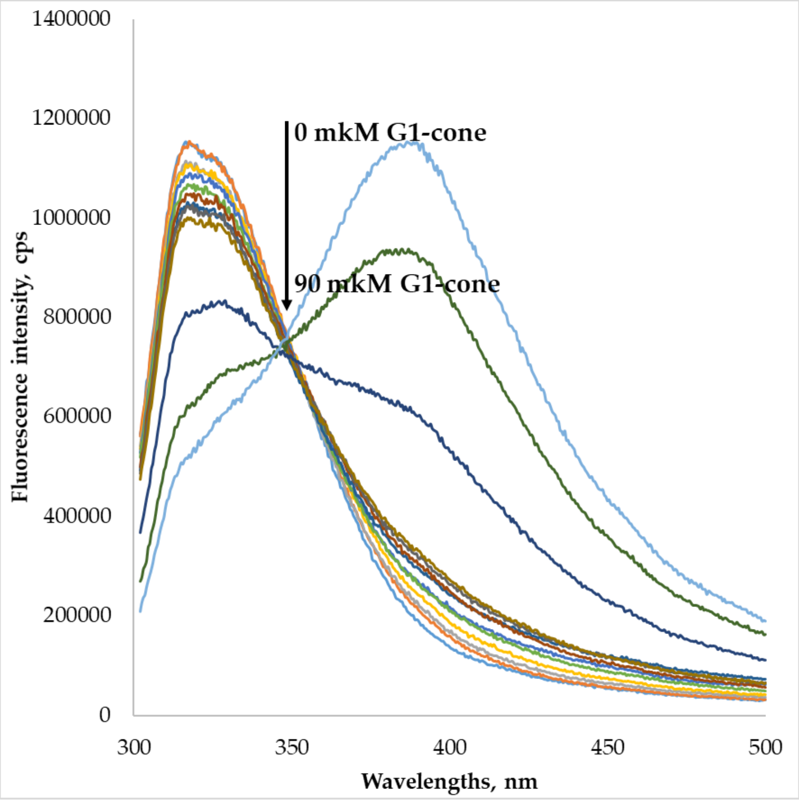

Figure S44. Fluorescence spectra of dopamine (100 μM) in presence of different concentrations of G1-cone (0-90 μM) in phosphate buffer, pH = 7.4, 50 mM.

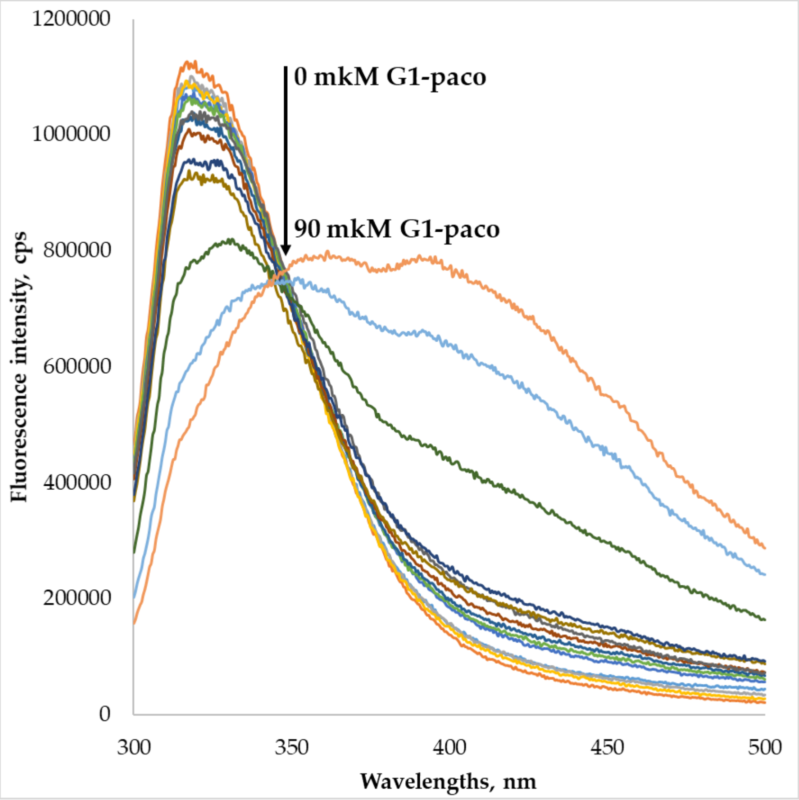

Figure S45. Fluorescence spectra of dopamine (100 μM) in presence of different concentrations of G1-paco (0-90 μM) in phosphate buffer, pH = 7.4, 50 mM.

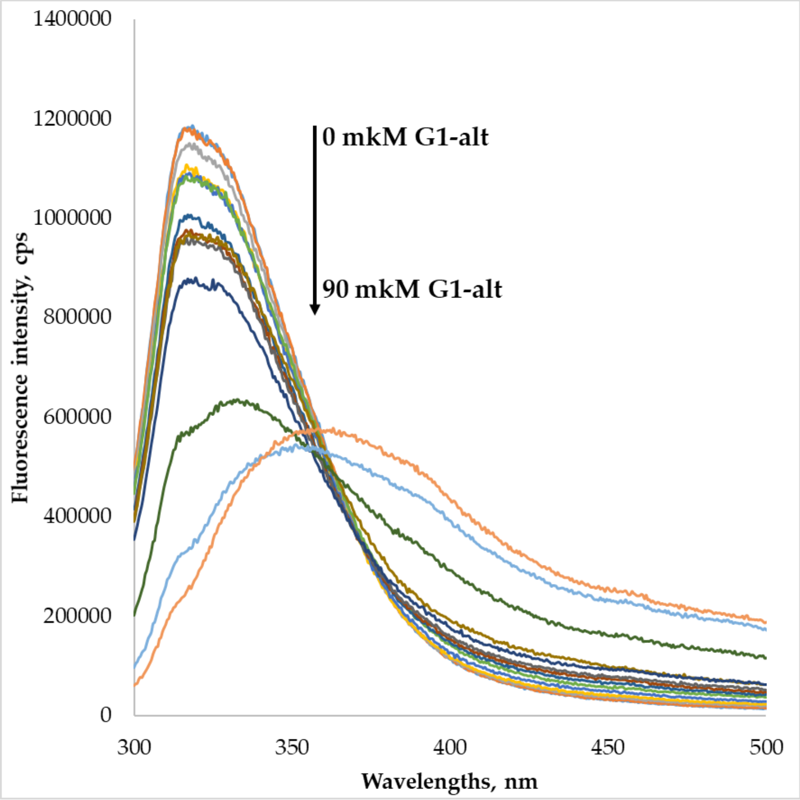

Figure S46. Fluorescence spectra of dopamine (100 μM) in presence of different concentrations of G1-alt (0-90 μM) in phosphate buffer, pH = 7.4, 50 mM.

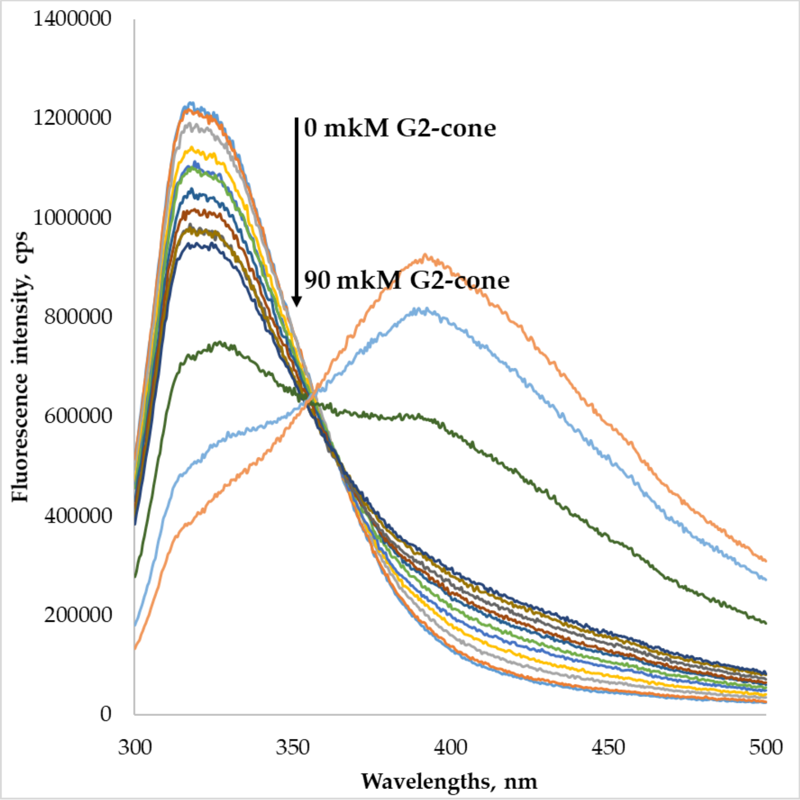

Figure S47. Fluorescence spectra of dopamine (100 μM) in presence of different concentrations of G2-cone (0-90 μM) in phosphate buffer, pH = 7.4, 50 mM.

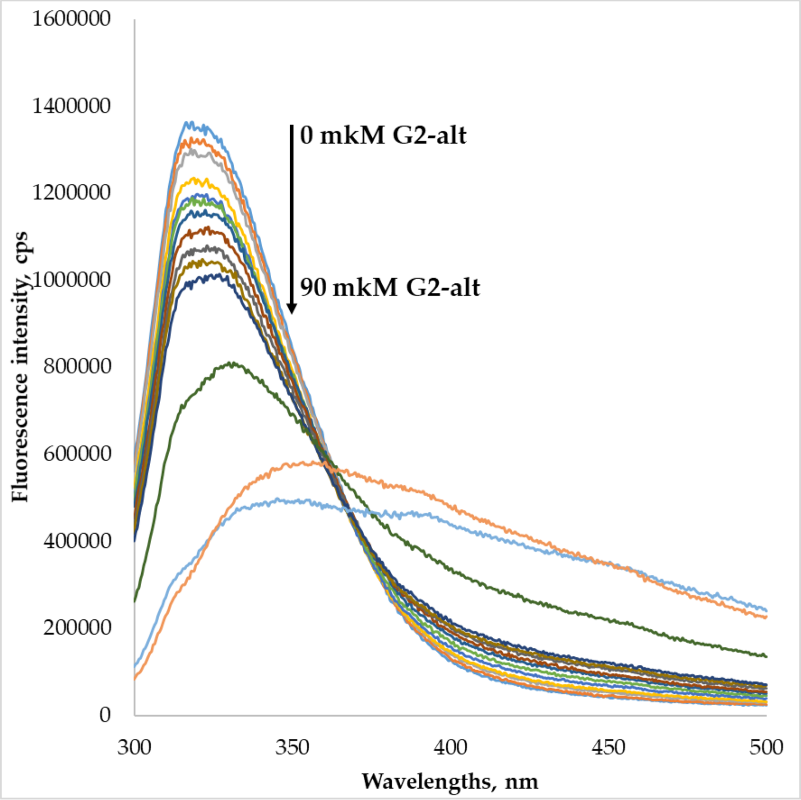

Figure S48. Fluorescence spectra of dopamine (100 μM) in presence of different concentrations of G2-alt (0-90 μM) in phosphate buffer, pH = 7.4, 50 mM.

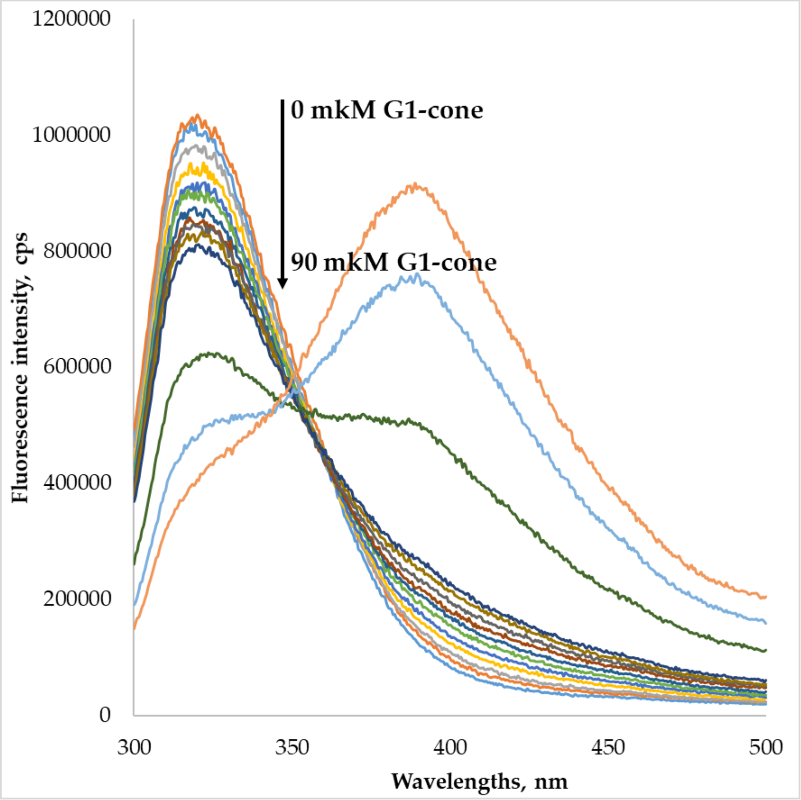

Figure S49. Fluorescence spectra of noradrenaline (100 μM) in presence of different concentrations of G1-cone (0-90 μM) in phosphate buffer, pH = 7.4, 50 mM.

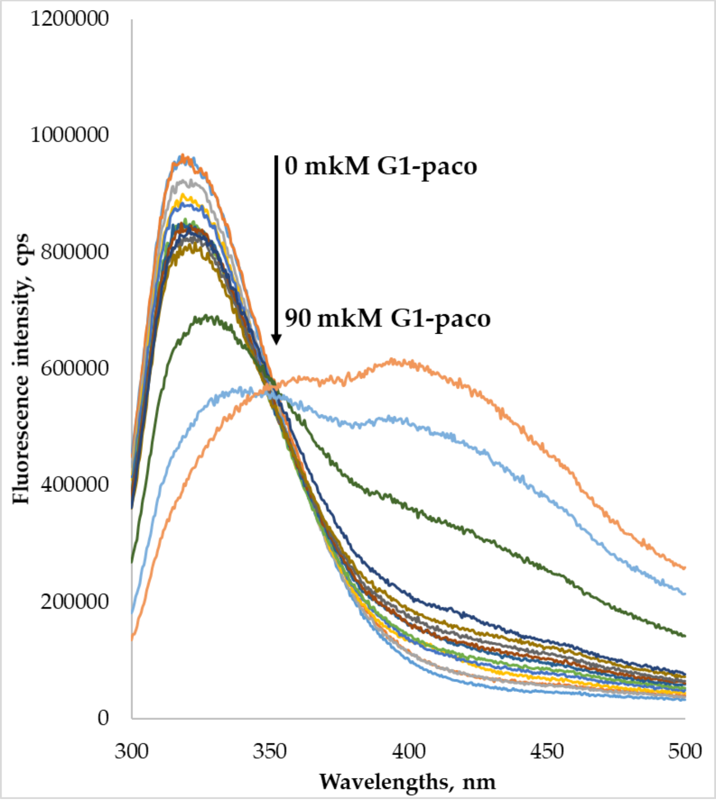

Figure S50. Fluorescence spectra of noradrenaline (100 μM) in presence of different concentrations of G1-paco (0-90 μM) in phosphate buffer, pH = 7.4, 50 mM.

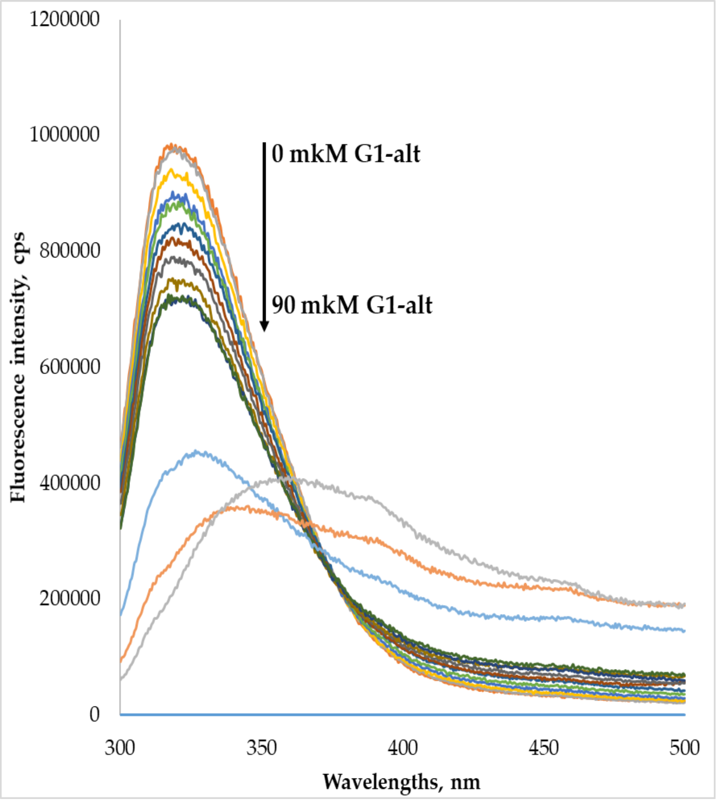

Figure S51. Fluorescence spectra of noradrenaline (100 μM) in presence of different concentrations of G1-alt (0-90 μM) in phosphate buffer, pH = 7.4, 50 mM.

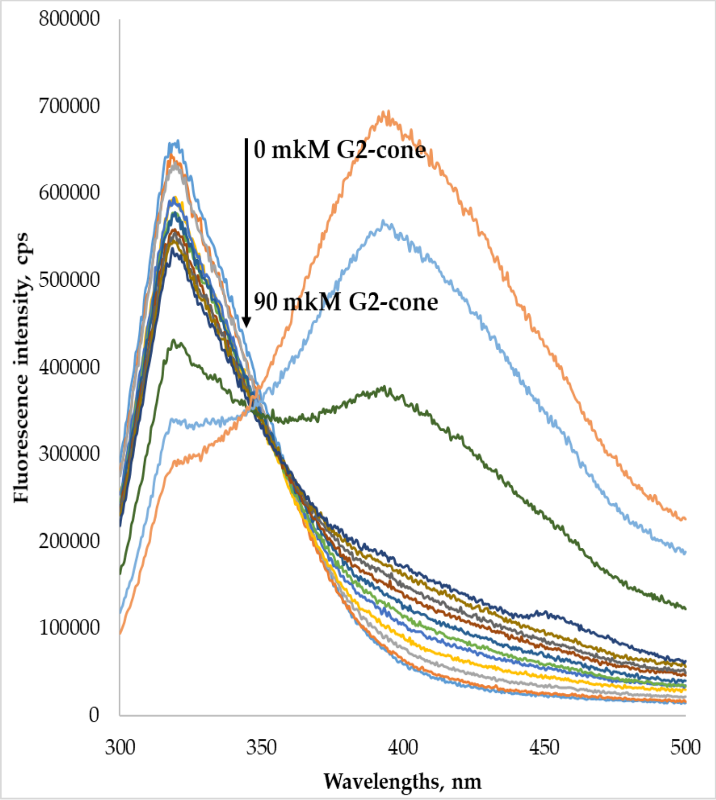

Figure S52. Fluorescence spectra of noradrenaline (100 μM) in presence of different concentrations of G2-cone (0-90 μM) in phosphate buffer, pH = 7.4, 50 mM.

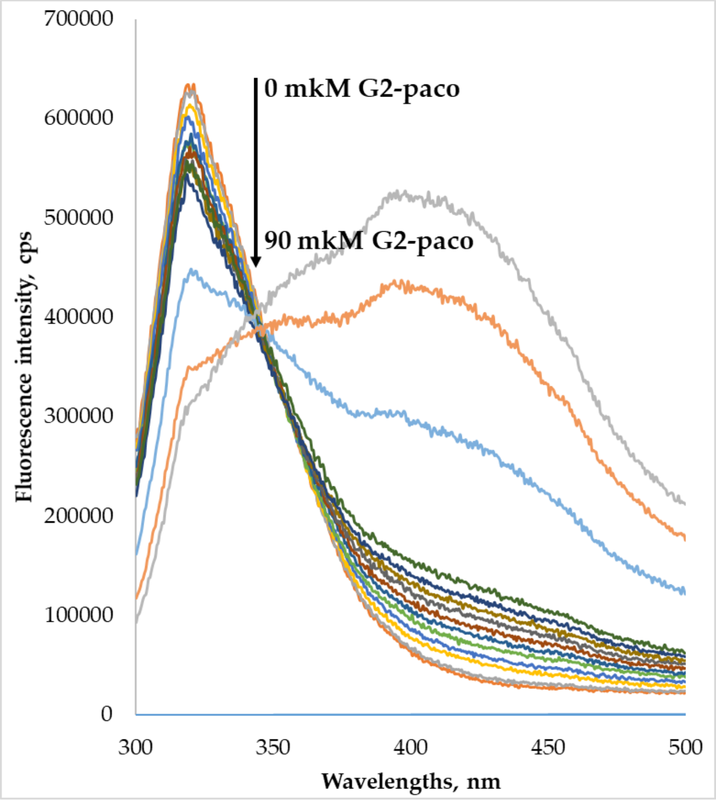

Figure S53. Fluorescence spectra of noradrenaline (100 μM) in presence of different concentrations of G2-paco (0-90 μM) in phosphate buffer, pH = 7.4, 50 mM.

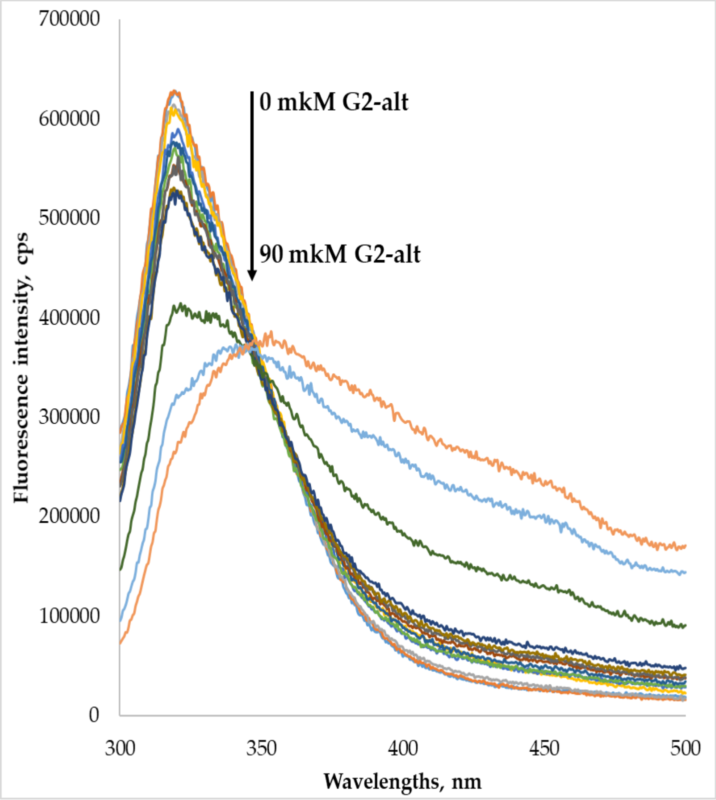

Figure S54. Fluorescence spectra of noradrenaline (100 µM) in presence of different concentrations of G2-alt (0-90 µM) in phosphate buffer, pH = 7.4, 50 mM.

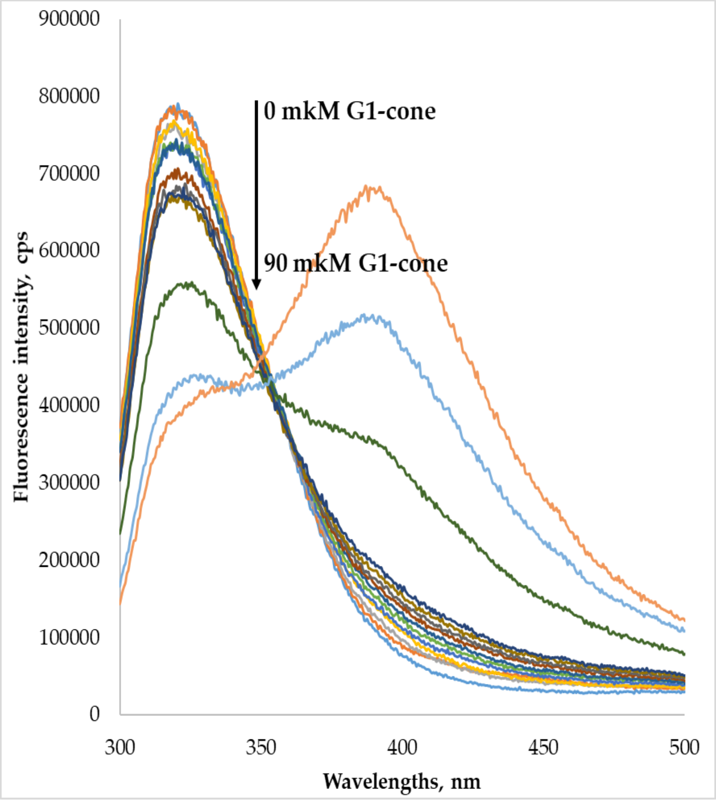

Figure S55. Fluorescence spectra of adrenaline (100 µM) in presence of different concentrations of G1-cone (0-90 µM) in phosphate buffer, pH = 7.4, 50 mM.

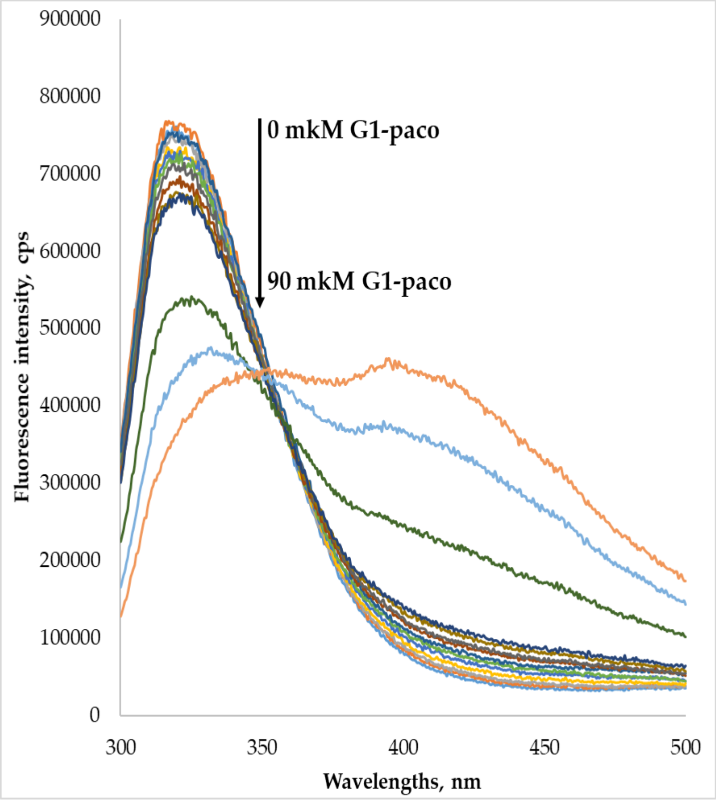

Figure S56. Fluorescence spectra of adrenaline (100 µM) in presence of different concentrations of G1-paco (0-90 µM) in phosphate buffer, pH = 7.4, 50 mM.

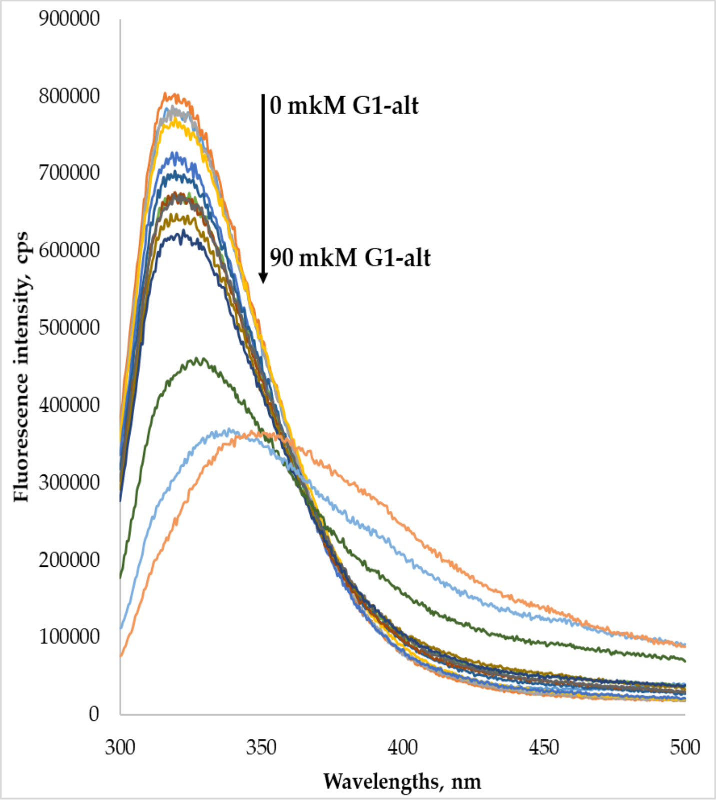

Figure S57. Fluorescence spectra of adrenaline (100 µM) in presence of different concentrations of G1-alt (0-90 µM) in phosphate buffer, pH = 7.4, 50 mM.

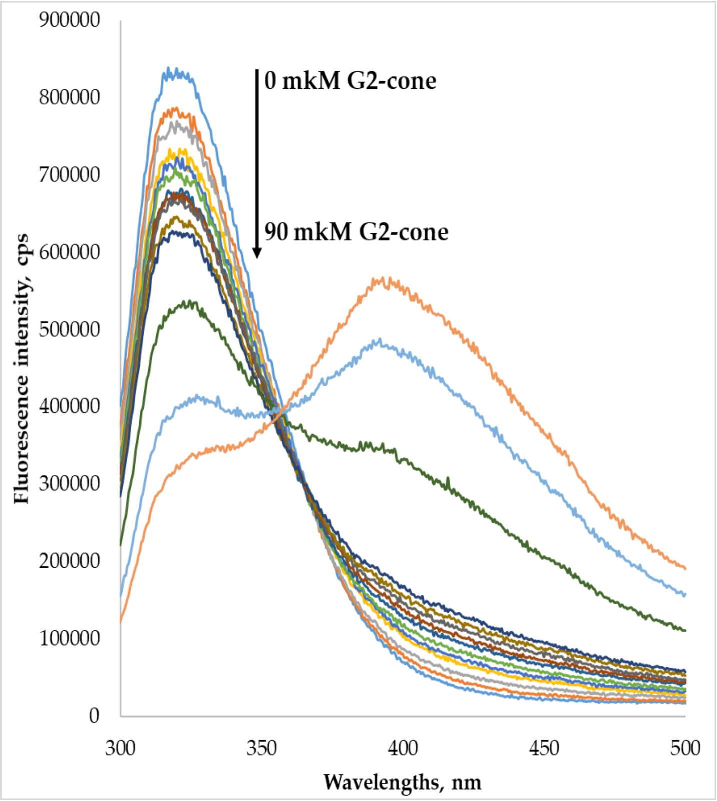

Figure S58. Fluorescence spectra of adrenaline (100 μM) in presence of different concentrations of G2-cone (0-90 μM) in phosphate buffer, pH = 7.4, 50 mM.

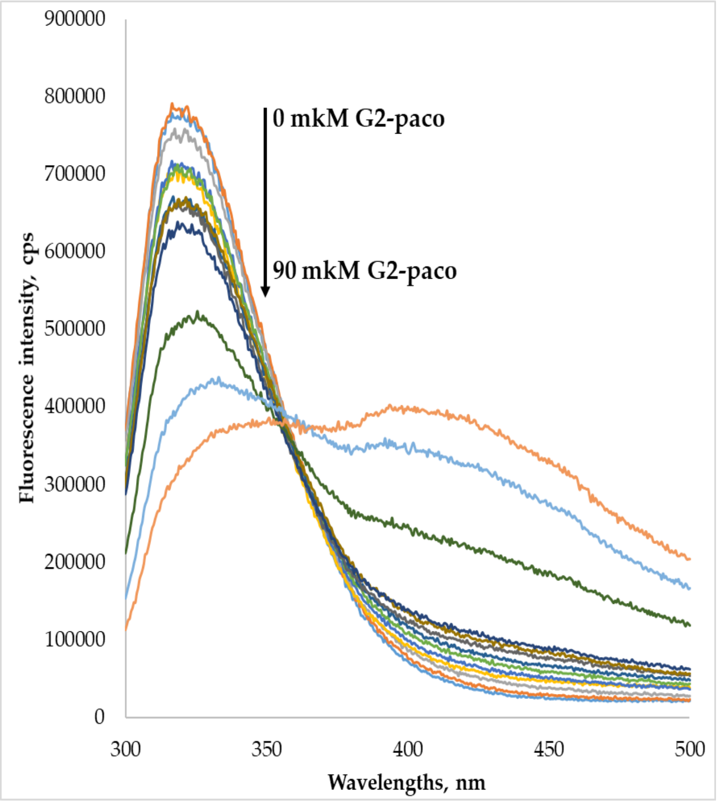

Figure S59. Fluorescence spectra of adrenaline (100 μM) in presence of different concentrations of G2-paco (0-90 μM) in phosphate buffer, pH = 7.4, 50 mM.

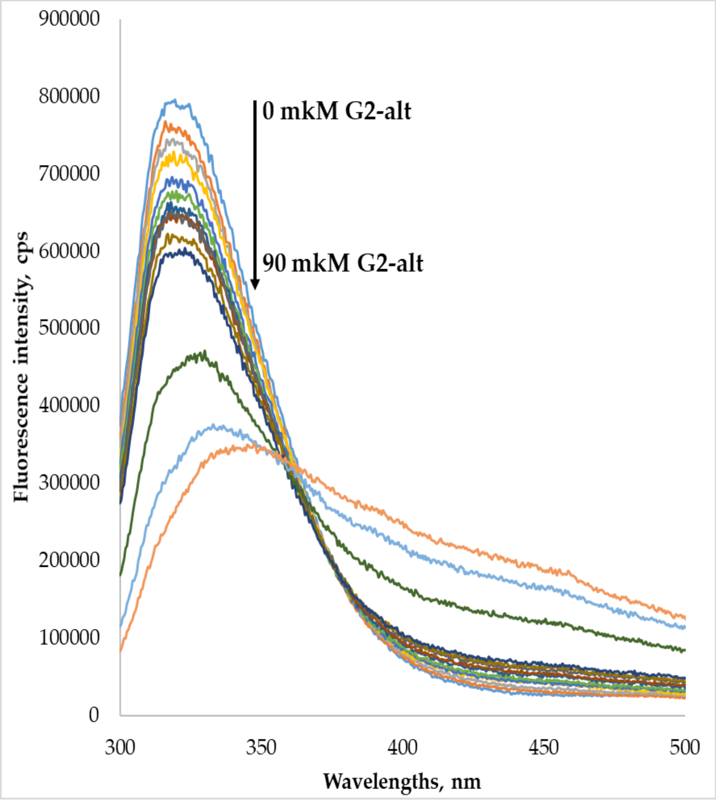

Figure S60. Fluorescence spectra of adrenaline (100 μM) in presence of different concentrations of G2-alt (0-90 μM) in phosphate buffer, pH = 7.4, 50 mM.

Table S1. Fit ID for the data obtained, calculated by Bindfit (ratio 1:1).

	**dopamine HCl**	***L*-adrenaline HCl**	***L*-noradrenaline HCl**
**G1-cone**	e181dcfe-22d1-4b7b-bc74-07080ba06526	db53bbeb-c076-4938-99c2-c010718187c2	c87eb897-55c7-4656-b7a7-ae97ea8f15b4
**G1-paco**	abc384f6-9649-4d80-9734-593b3d08f7b7	426769da-2047-4b2e-9ac4-4c291a62f829	4b6c98fa-f41f-4050-8f19-c81ef12a5b9a
**G1-alt**	087e0832-2262-470c-9829-90280deda834	08fe3254-3114-4fef-82c2-11d80749a00f	4fb3b28c-8d9c-47a5-bd08-e34b6784d144
**G2-cone**	83f889cc-59ad-4b2d-8946-f9d68616452d	823774b7-155b-41bd-953d-c72c0feb1bcd	eb60464e-aea9-468b-8496-539b9daeee5b
**G2-paco**	892f261c-5f8f-4a62-9ce7-5c87d622573e	7b2b4374-7313-4c01-b34d-cd8f1e1988bc	7dec3fce-4abb-424a-b6c7-88920e7ff889
**G2-alt**	9865f504-cc05-42ae-8a95-4291090fe5ee	5a64d07a-313f-464d-bd67-610a09c8c5c5	20e159ec-d7ce-4cf1-8410-4e67c94d533f

3.3. ^{1}H and 2D ^{1}H-^{1}H NOESY NMR spectra

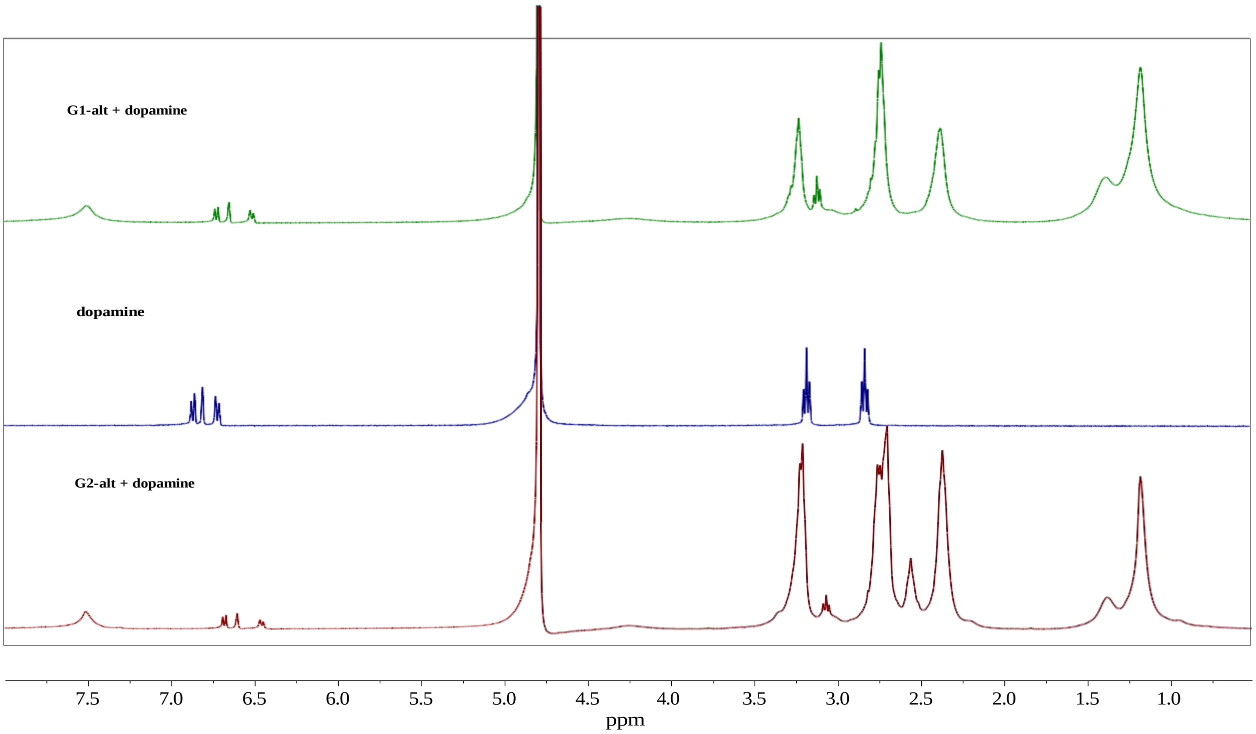

Figure S61. ^{1}H NMR spectra of pure dopamine, G1-alt/dopamine and G2-alt/dopamine mixtures (D_2O, 25°C, 400 MHz, concentration of the compounds 1×10^{-2} M, ratio 1:1).

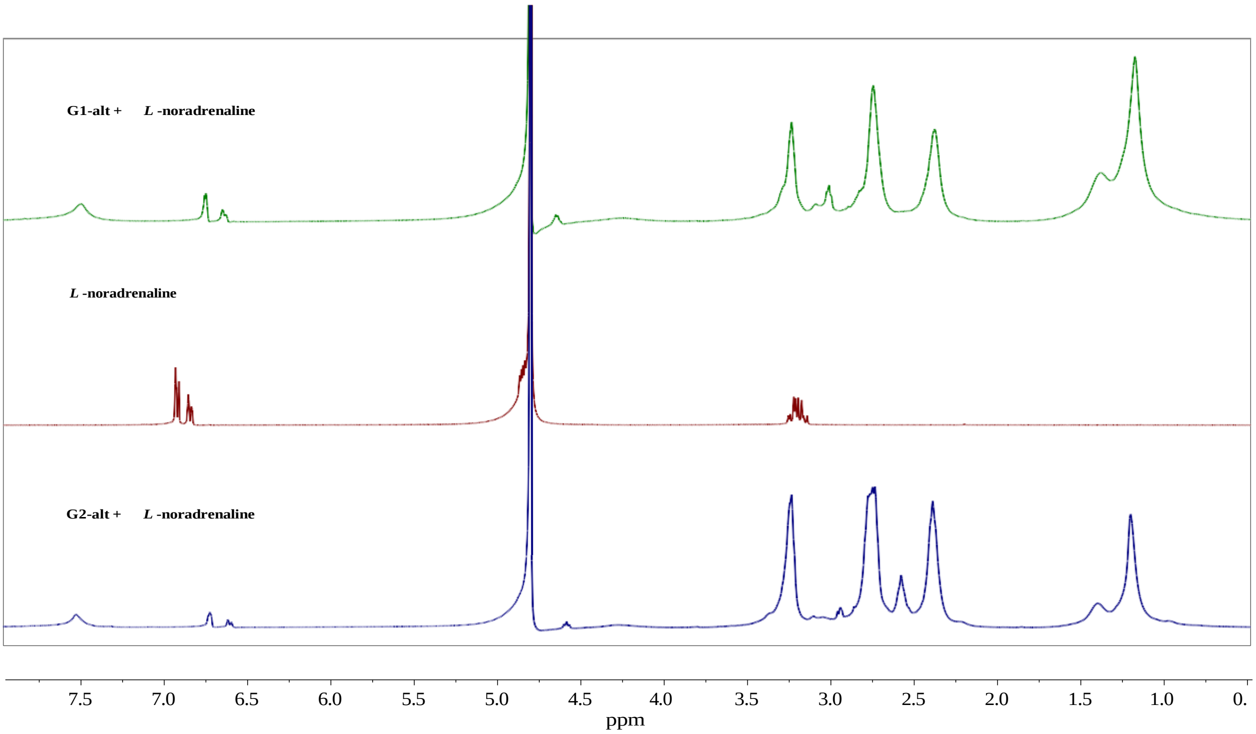

Figure S62. ^{1}H NMR spectra of pure noradrenaline, G1-alt/noradrenaline and G2-alt/noradrenaline mixtures (D_2O, 25°C, 400 MHz, concentration of the compounds 1×10^{-2} M, ratio 1:1).

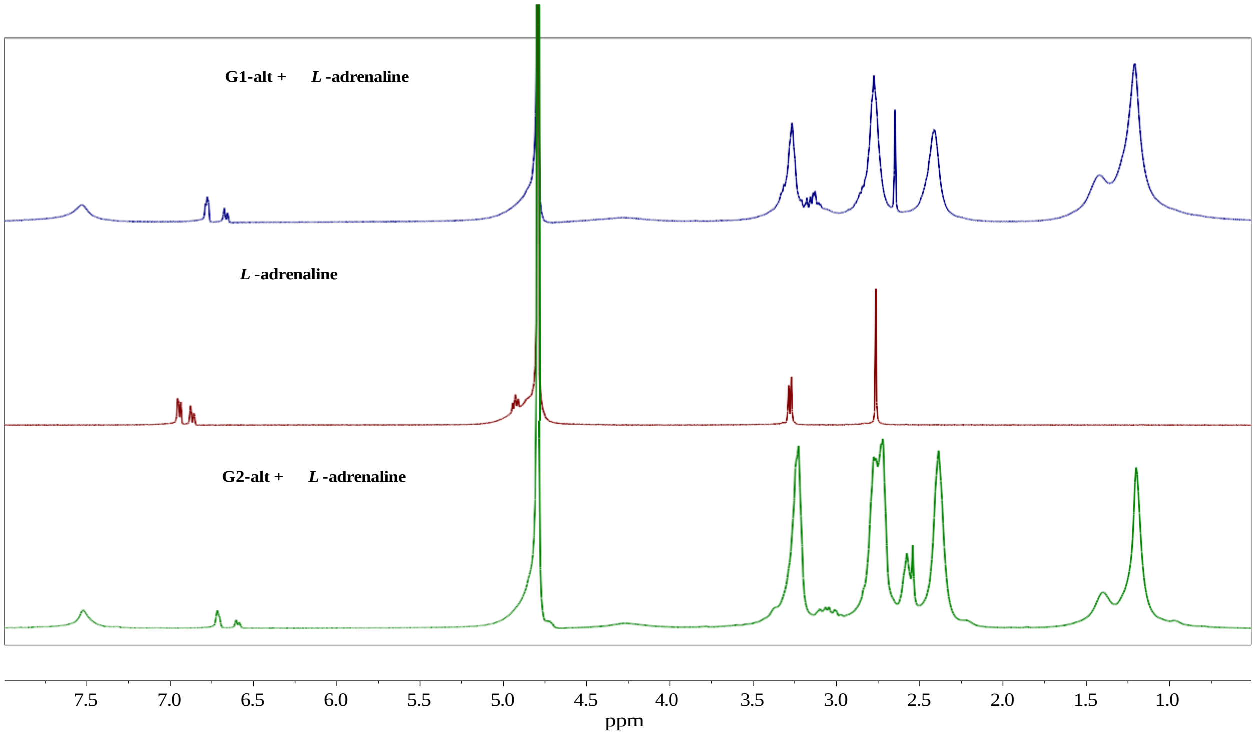

Figure S63. ^{1}H NMR spectra of pure adrenaline, G1-alt/adrenaline and G2-alt/adrenaline mixtures (D_2O, 25°C, 400 MHz, concentration of the compounds 1×10^{-2} M, ratio 1:1).

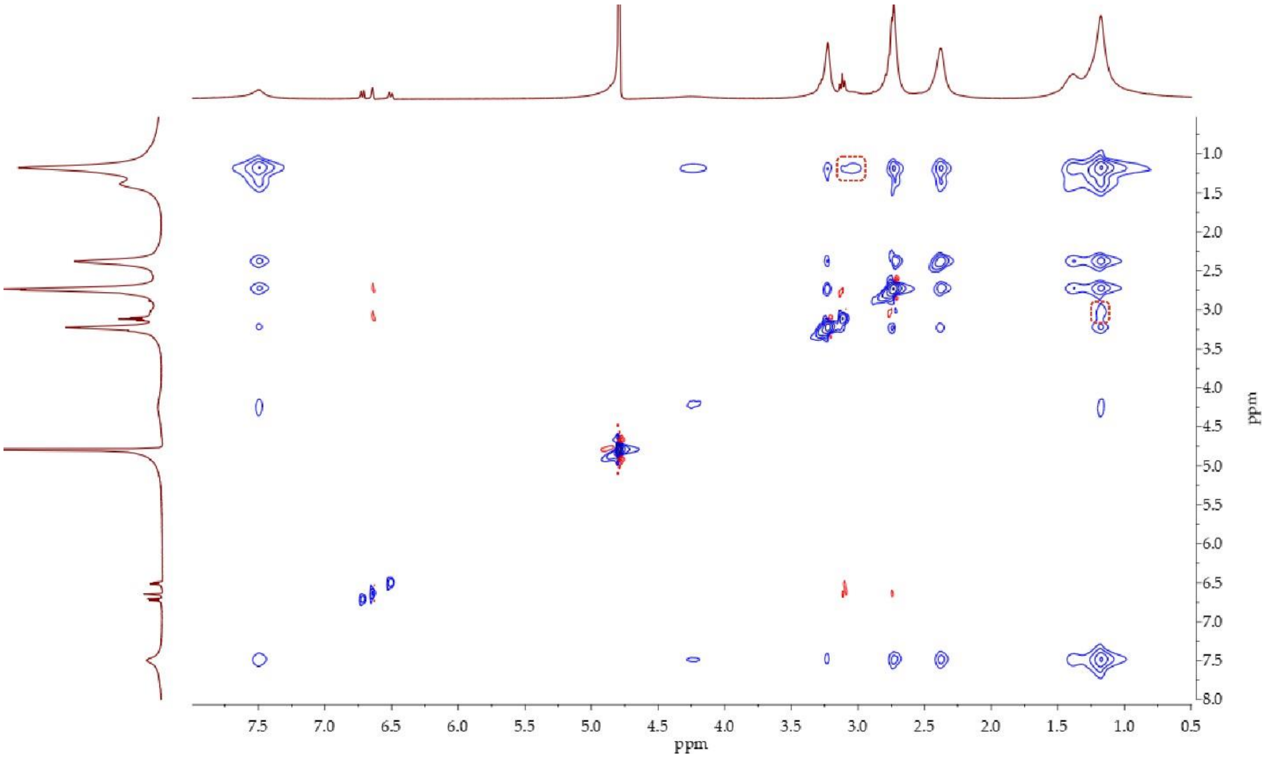

Figure S64. 2D ^{1}H–^{1}H NOESY NMR spectra (D_2O, 25°C, 400 MHz, the compounds concentration 1×10^{-2} M, ratio 1:1) of **G1-alt**/dopamine mixture.

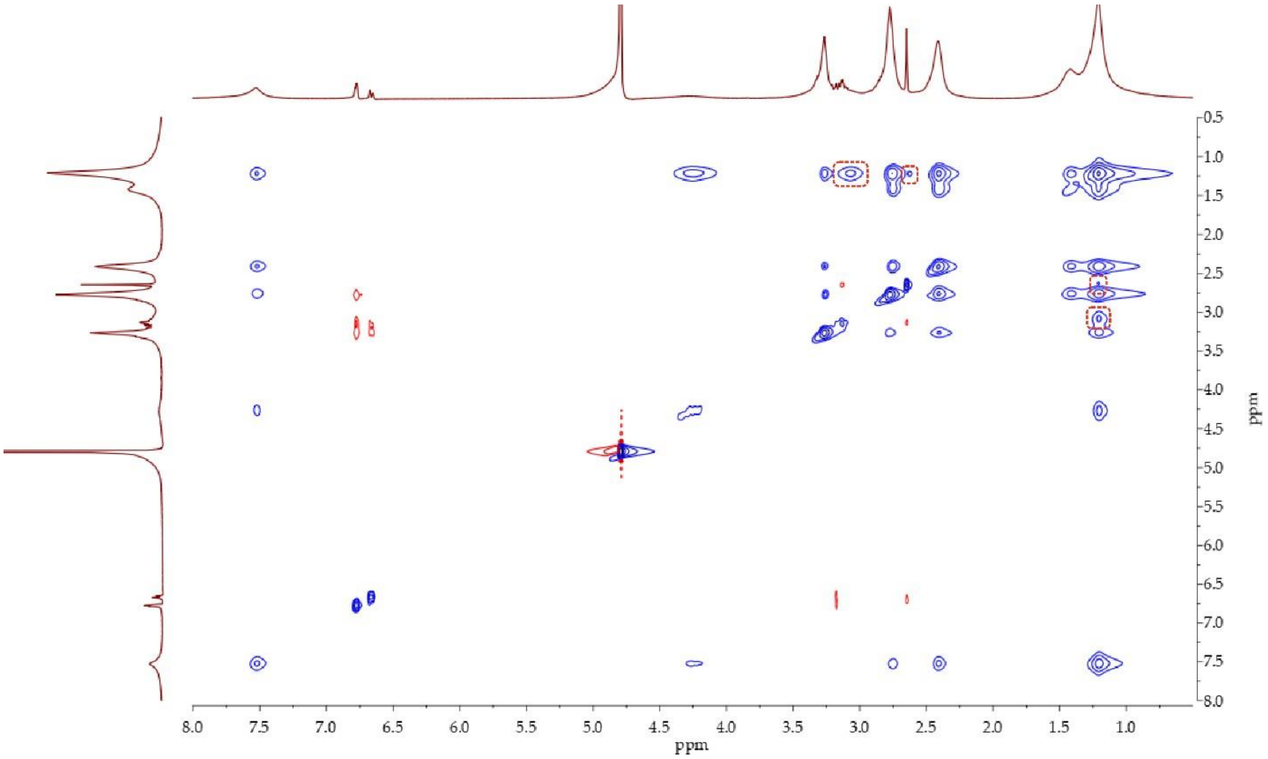

Figure S65. 2D ^1H–^1H NOESY NMR spectra (D_2O, 25°C, 400 MHz, the compounds concentration 1×10^{-2} M, ratio 1:1) of **G1-alt**/adrenaline mixture.

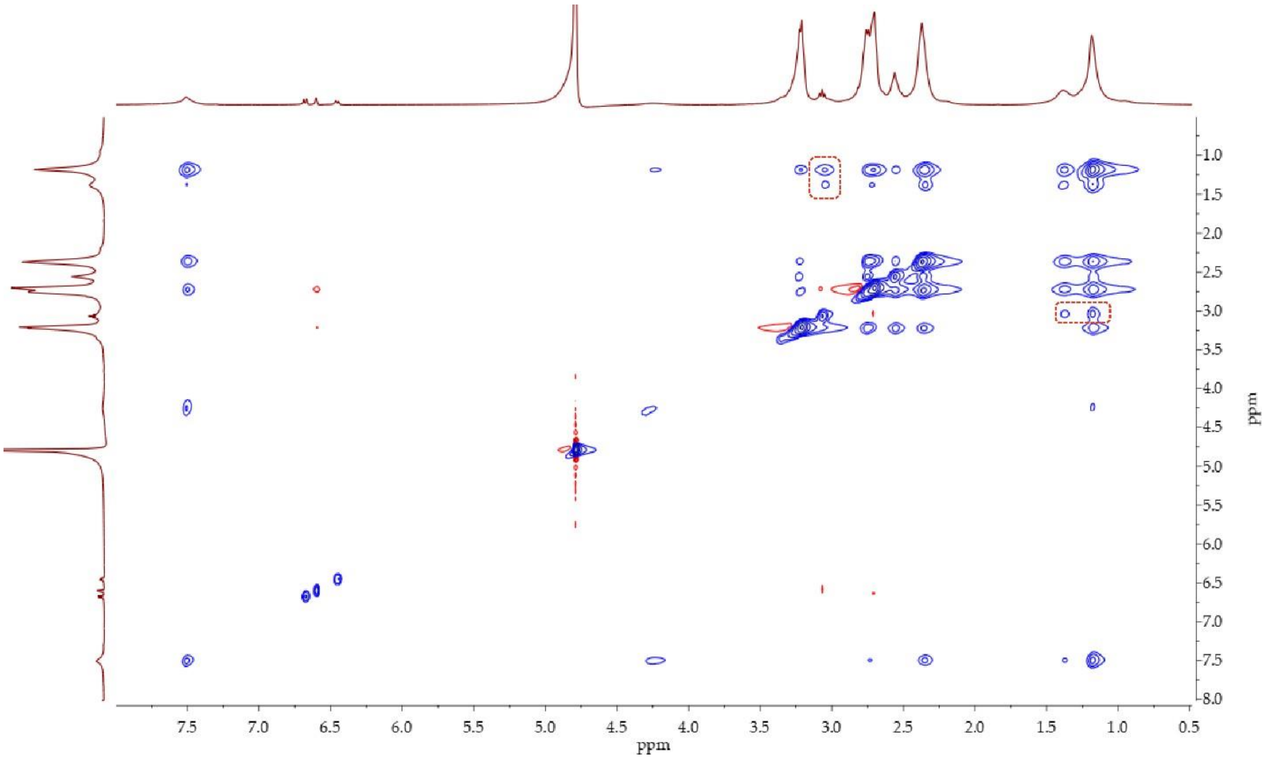

Figure S66. 2D ^1H–^1H NOESY NMR spectra (D_2O, 25°C, 400 MHz, the compounds concentration 1×10^{-2} M, ratio 1:1) of **G2-alt**/dopamine mixture.

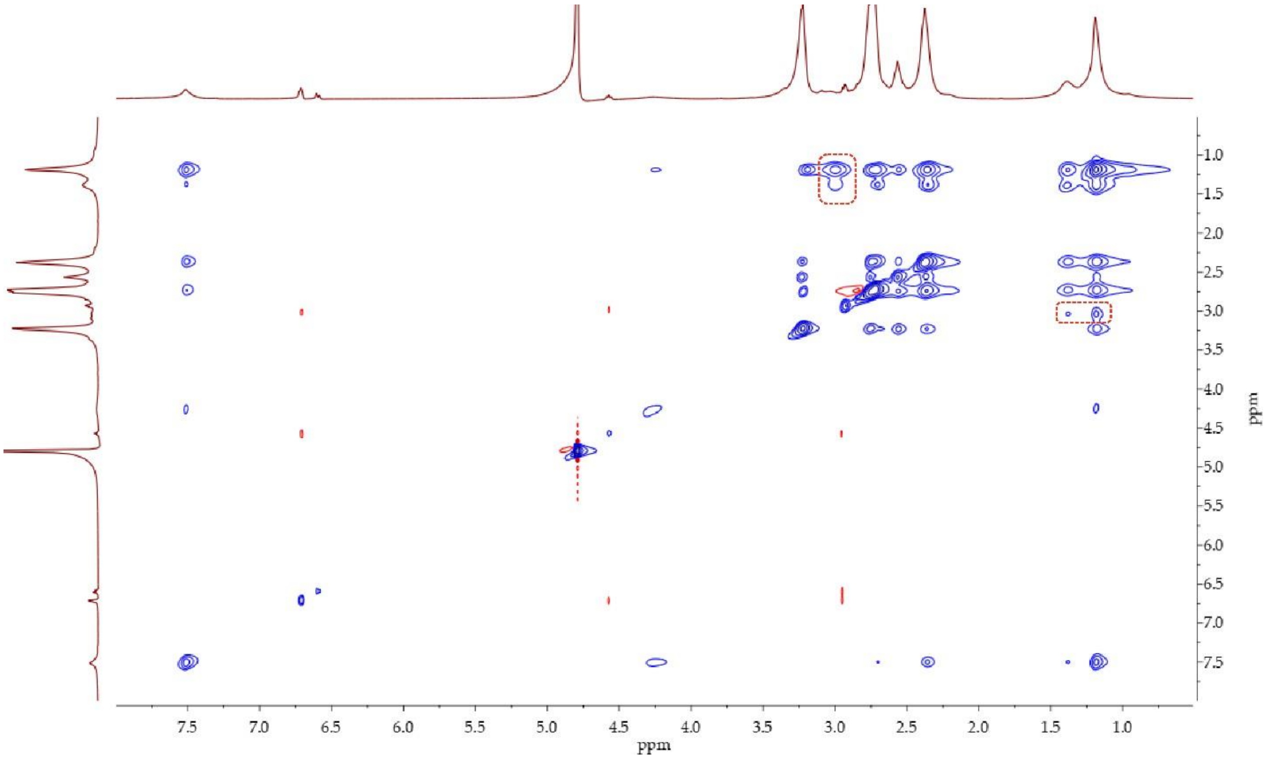

Figure S67. 2D ^{1}H–^{1}H NOESY NMR spectra (D_2O, 25°C, 400 MHz, the compounds concentration 1×10^{-2} M, ratio 1:1) of **G1-alt**/noradrenaline mixture.

3.4. DLS data

The particle size was determined by the Zetasizer Nano ZS instrument (Malvern Instruments) at 293 K. The instrument contains the 4 mW He-Ne laser operating at the wavelength of 633 nm and incorporates noninvasive backscatter optics (NIBS). The measurements were performed at the detection angle of 173°, and the measurement position within the cuvette was automatically determined by the software. The results were processed with the DTS (Dispersion Technology Software 4.20) software package. The experiments were carried out in polystyrene cuvettes in 50 mM phosphate buffer (pH = 7.4). Concentrations of the dendrimers in individual solutions ranged from 1×10^{-5} to 1×10^{-4} M. Concentrations of the catecholamines and of the **G1** and **G2** dendrimers in the mixtures were 1×10^{-5} M. The determination of the particle size was carried out in 1 h after the sample preparation.

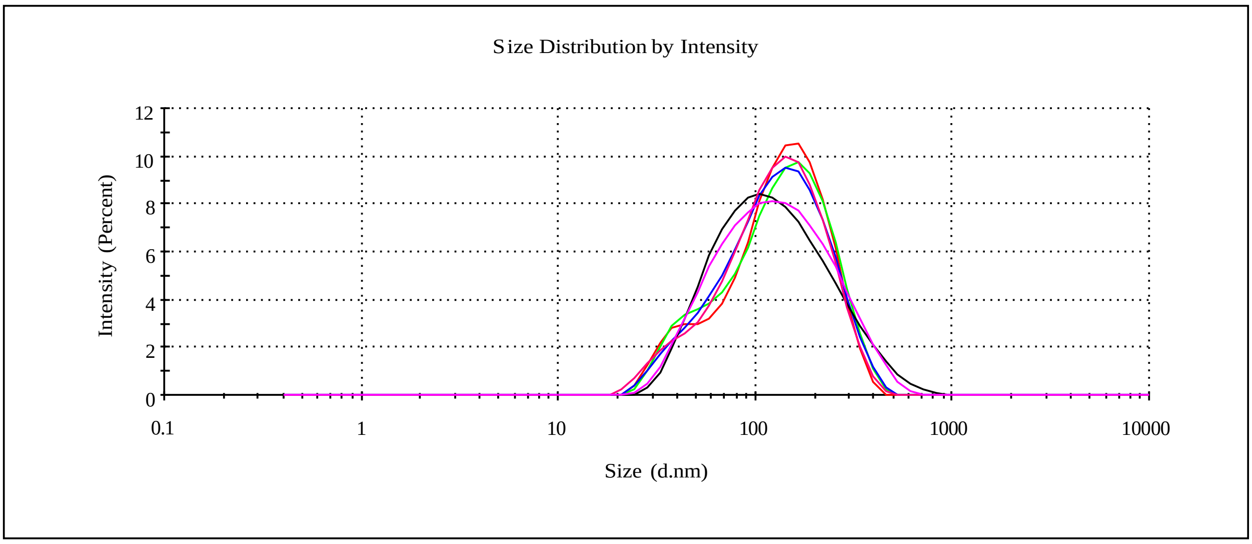

Figure S68. The size distribution (intensity) of **G1-monomer** (2×10^{-4} M) in phosphate buffer (pH = 7.4, 50 mM).

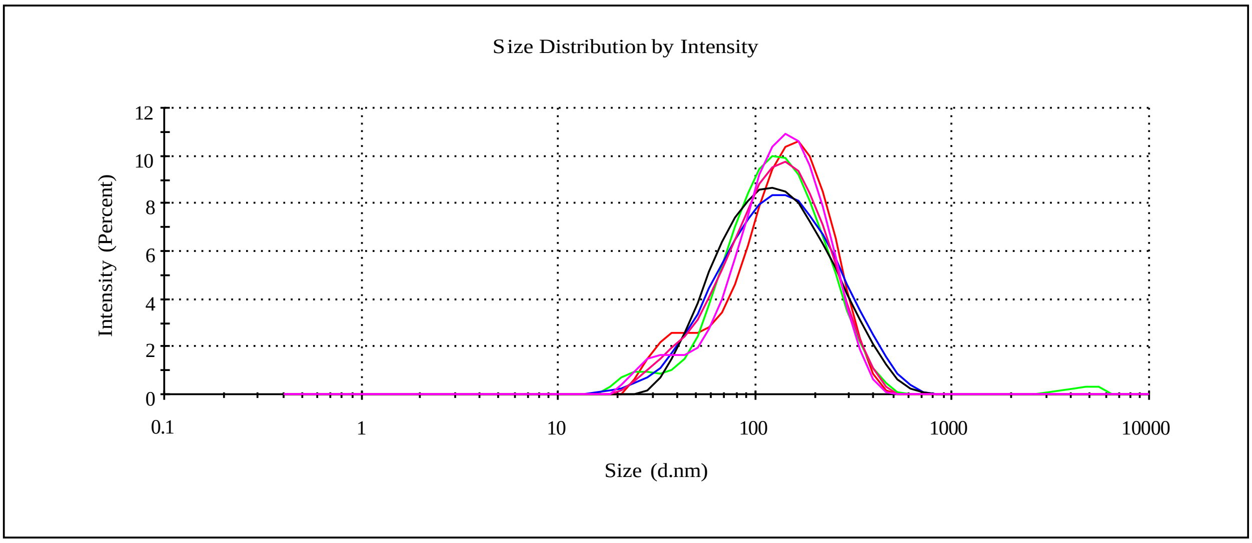

Figure S69. The size distribution (intensity) of **G1-monomer** (1×10^{-4} M) in phosphate buffer (pH = 7.4, 50 mM).

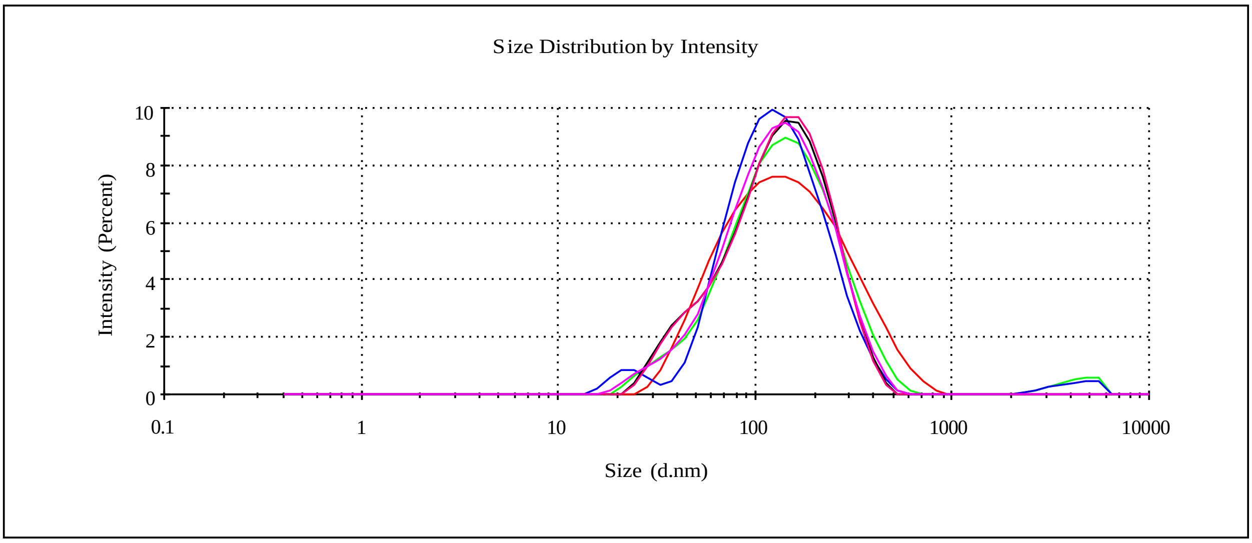

Figure S70. The size distribution (intensity) of **G1-monomer** (5×10^{-5} M) in phosphate buffer (pH = 7.4, 50 mM).

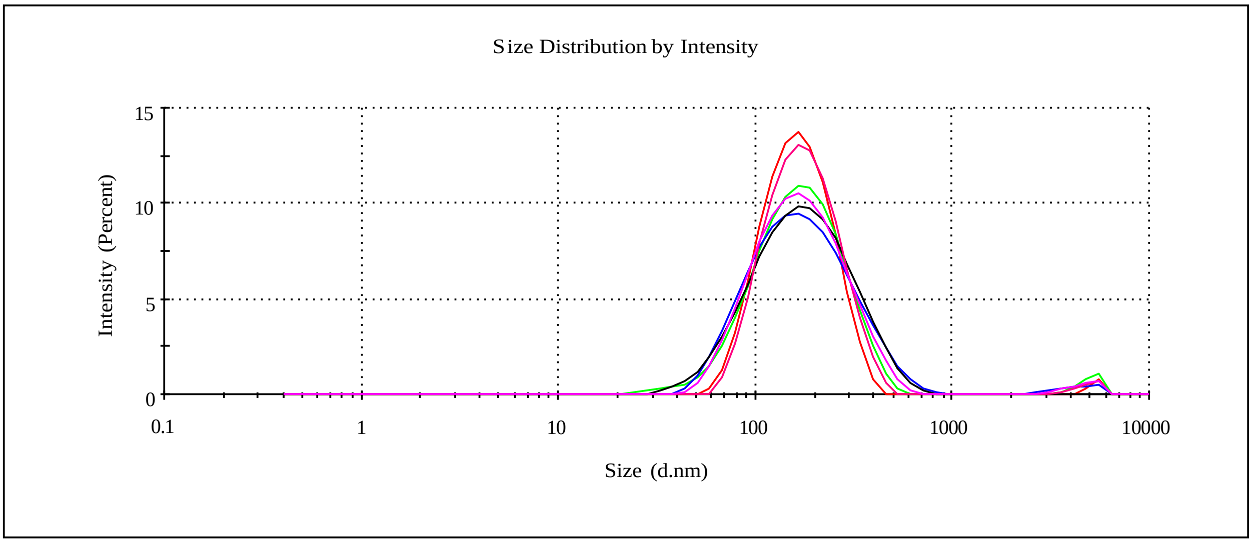

Figure S71. The size distribution (intensity) of **G1-monomer** (1×10^{-5} M) in phosphate buffer (pH = 7.4, 50 mM).

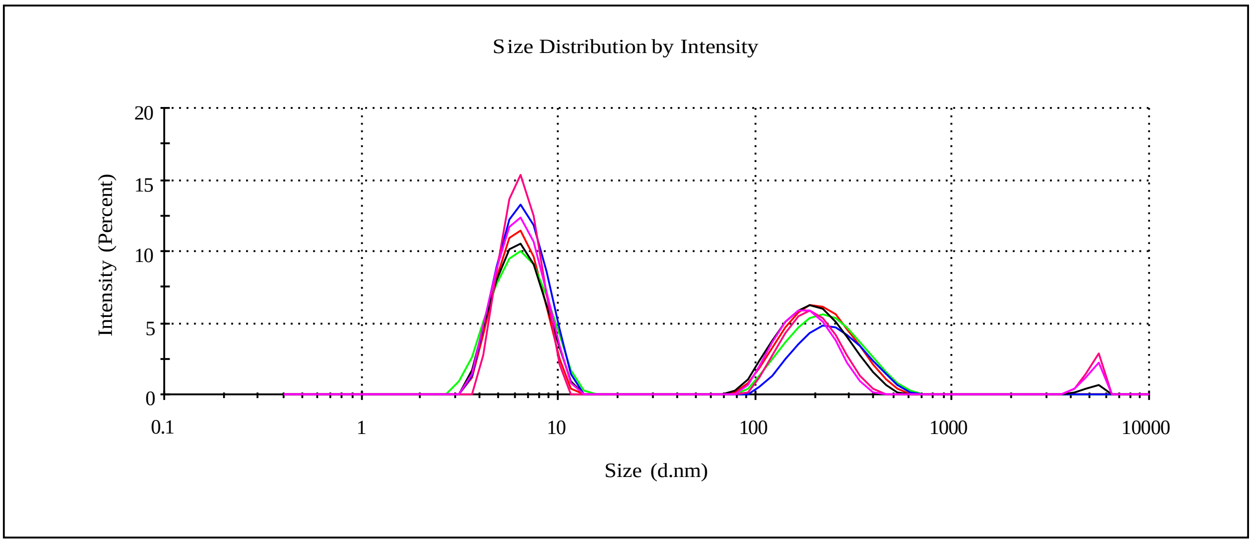

Figure S72. The size distribution (intensity) of **G1-cone** (1×10^{-4} M) in phosphate buffer (pH = 7.4, 50 mM).

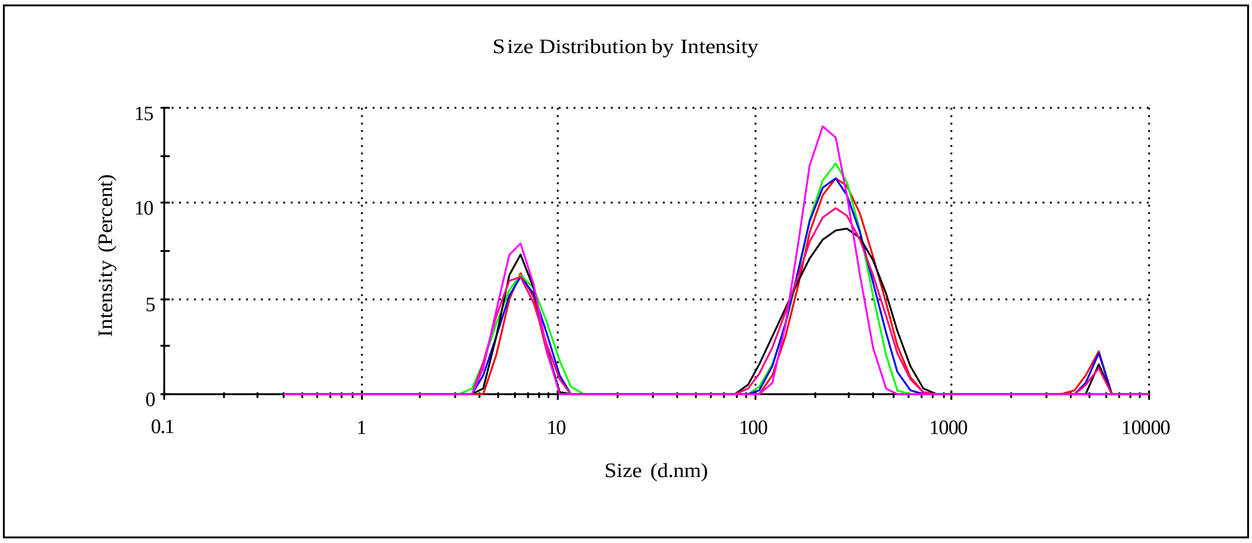

Figure S73. The size distribution (intensity) of **G1-cone** (5×10^{-5} M) in phosphate buffer (pH = 7.4, 50 mM).

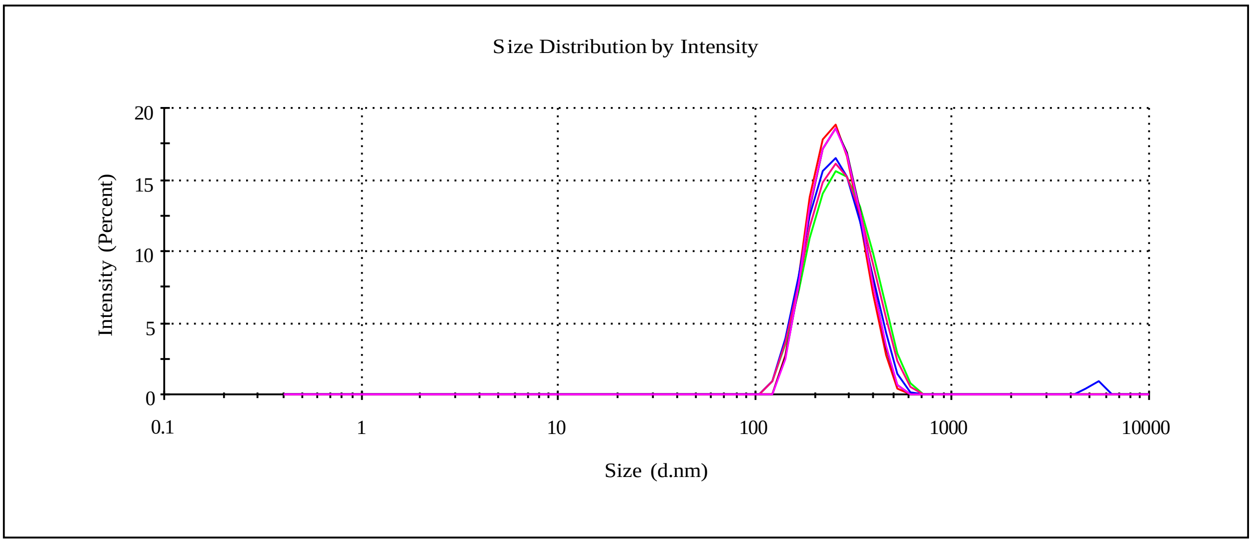

Figure S74. The size distribution (intensity) of **G1-cone** (1×10^{-5} M) in phosphate buffer (pH = 7.4, 50 mM).

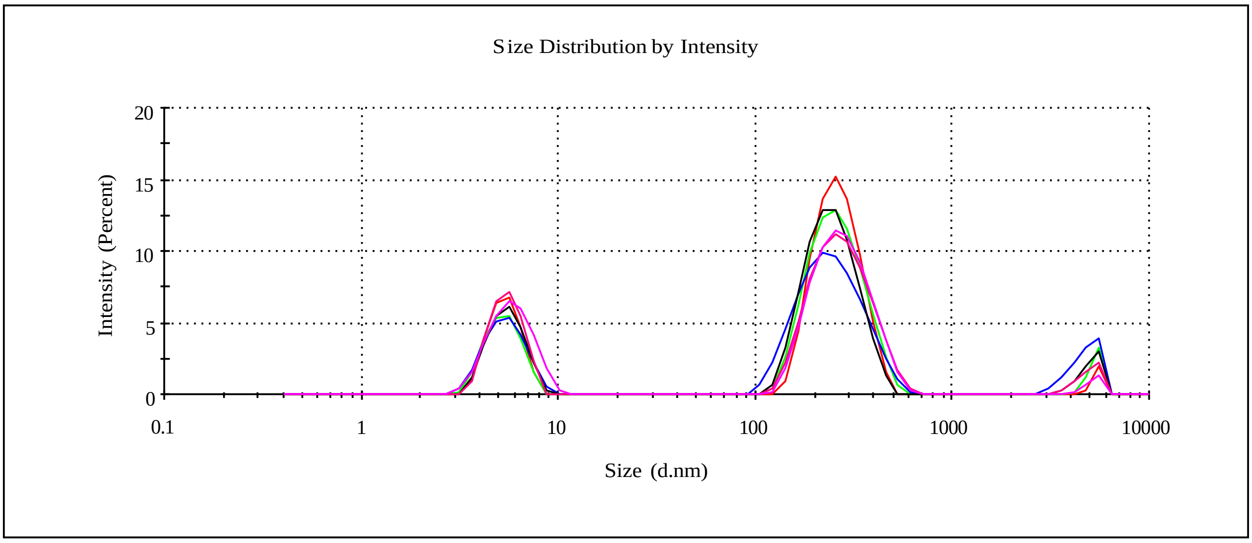

Figure S75. The size distribution (intensity) of **G1-paco** (1×10^{-4} M) in phosphate buffer (pH = 7.4, 50 mM).

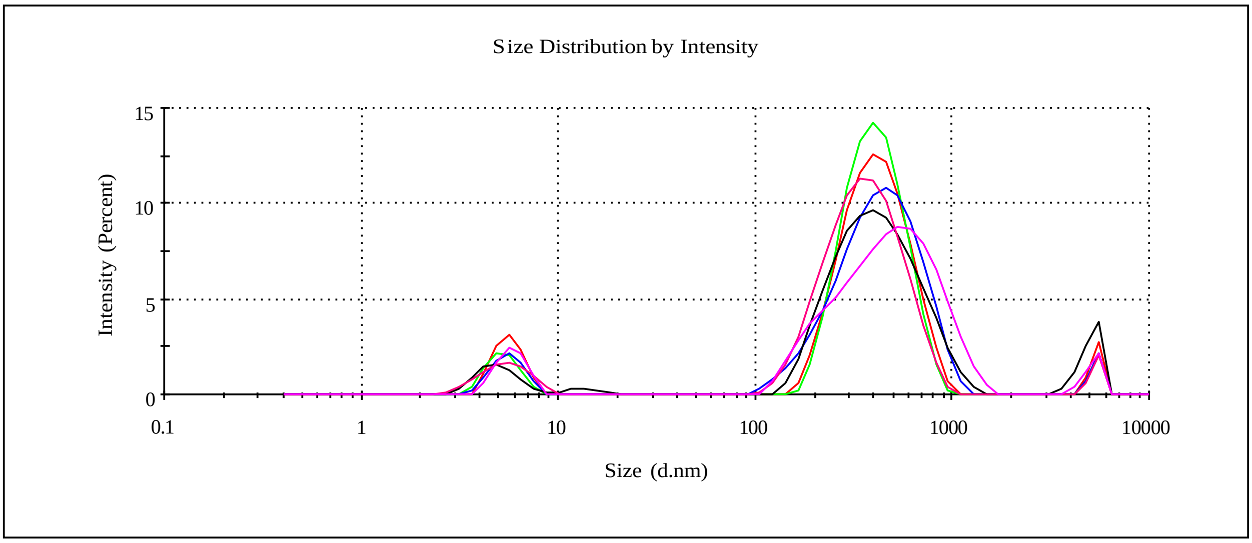

Figure S76. The size distribution (intensity) of **G1-paco** (5×10^{-5} M) in phosphate buffer (pH = 7.4, 50 mM).

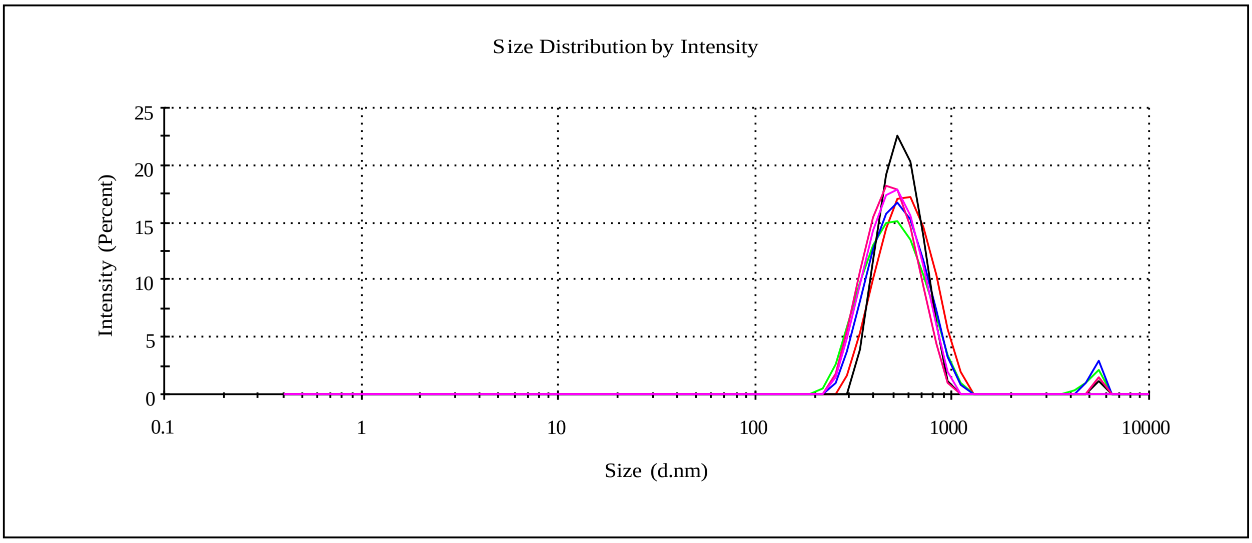

Figure S77. The size distribution (intensity) of **G1-paco** (1×10^{-5} M) in phosphate buffer (pH = 7.4, 50 mM).

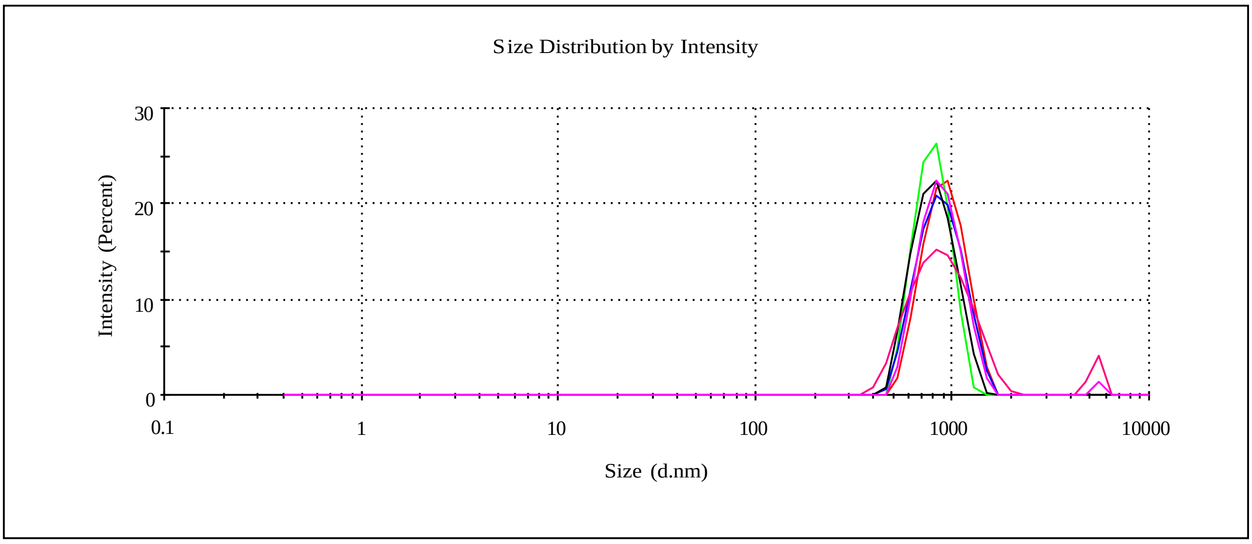

Figure S78. The size distribution (intensity) of **G1-alt** (1×10^{-4} M) in phosphate buffer (pH = 7.4, 50 mM).

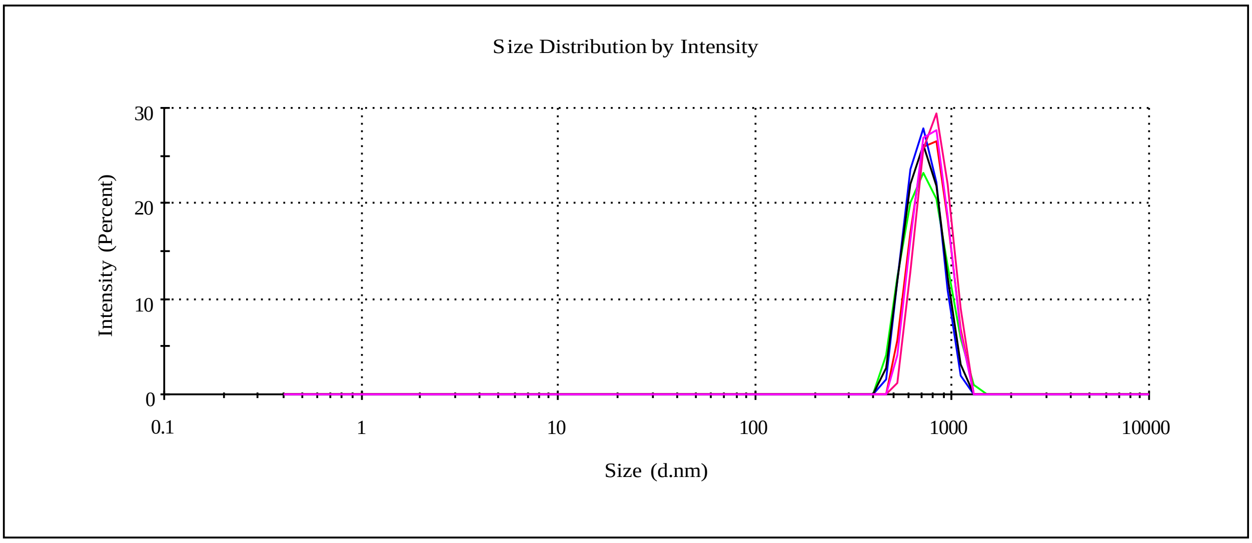

Figure S79. The size distribution (intensity) of **G1-alt** (5×10^{-5} M) in phosphate buffer (pH = 7.4, 50 mM).

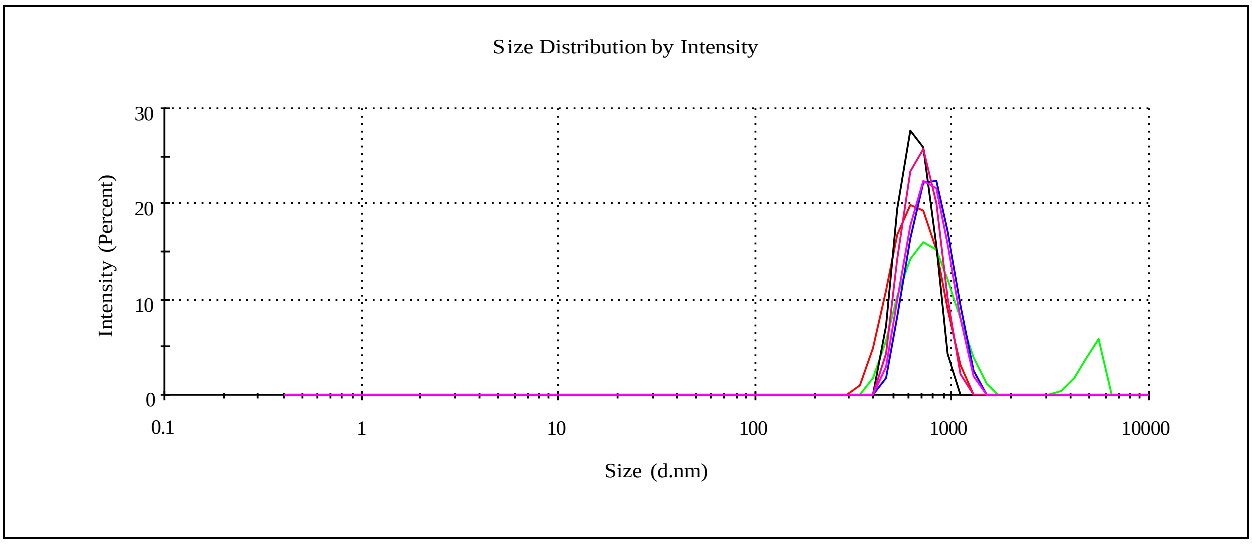

Figure S80. The size distribution (intensity) of **G1-alt** (1×10^{-5} M) in phosphate buffer (pH = 7.4, 50 mM).

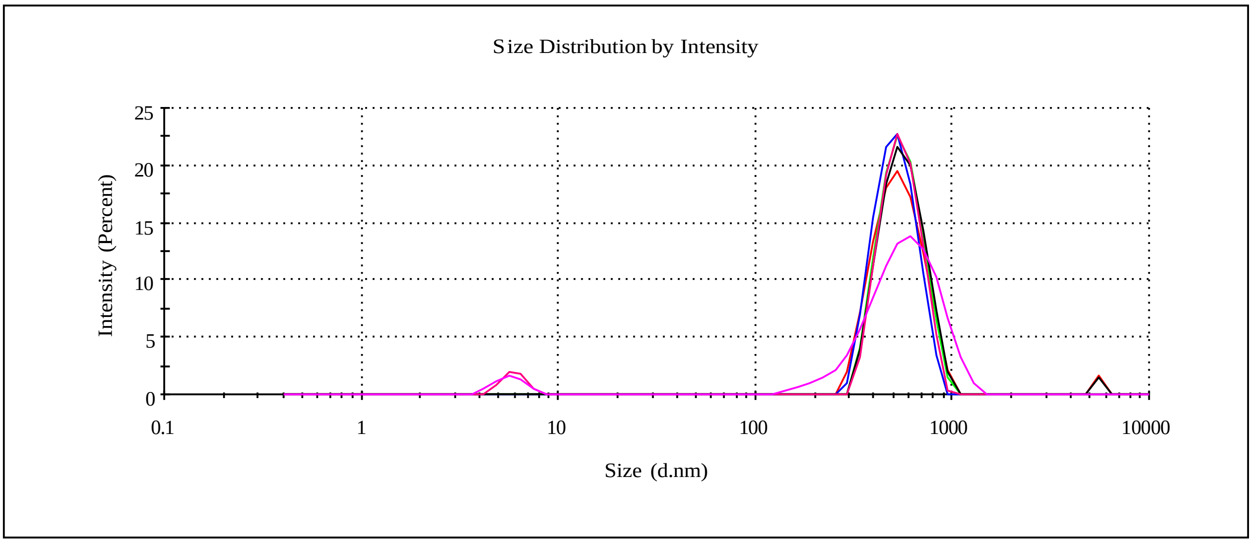

Figure S81. The size distribution (intensity) of **G2-cone** (1×10^{-4} M) in phosphate buffer (pH = 7.4, 50 mM).

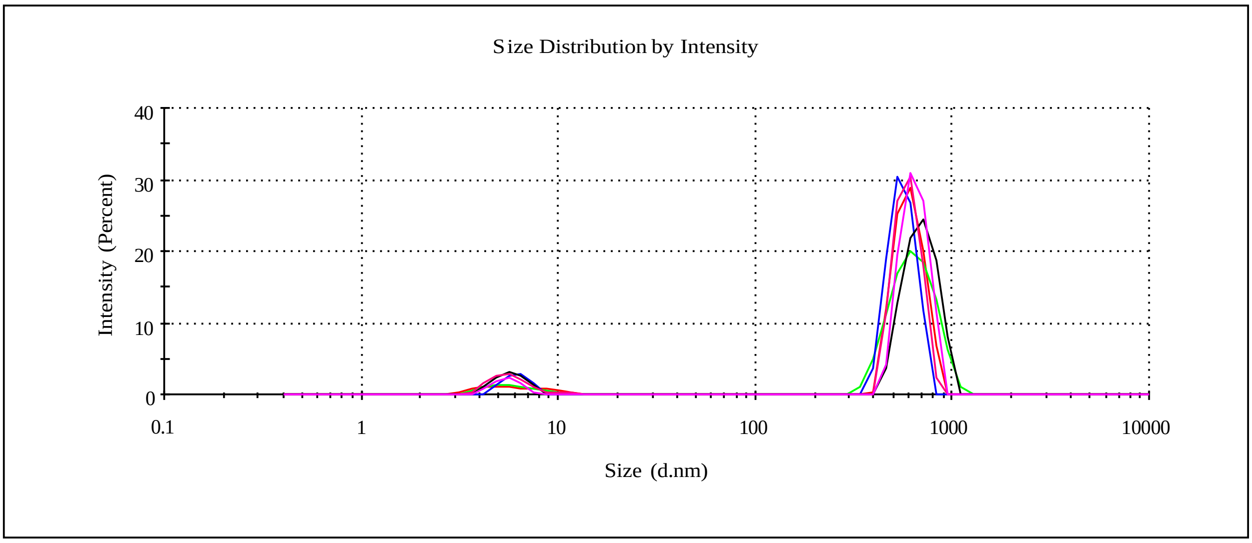

Figure S82. The size distribution (intensity) of **G2-cone** (5×10^{-5} M) in phosphate buffer (pH = 7.4, 50 mM).

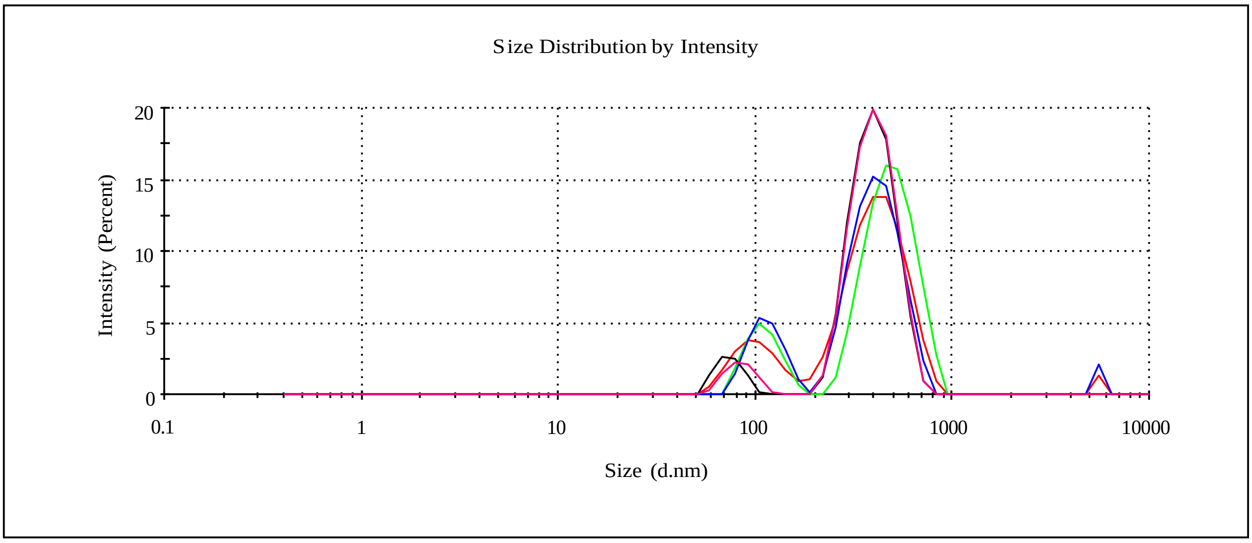

Figure S83. The size distribution (intensity) of **G2-cone** (1×10^{-5} M) in phosphate buffer (pH = 7.4, 50 mM).

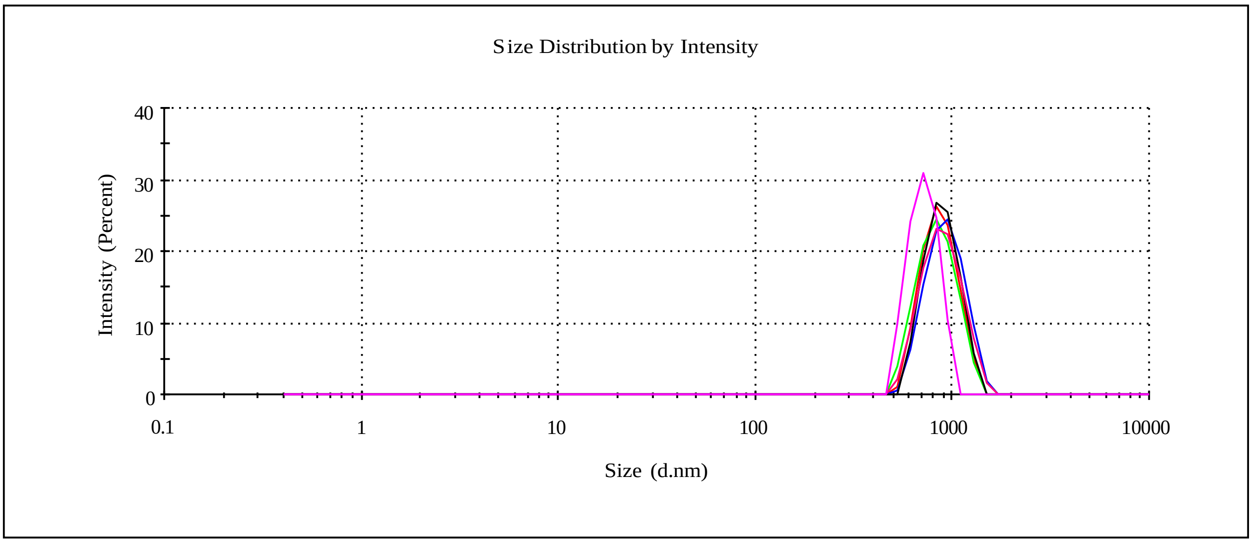

Figure S84. The size distribution (intensity) of **G2-paco** (1×10^{-4} M) in phosphate buffer (pH = 7.4, 50 mM).

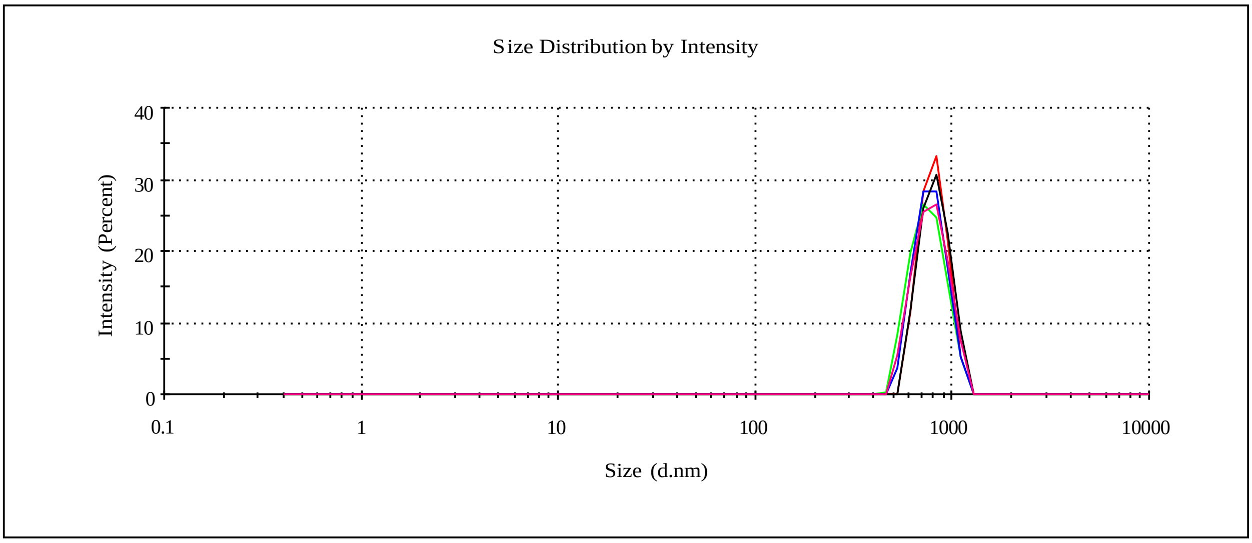

Figure S85. The size distribution (intensity) of **G2-paco** (5×10^{-5} M) in phosphate buffer (pH = 7.4, 50 mM).

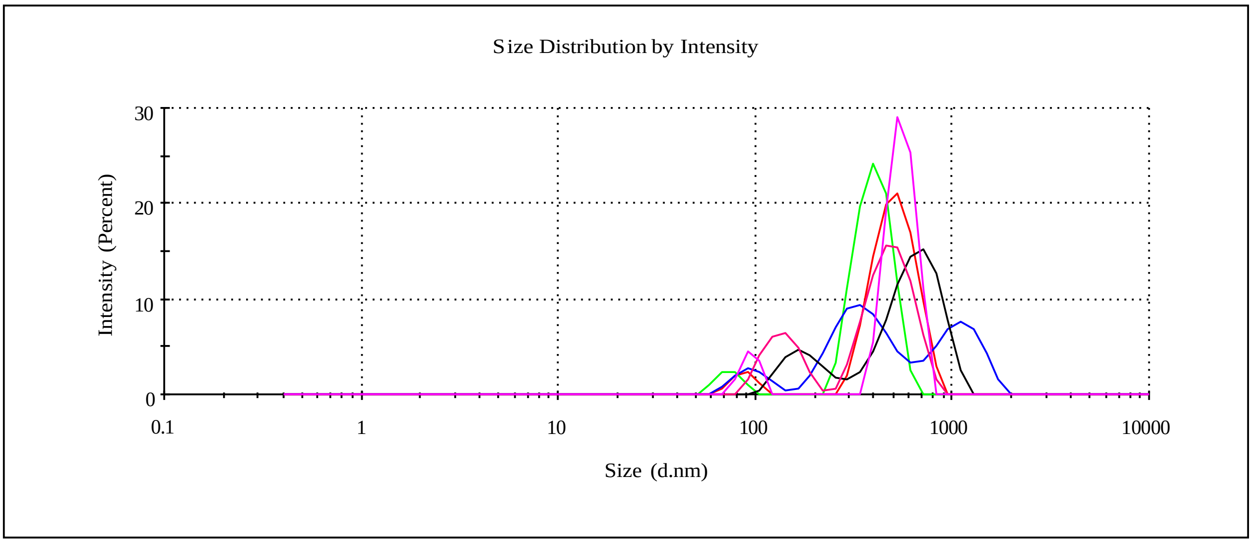

Figure S86. The size distribution (intensity) of **G2-paco** (1×10^{-5} M) in phosphate buffer (pH = 7.4, 50 mM).

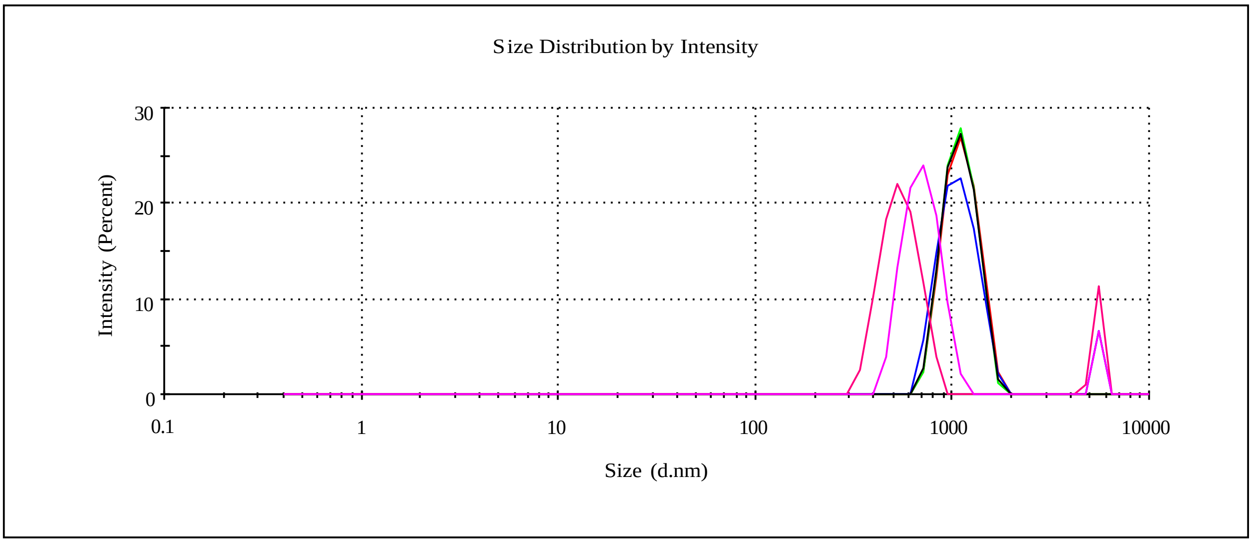

Figure S87. The size distribution (intensity) of **G2-alt** (1×10^{-4} M) in phosphate buffer (pH = 7.4, 50 mM).

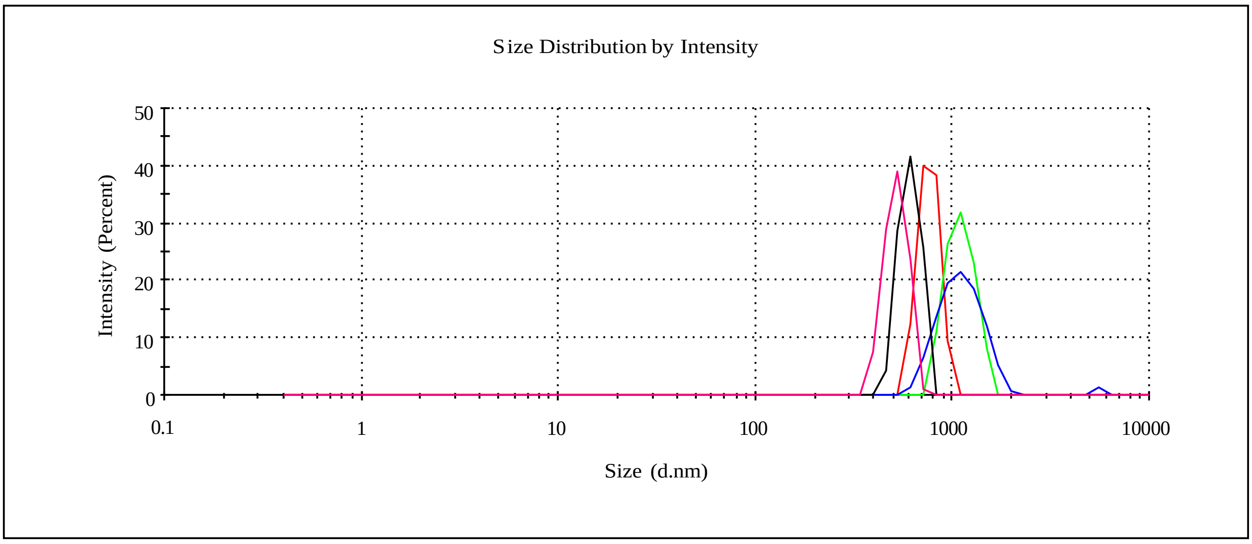

Figure S88. The size distribution (intensity) of **G2-alt** (5×10^{-5} M) in phosphate buffer (pH = 7.4, 50 mM).

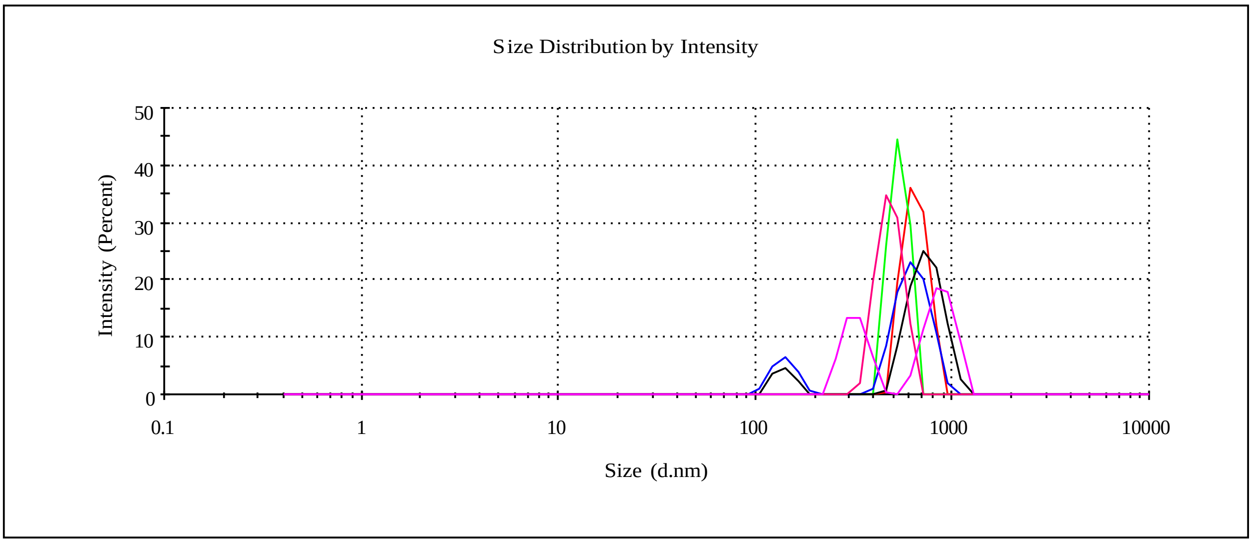

Figure S89. The size distribution (intensity) of **G2-alt** (1×10^{-5} M) in phosphate buffer (pH = 7.4, 50 mM).

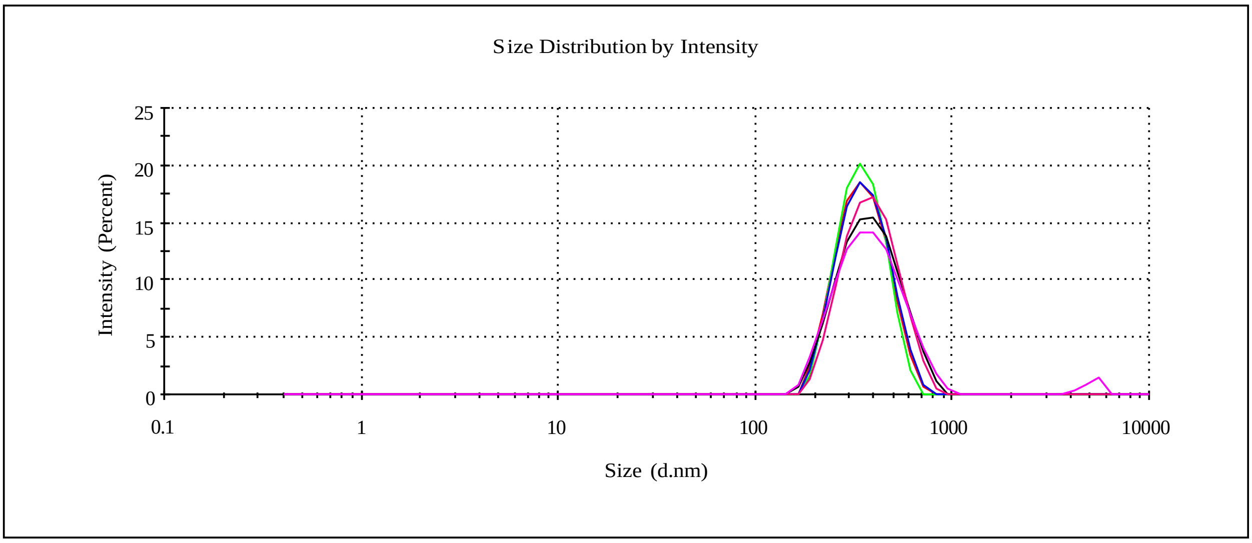

Figure S90. The size distribution (intensity) of systems **G1-cone**/dopamine (ratio 1:1, concentration of compounds 10^{-5} M) in phosphate buffer (pH = 7.4, 50 mM).

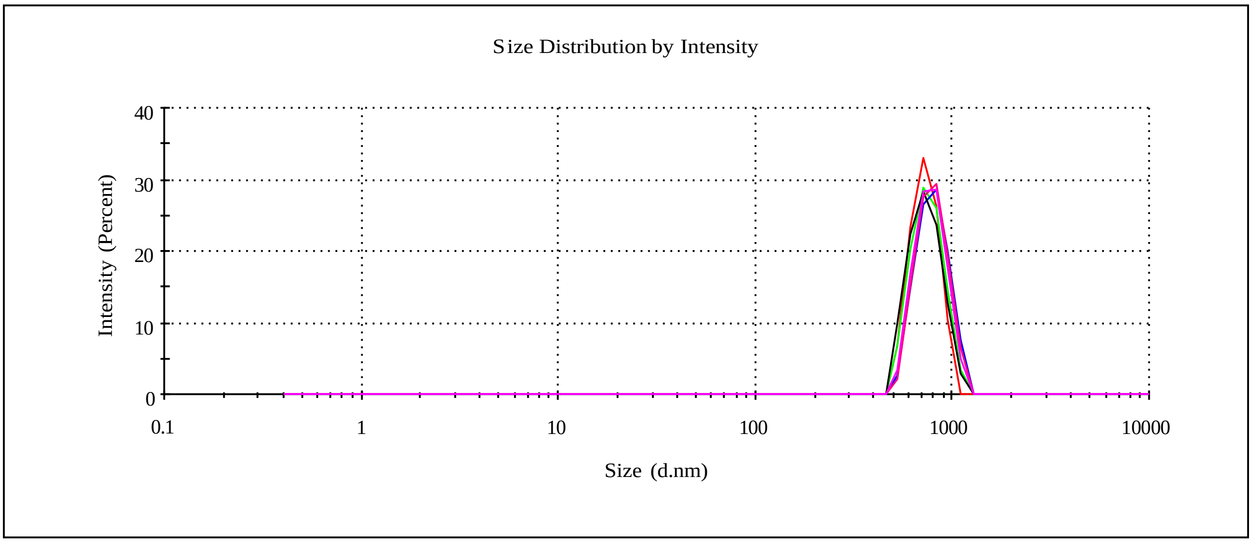

Figure S91. The size distribution (intensity) of systems **G1-paco**/dopamine (ratio 1:1, concentration of compounds 10^{-5} M) in phosphate buffer (pH = 7.4, 50 mM).

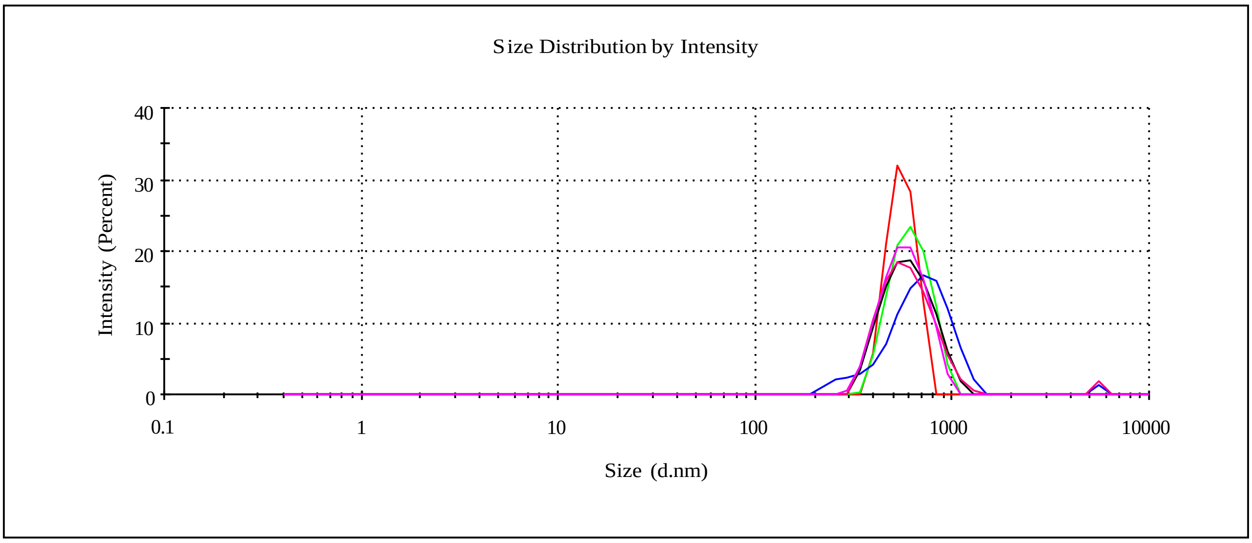

Figure S92. The size distribution (intensity) of systems **G1-alt**/dopamine (ratio 1:1, concentration of compounds 10^{-5} M) in phosphate buffer (pH = 7.4, 50 mM).

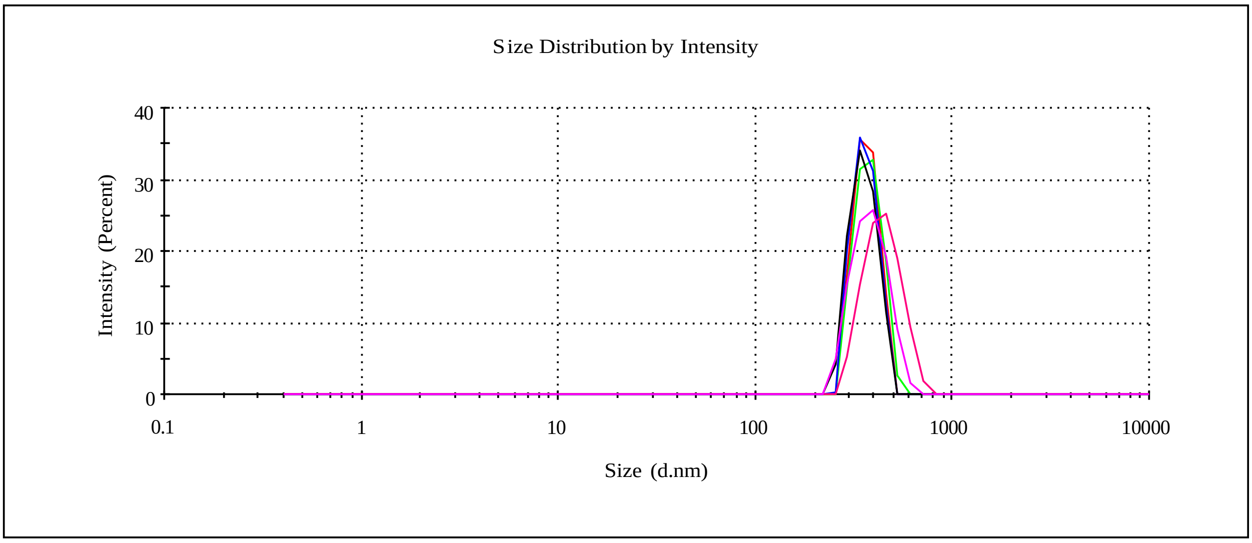

Figure S93. The size distribution (intensity) of systems **G2-cone**/dopamine (ratio 1:1, concentration of compounds 10^{-5} M) in phosphate buffer (pH = 7.4, 50 mM).

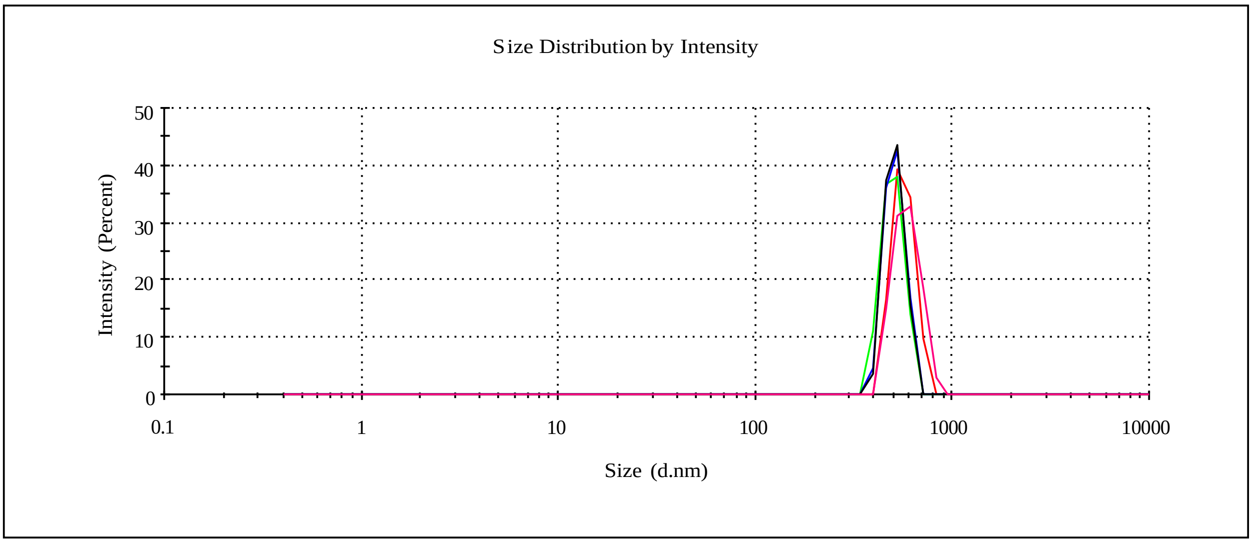

Figure S94. The size distribution (intensity) of systems **G2-paco**/dopamine (ratio 1:1, concentration of compounds 10^{-5} M) in phosphate buffer (pH = 7.4, 50 mM).

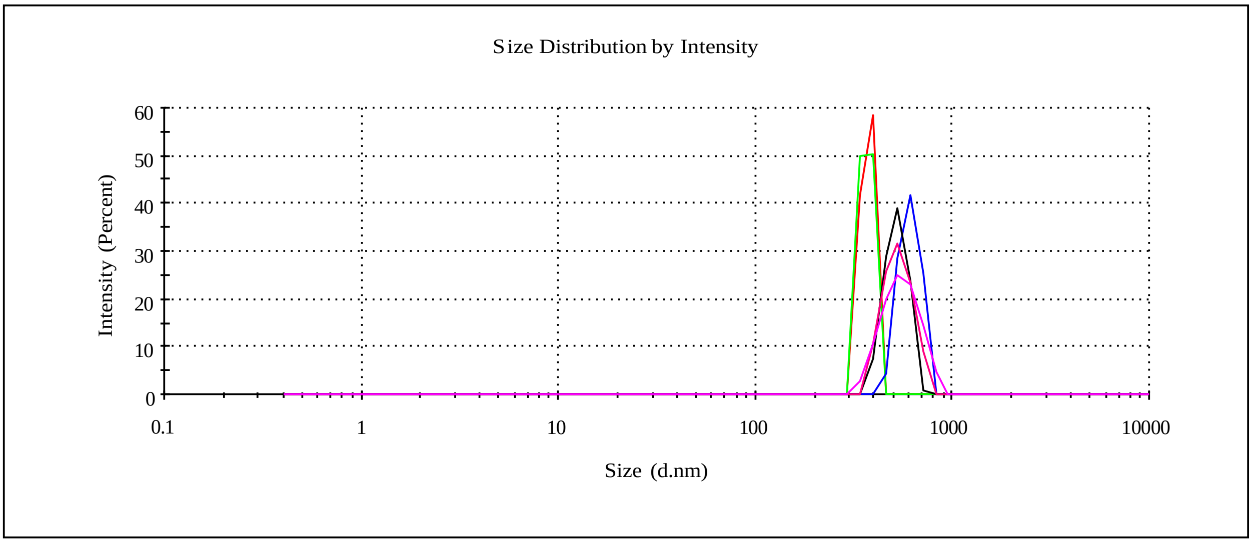

Figure S95. The size distribution (intensity) of systems **G2-alt**/dopamine (ratio 1:1, concentration of compounds 10^{-5} M) in phosphate buffer (pH = 7.4, 50 mM).

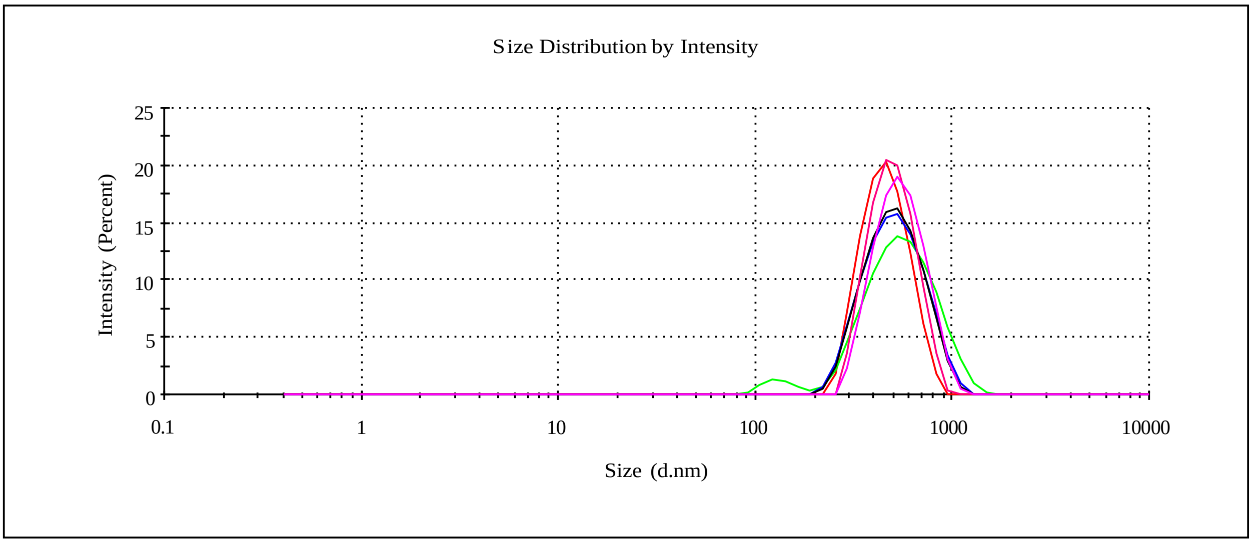

Figure S96. The size distribution (intensity) of systems **G1-cone**/noradrenaline (ratio 1:1, concentration of compounds 10^{-5} M) in phosphate buffer (pH = 7.4, 50 mM).

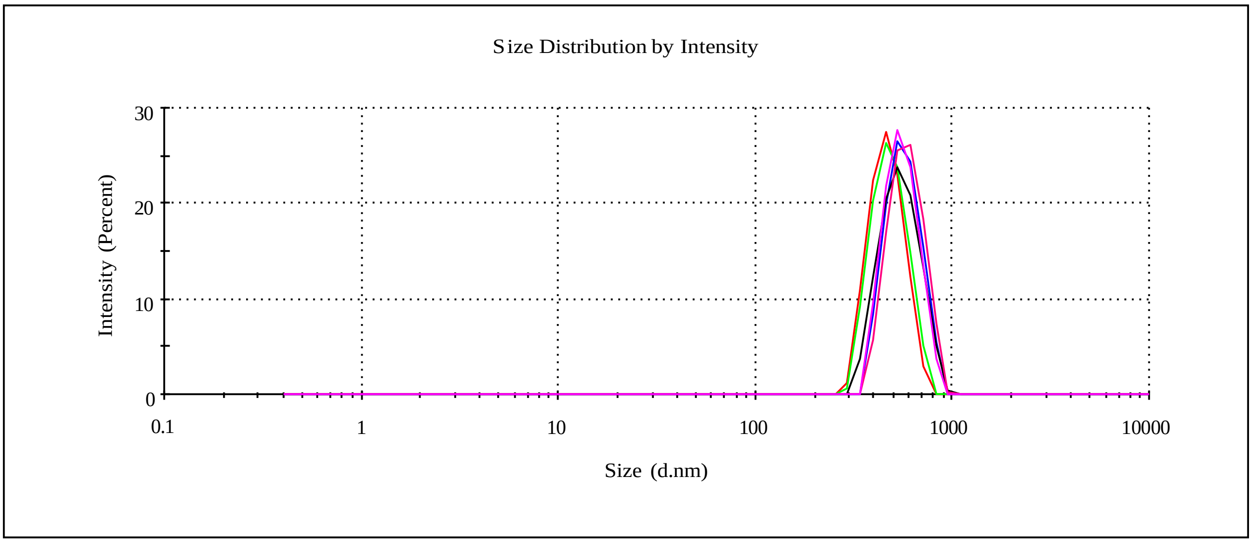

Figure S97. The size distribution (intensity) of systems **G1-paco**/noradrenaline (ratio 1:1, concentration of compounds 10^{-5} M) in phosphate buffer (pH = 7.4, 50 mM).

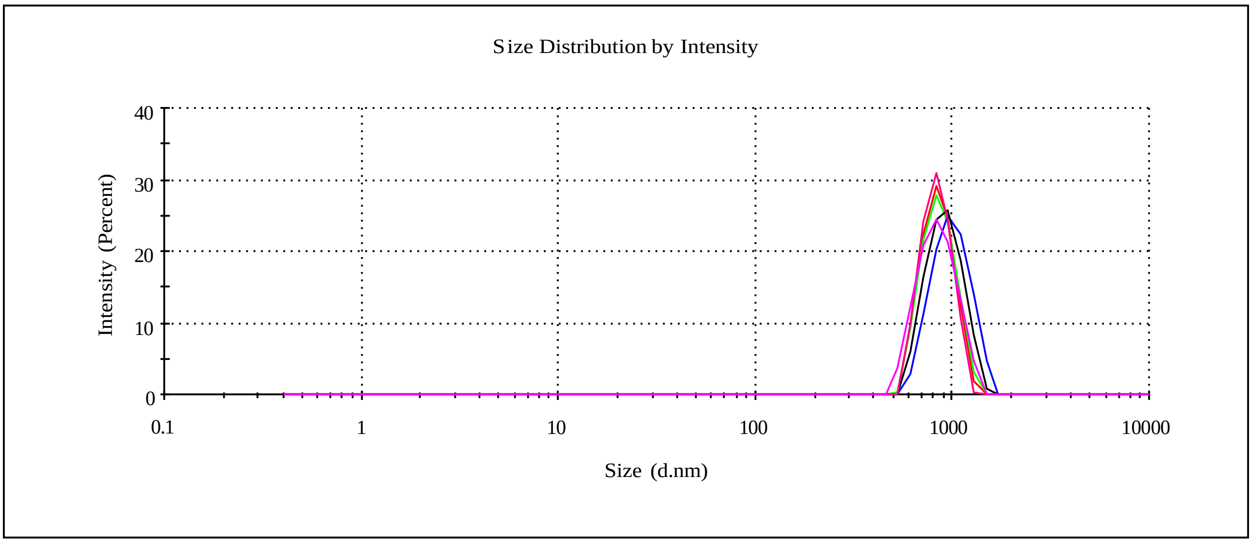

Figure S98. The size distribution (intensity) of systems **G1-alt**/noradrenaline (ratio 1:1, concentration of compounds 10^{-5} M) in phosphate buffer (pH = 7.4, 50 mM).

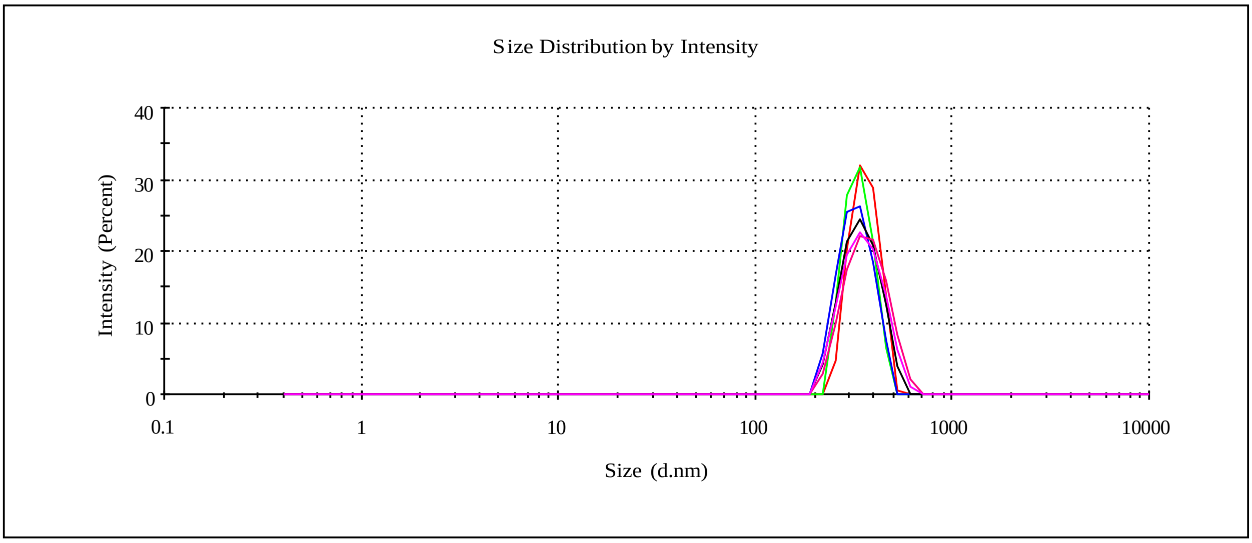

Figure S99. The size distribution (intensity) of systems **G2-cone**/noradrenaline (ratio 1:1, concentration of compounds 10^{-5} M) in phosphate buffer (pH = 7.4, 50 mM).

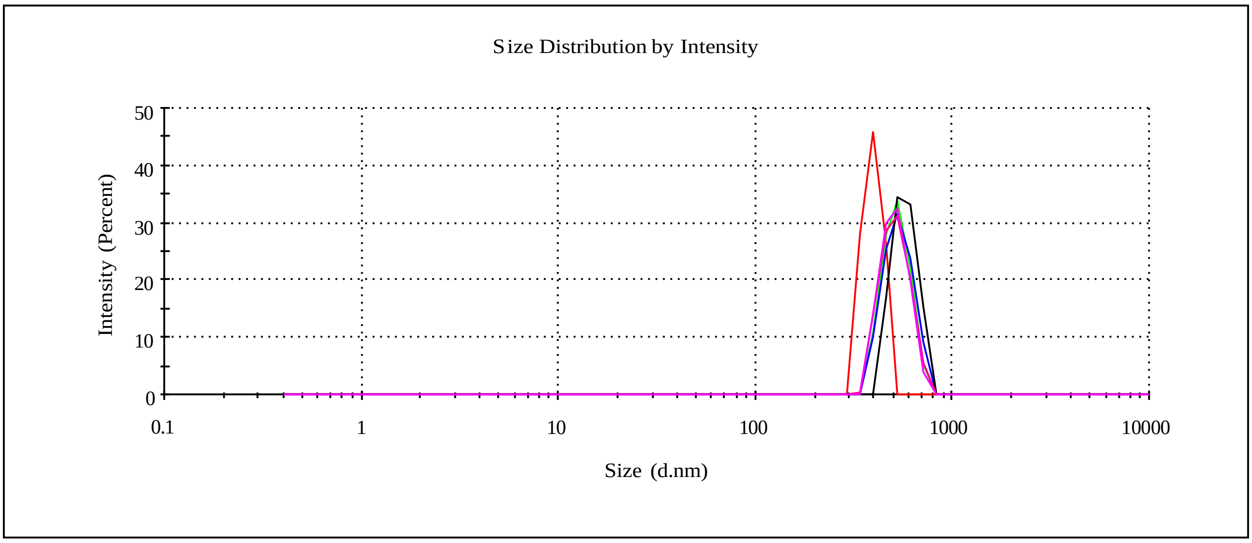

Figure S100. The size distribution (intensity) of systems **G2-paco**/noradrenaline (ratio 1:1, concentration of compounds 10^{-5} M) in phosphate buffer (pH = 7.4, 50 mM).

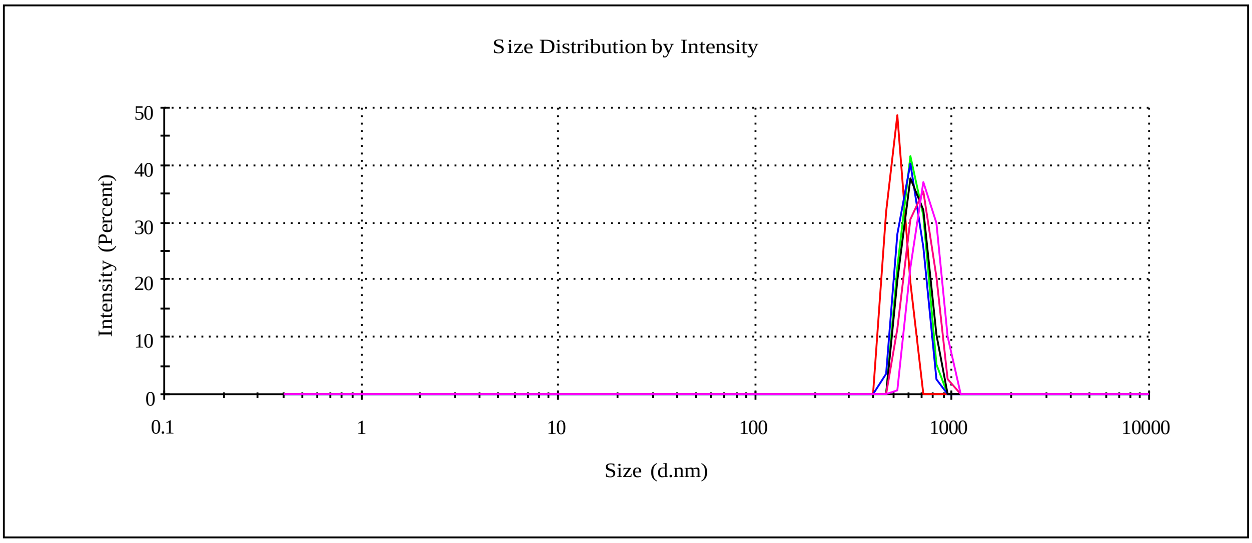

Figure S101. The size distribution (intensity) of systems **G2-alt**/noradrenaline (ratio 1:1, concentration of compounds 10^{-5} M) in phosphate buffer (pH = 7.4, 50 mM).

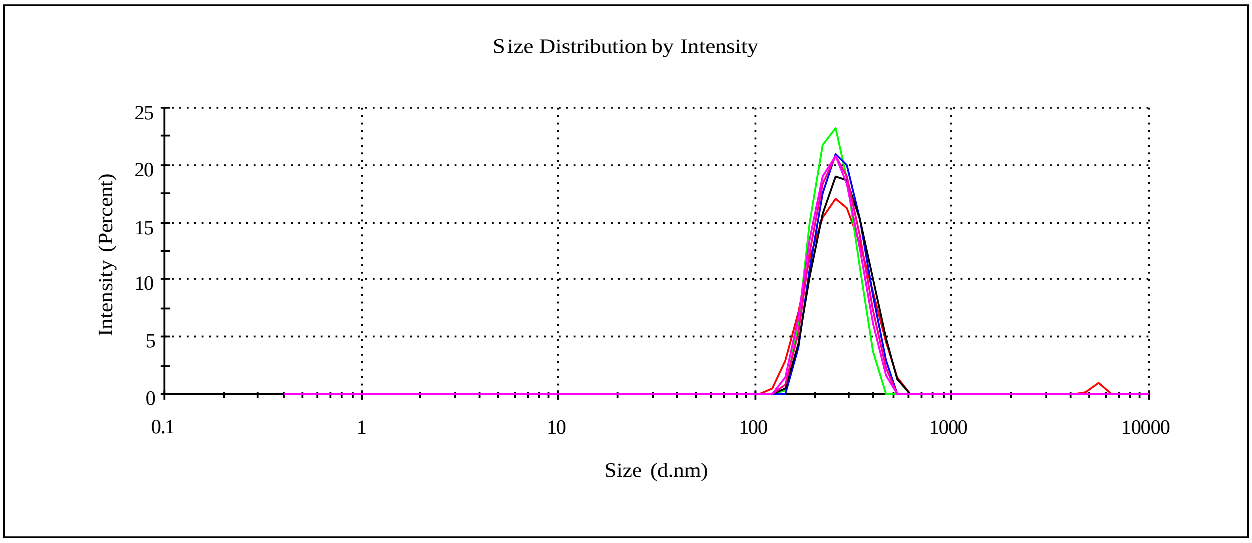

Figure S102. The size distribution (intensity) of systems **G1-cone**/adrenaline (ratio 1:1, concentration of compounds 10^{-5} M) in phosphate buffer (pH = 7.4, 50 mM).

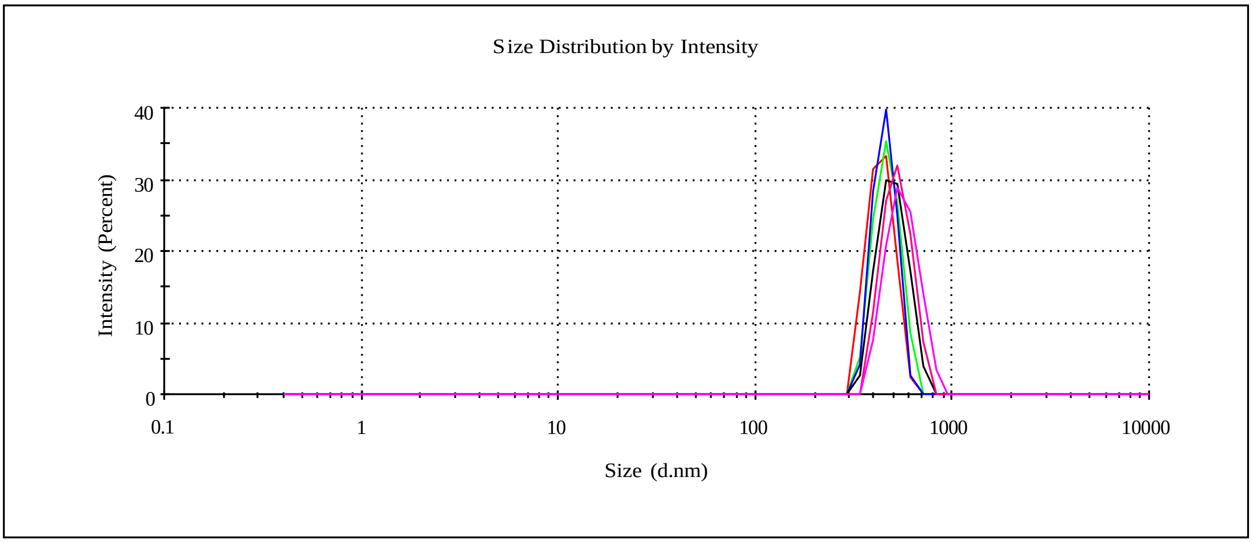

Figure S103. The size distribution (intensity) of systems **G1-paco**/adrenaline (ratio 1:1, concentration of compounds 10^{-5} M) in phosphate buffer (pH = 7.4, 50 mM).

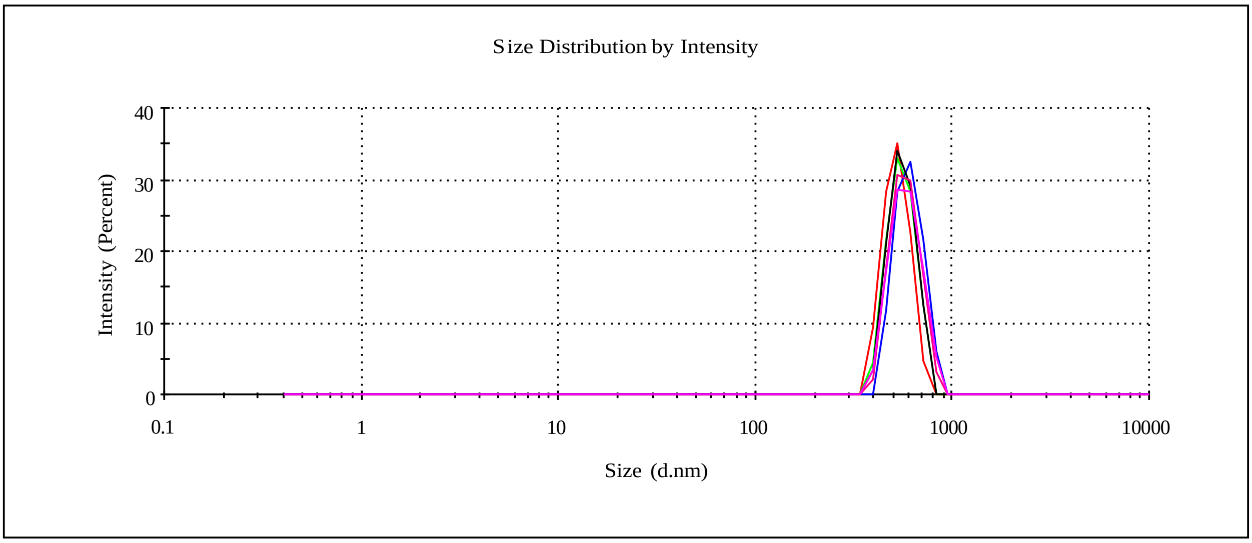

Figure S104. The size distribution (intensity) of systems **G1-alt**/adrenaline (ratio 1:1, concentration of compounds 10^{-5} M) in phosphate buffer (pH = 7.4, 50 mM).

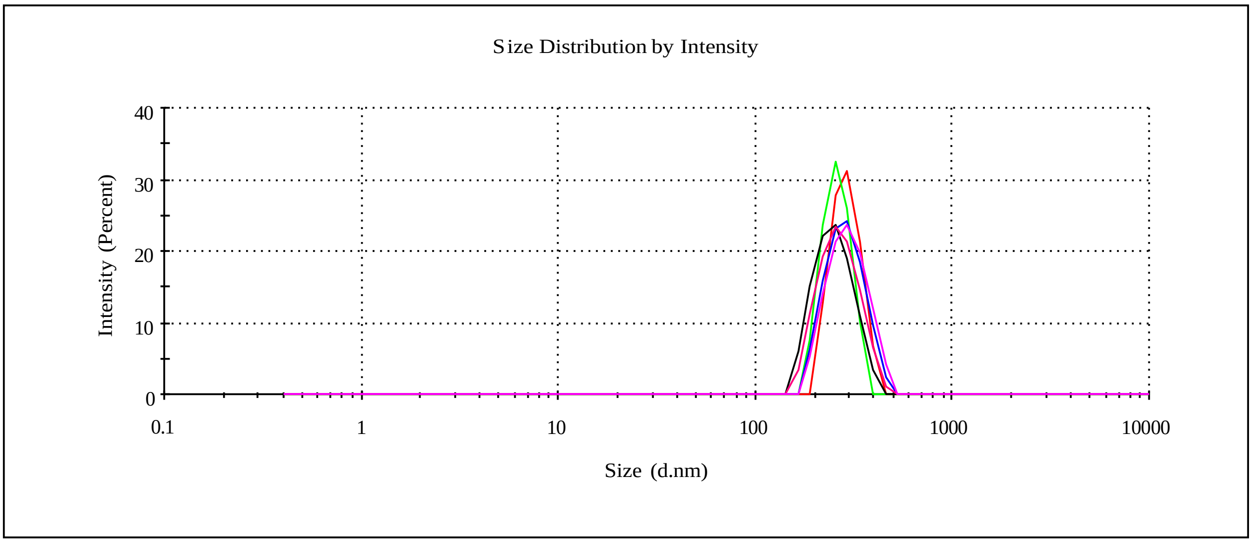

Figure S105. The size distribution (intensity) of systems **G2-cone**/adrenaline (ratio 1:1, concentration of compounds 10^{-5} M) in phosphate buffer (pH = 7.4, 50 mM).

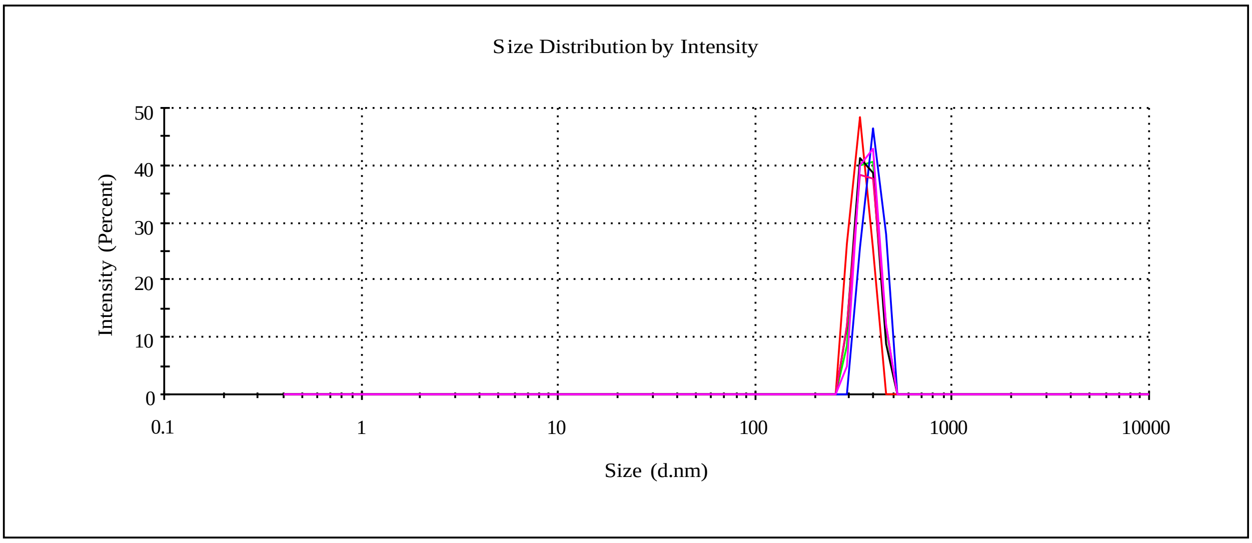

Figure S106. The size distribution (intensity) of systems **G2-paco**/adrenaline (ratio 1:1, concentration of compounds 10^{-5} M) in phosphate buffer (pH = 7.4, 50 mM).

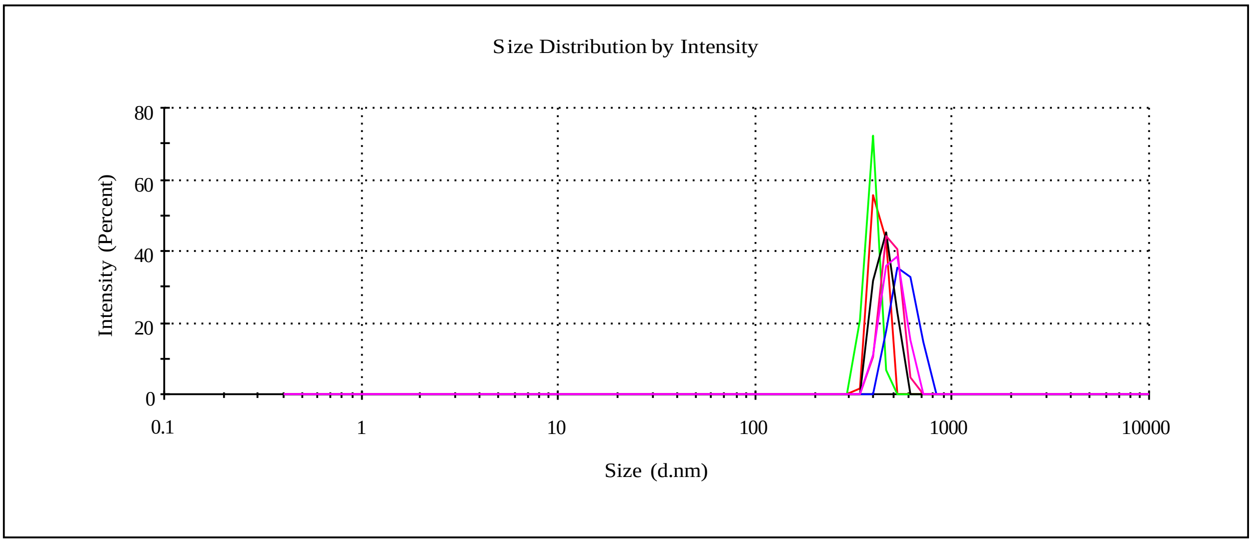

Figure S107. The size distribution (intensity) of systems **G2-alt**/adrenaline (ratio 1:1, concentration of compounds 10^{-5} M) in phosphate buffer (pH = 7.4, 50 mM).

3.5. TEM images

TEM analysis was carried with the Hitachi HT7700 Exalens microscope (Tokyo, Japan). The PAMAM-calix-dendrimers concentration was 10 µM, the concentration of catecholamines was 10 µM. The recording of the images of the mixture of catecholamines with PAMAM-calix-dendrimers was carried out in 1 h after mixing the solutions at 293 K.

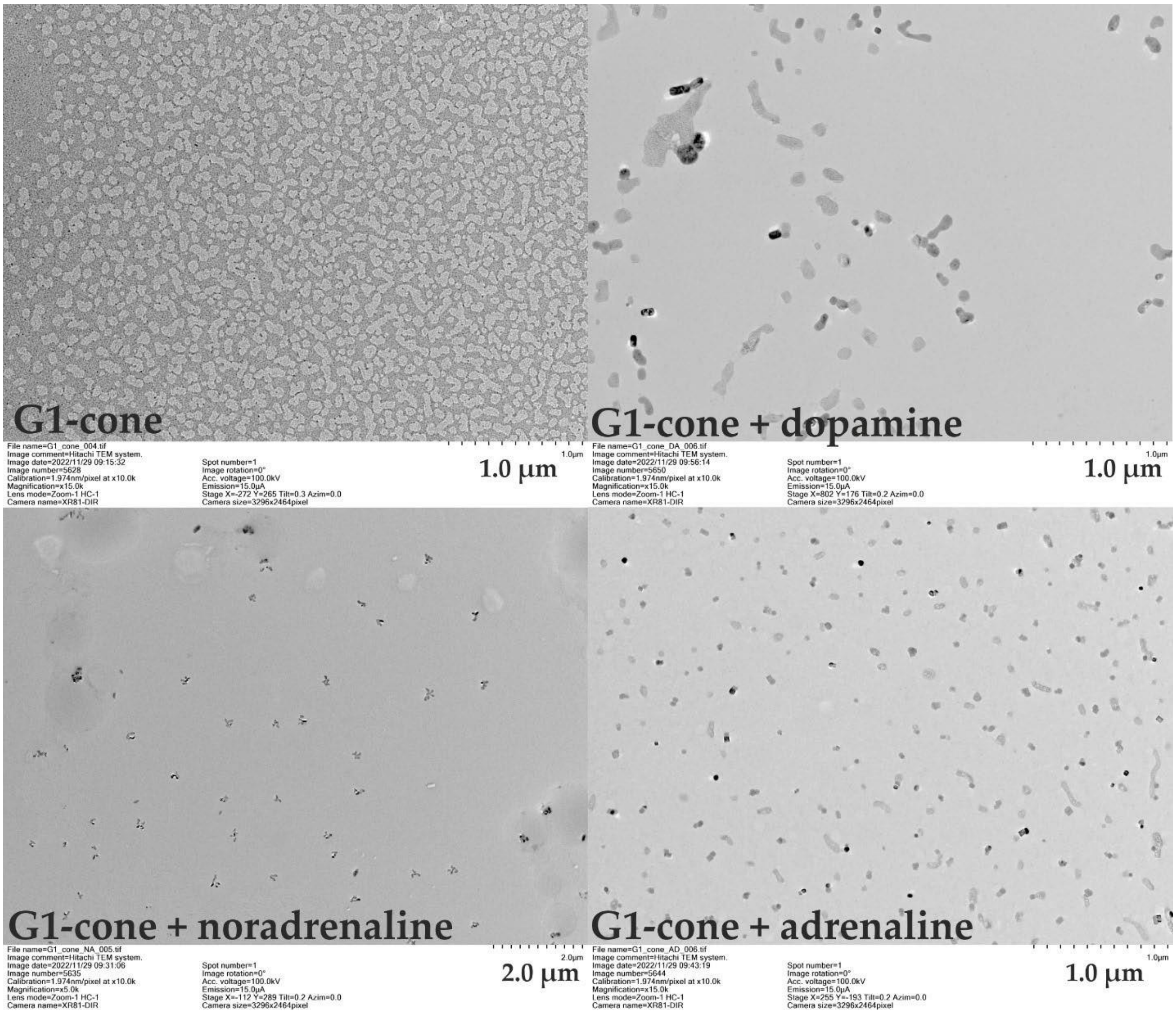

Figure S108. TEM images of pure **G1-cone** (10 µM) and associates of **G1-cone**/catecholamines (ratio 1:1, concentration of compounds 10 µM). Scale bar for **G1-cone**, **G1-cone**/dopamine and **G1-cone**/adrenaline: 1.0 µm; scale bar for **G1-cone**/noradrenaline 2.0 µm.

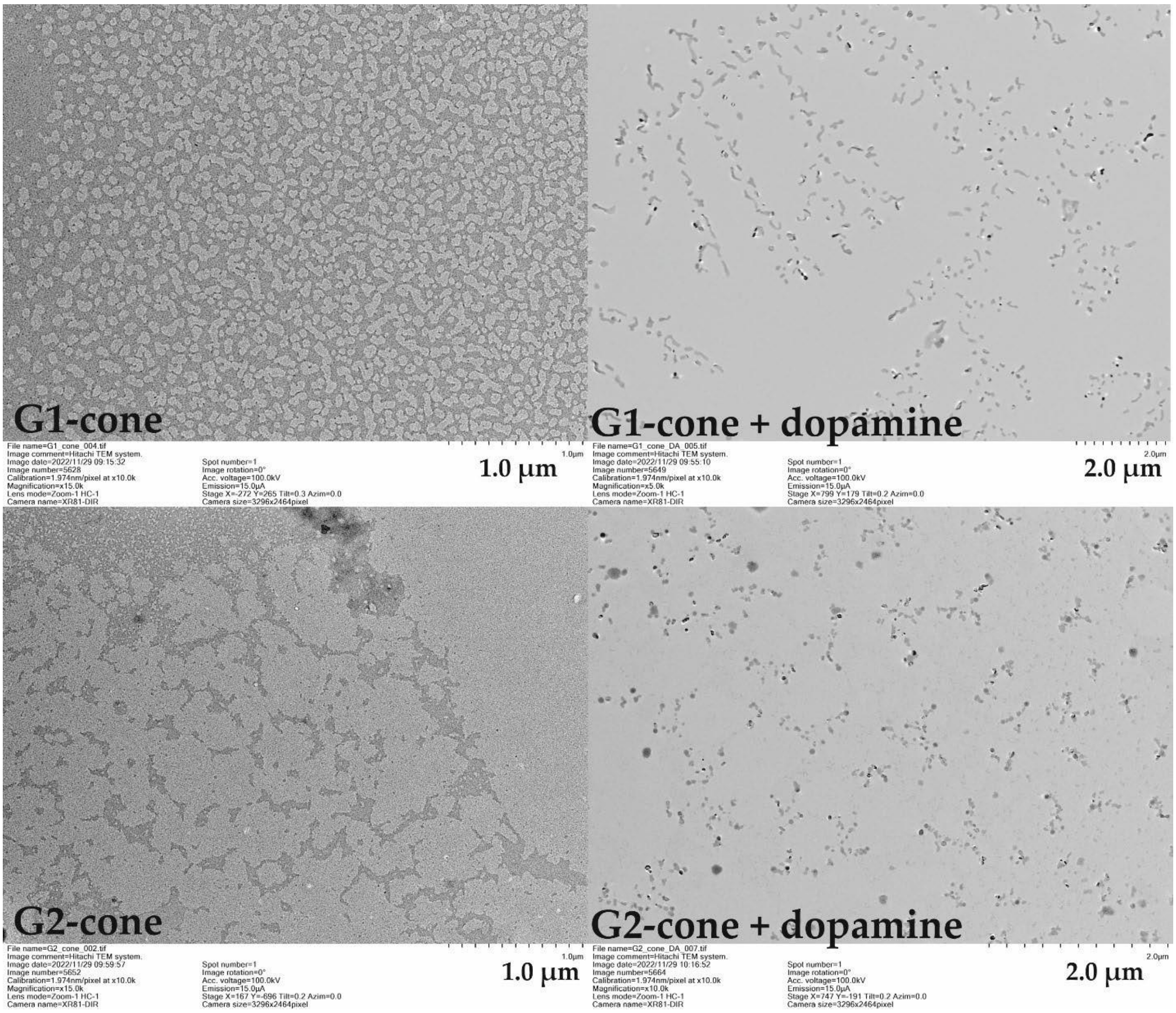

Figure S109. TEM images of pure **G1-cone** (10 µM), pure **G2-cone** (10 µM) and associates of **G1-cone**/dopamine (ratio 1:1, concentration of compounds 10 µM) and **G2-cone**/dopamine (ratio 1:1, concentration of compounds 10 µM). Scale bar for **G1-cone**, **G2-cone**: 1.0 µm; scale bar for **G1-cone**/dopamine and **G2-cone**/dopamine 2.0 µm.

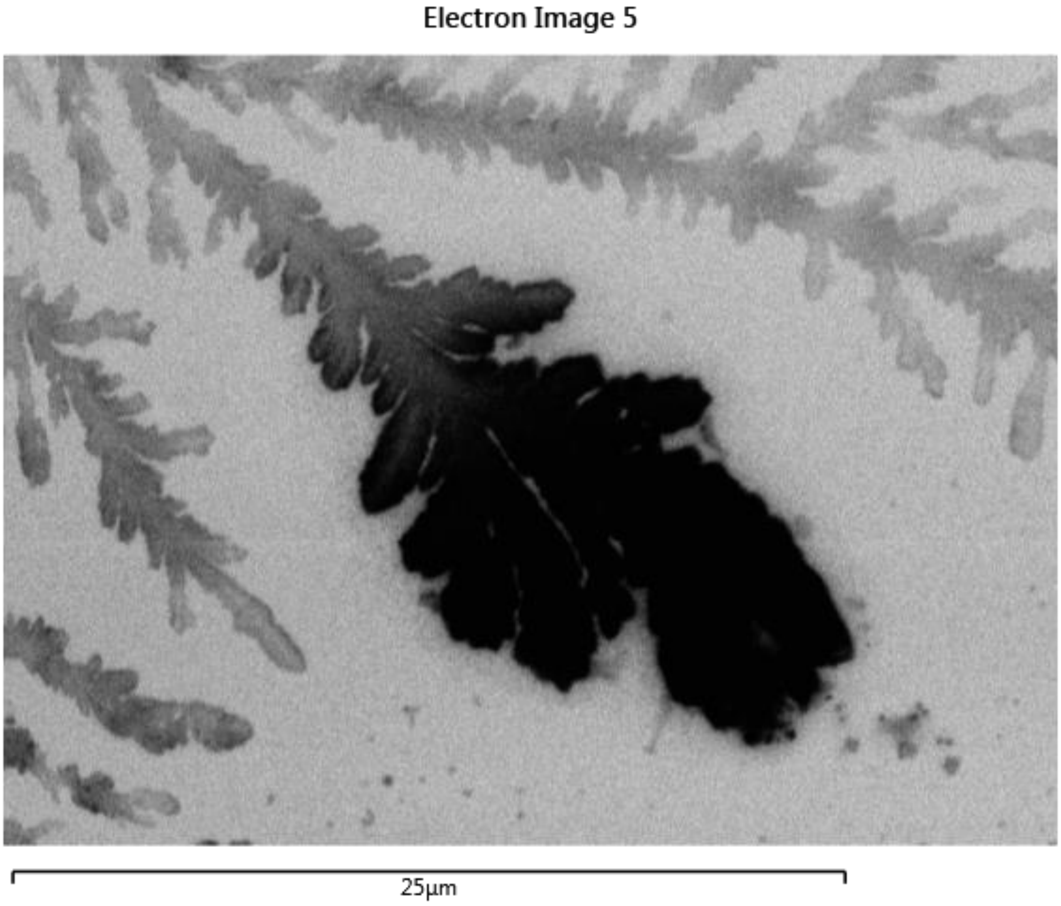

Figure S110. TEM image of associates of **G2-cone**/dopamine (ratio 1:1, concentration of compounds 10 µM). Scale bar 25 µm.

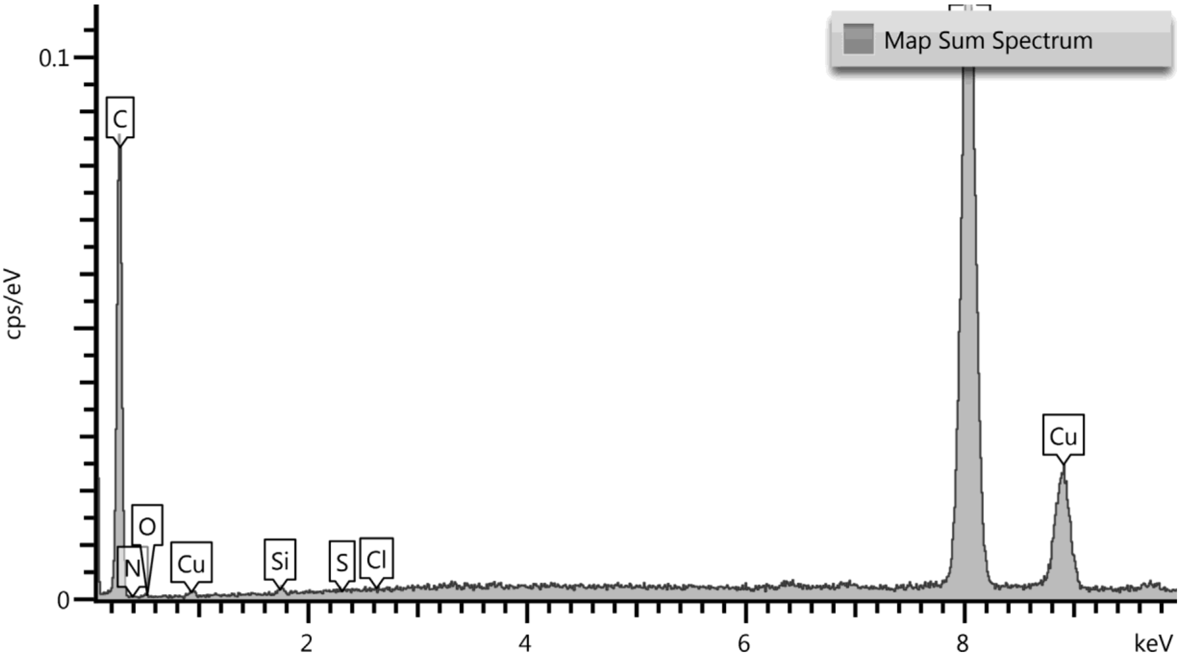

Figure S111. Energy-dispersion spectrum of associates of **G2-cone**/dopamine (ratio 1:1, concentration of compounds 10 µM).

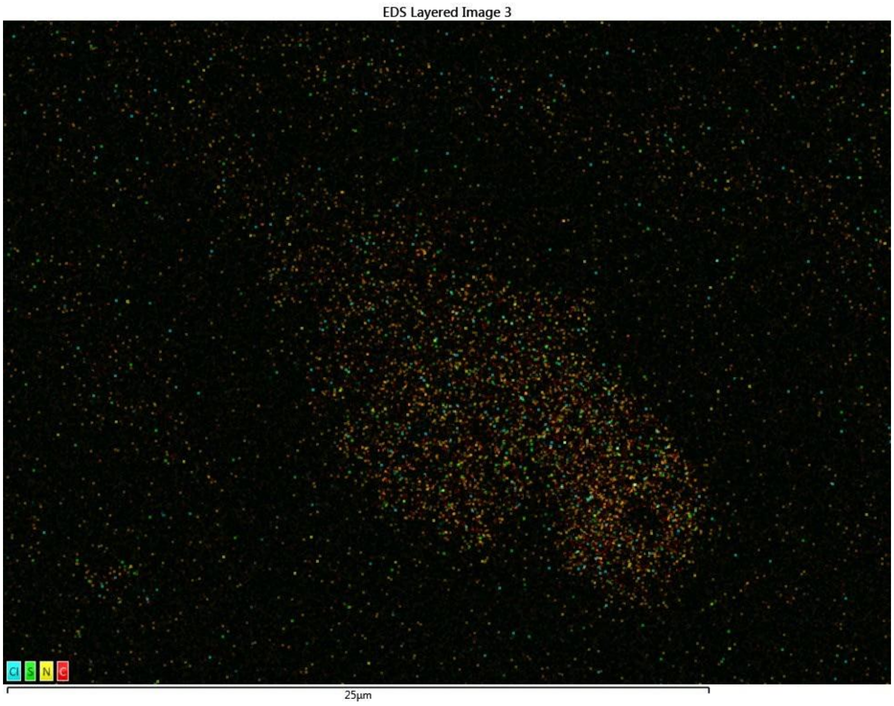

Figure S112. Superposition of elemental maps of C, N, S and Cl in the associates of **G2-cone**/dopamine (ratio 1:1, concentration of compounds 10 μM). Scale bar 25 μm.

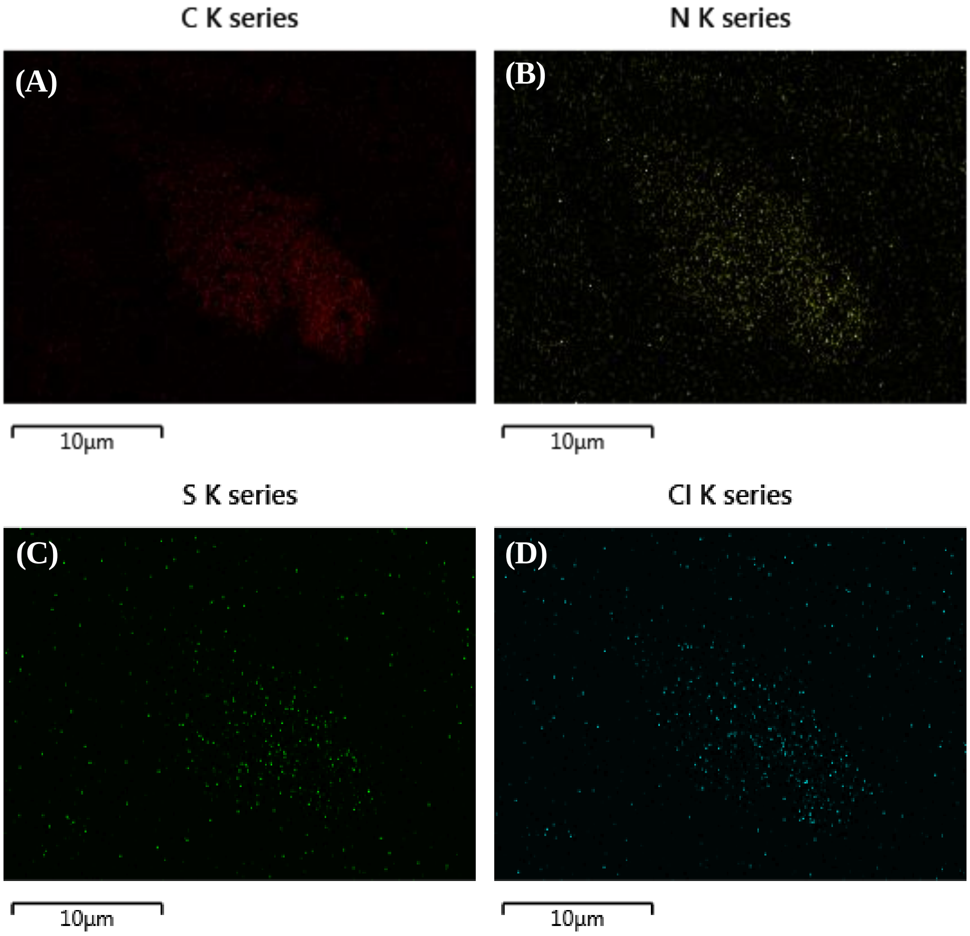

Figure S113. Mapping of C (A), N (B), S (C) and Cl (D) in the associates of **G2-cone**/dopamine (ratio 1:1, concentration of the compounds 10 μM). Scale bar 10 μm.

3.6. Release investigation

In vitro release investigation was carried out with dialysis membrane (Spectra/Por® 6 dialysis tubing, MWCO 1000). Concentration of **G2-cone** in mixtures was 0.5 mM, concentration of catecholamines (dopamine, noradrenaline and adrenaline) was 0.5 mM, ratio 1:1. Pure catecholamines were dissolved in 50 mM phosphate buffer (pH = 7.4) at the same concentration and used as control. 6 mL of these solutions were transferred to a dialysis tubings and then the dialysis tubings were placed in a 100 mL beakers containing 60 mL 50 mM phosphate buffer (pH = 7.4) at 25°C. After defined intervals, 3 mL of dialysate was withdrawn from each beaker for the fluorescence analysis and were returned to the systems afterwards.

The amount of catecholamine in the dialysate was investigated by fluorescence spectroscopy. Fluorescence spectra were recorded on the Fluorolog 3 luminescent spectrometer (Horiba Jobin Yvon, Longjumeau, France). The excitation wavelength was selected at 280 nm. The emission scan range was 300–540 nm. Excitation and emission slits were 2 nm. Quartz cuvettes with an optical path length of 10 mm were used. The cuvette was placed at the front face position to avoid inner filter effect. Fluorescence spectra were automatically corrected by the Fluorescence software. Spectra were recorded at 293 K. The catecholamines emission maxima for the release profiles were recorded at the 320 nm. The catecholamine release efficiency was calculated as follows: release efficiency = (emission maxima intensity of mixture dialysate/emission maxima intensity of control dialysate) × 100%.

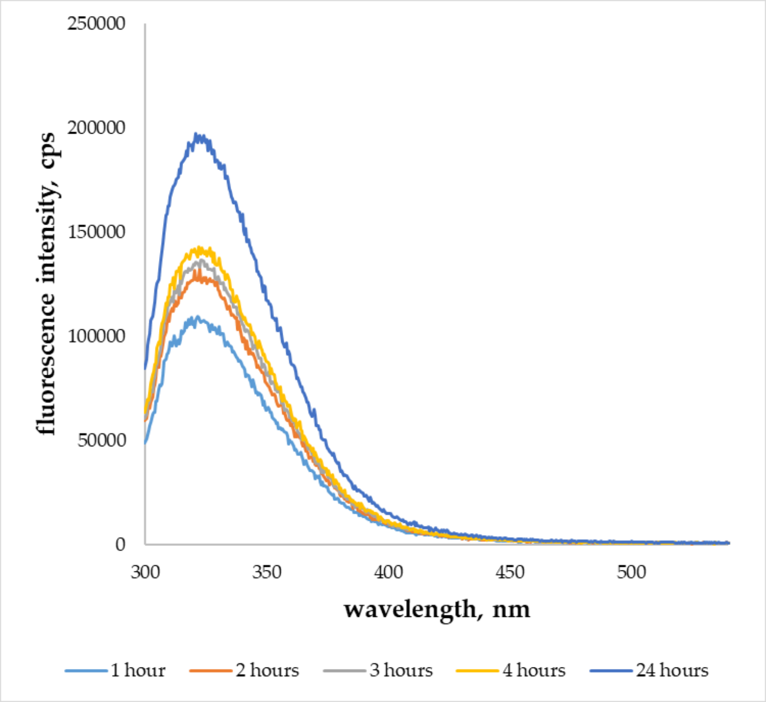

Figure S117. Fluorescence spectra of dialysate of pure dopamine over 24 h in phosphate buffer, pH = 7.4, 50 mM.

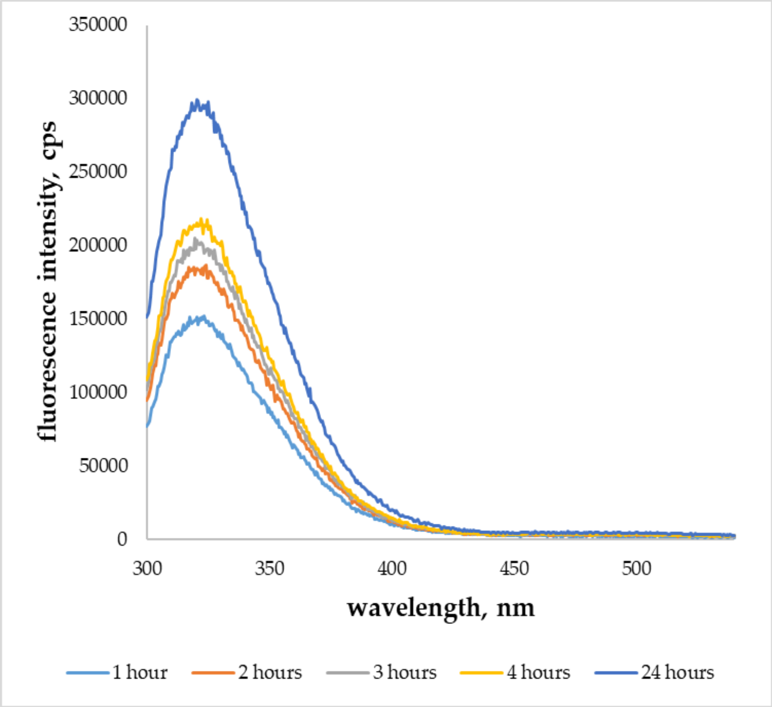

Figure S118. Fluorescence spectra of dialysate of pure noradrenaline over 24 h in phosphate buffer, pH = 7.4, 50 mM.

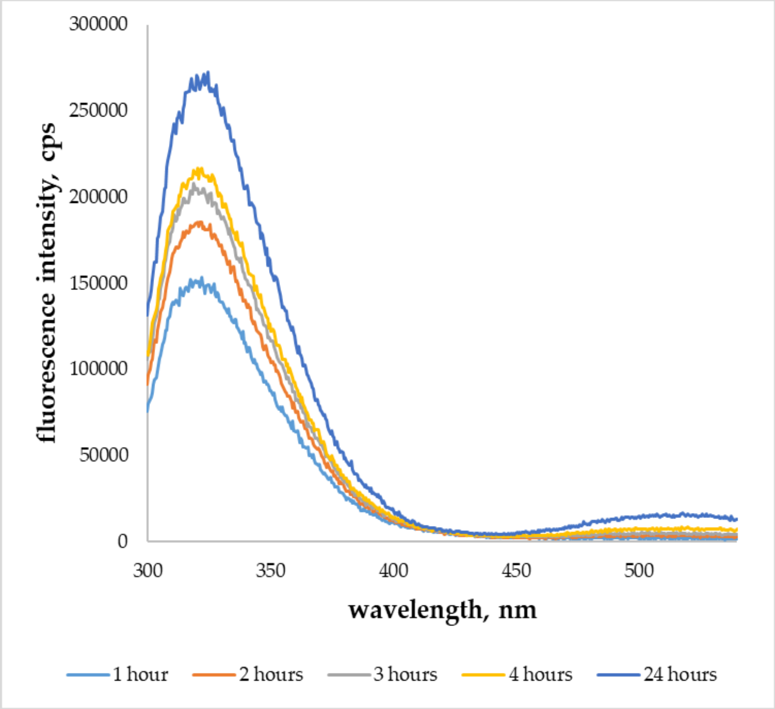

Figure S119. Fluorescence spectra of dialysate of pure adrenaline over 24 h in phosphate buffer, pH = 7.4, 50 mM.

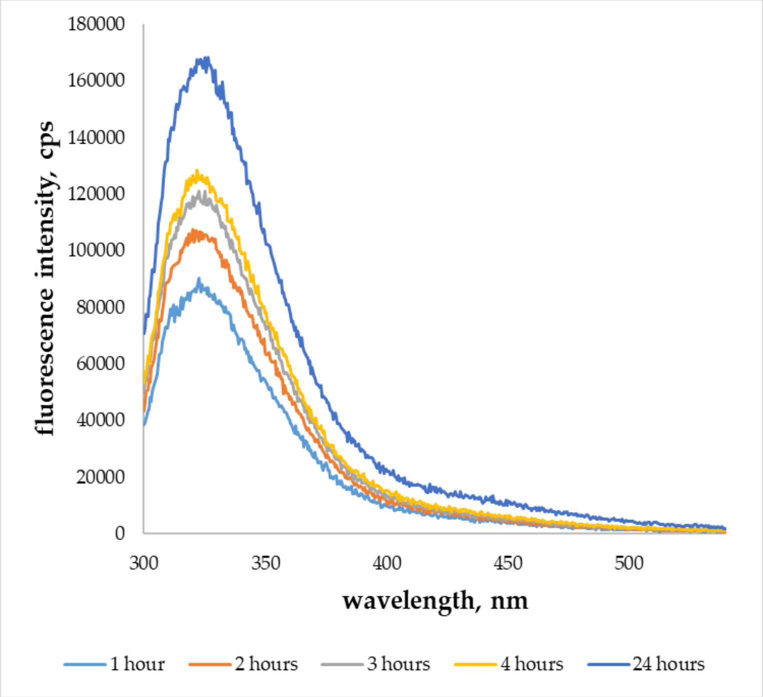

Figure S120. Fluorescence spectra of dialysate of **G2-cone**/dopamine mixture over 24 h in phosphate buffer, pH = 7.4, 50 mM.

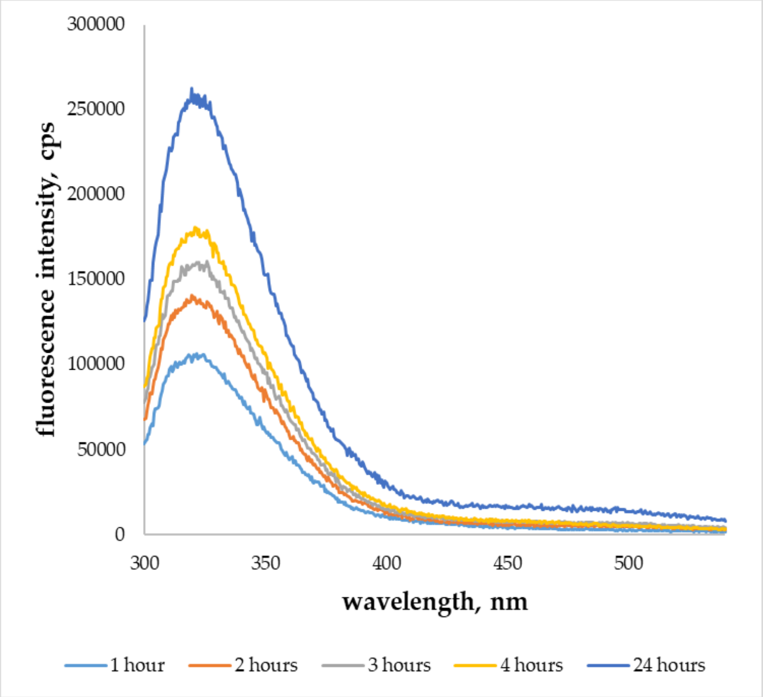

Figure S121. Fluorescence spectra of dialysate of **G2-cone**/noradrenaline mixture over 24 h in phosphate buffer, pH = 7.4, 50 mM.

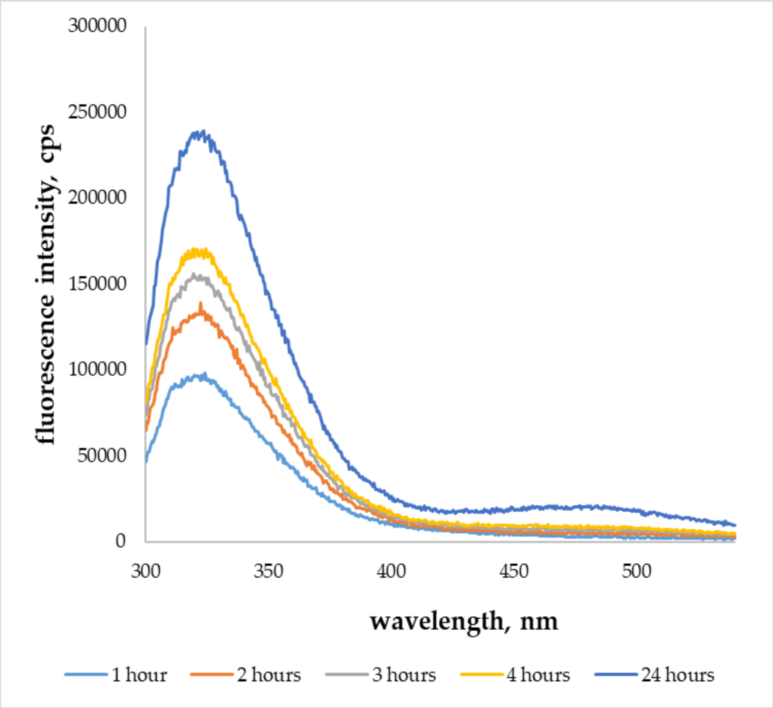

Figure S122. Fluorescence spectra of dialysate of **G2-cone**/adrenaline mixture over 24 h in phosphate buffer, pH = 7.4, 50 mM.

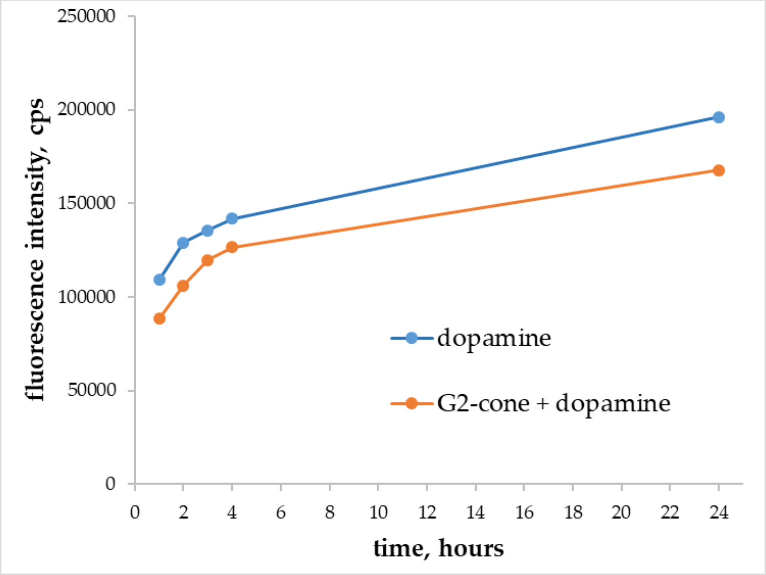

Figure S123. Emission maxima intensity of dialysate of pure dopamine solution and from **G2-cone**/dopamine over 24 h in phosphate buffer, pH = 7.4, 50 mM.

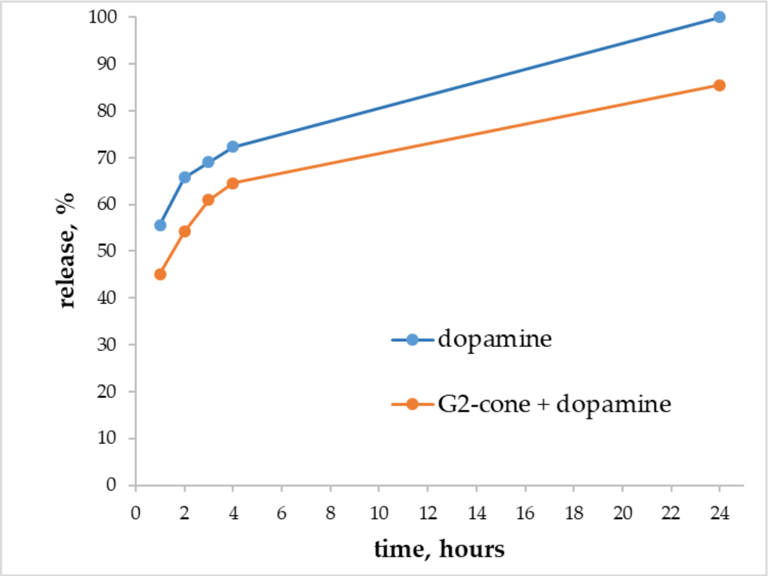

Figure S124. In vitro release profile of dopamine from pure dopamine solution and from **G2-cone**/dopamine mixture.

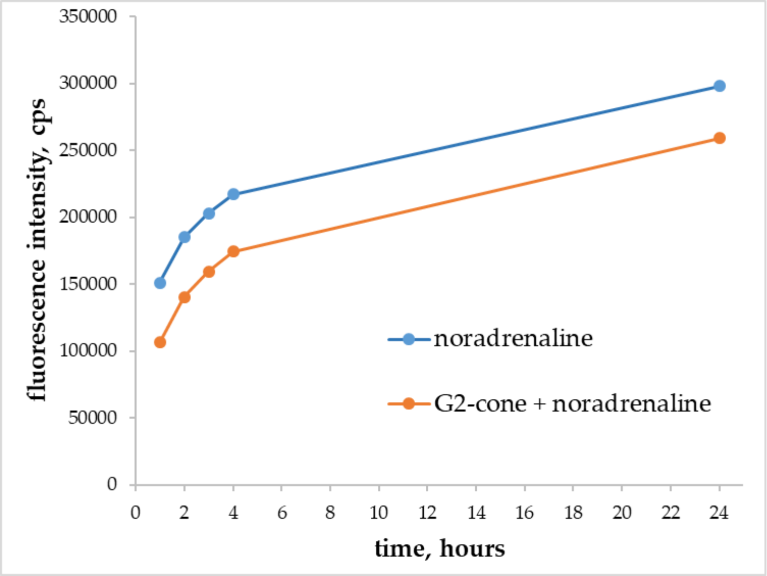

Figure S125. Emission maxima intensity of dialysate of pure noradrenaline solution and from **G2-cone**/noradrenaline over 24 h in phosphate buffer, pH = 7.4, 50 mM.

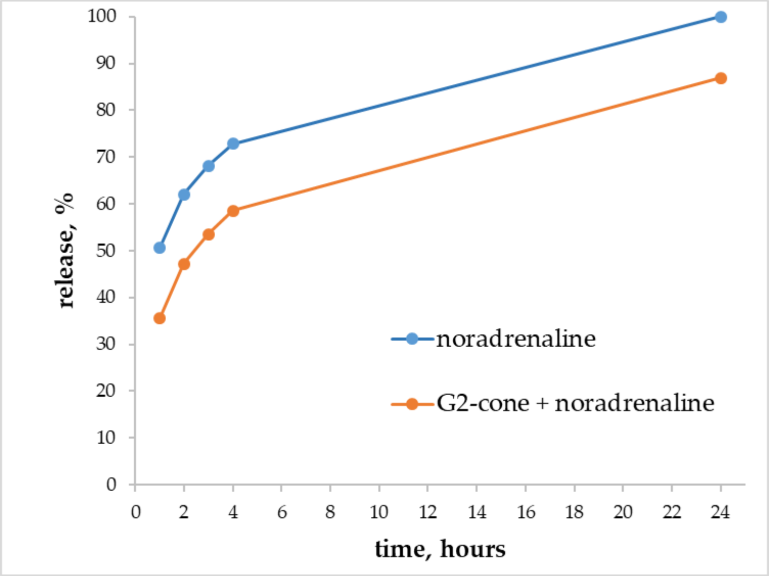

Figure S126. In vitro release profile of noradrenaline from pure noradrenaline solution and from **G2-cone**/noradrenaline mixture.

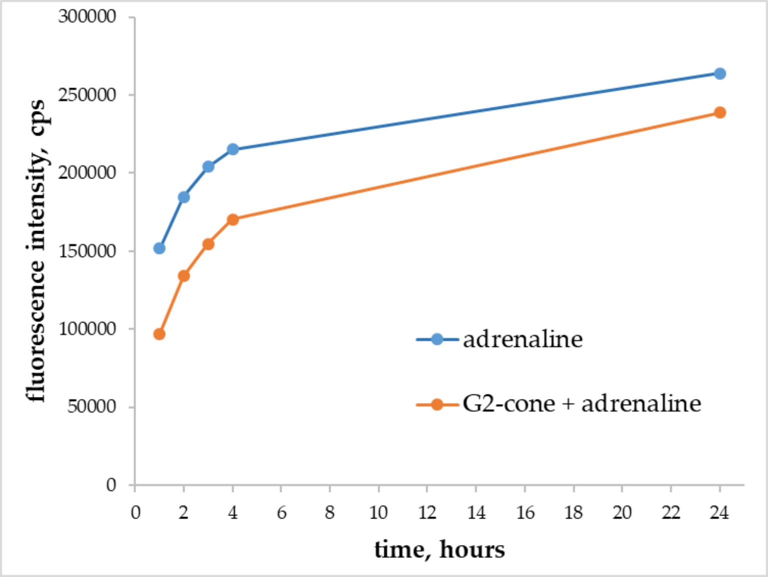

Figure S127. Emission maxima intensity of dialysate of pure adrenaline solution and from **G2-cone**/adrenaline over 24 h in phosphate buffer, pH = 7.4, 50 mM.

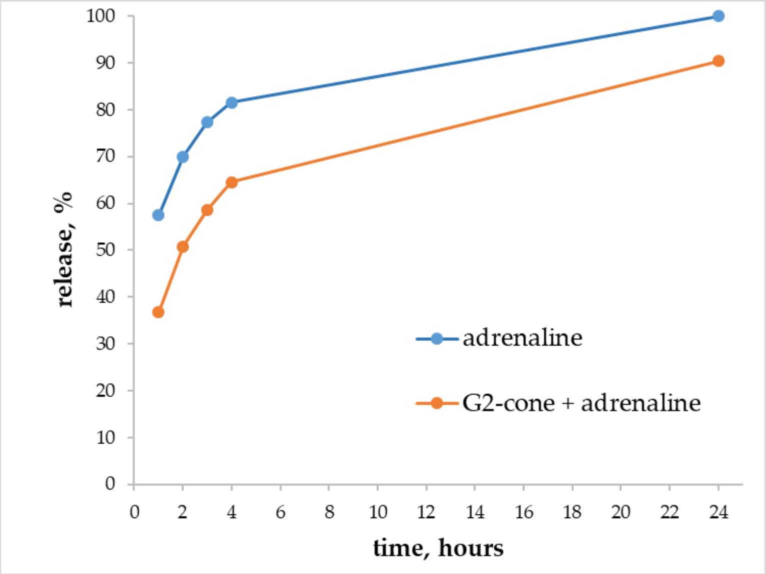

Figure S128. In vitro release profile of adrenaline from pure adrenaline solution and from G2-cone/adrenaline mixture.

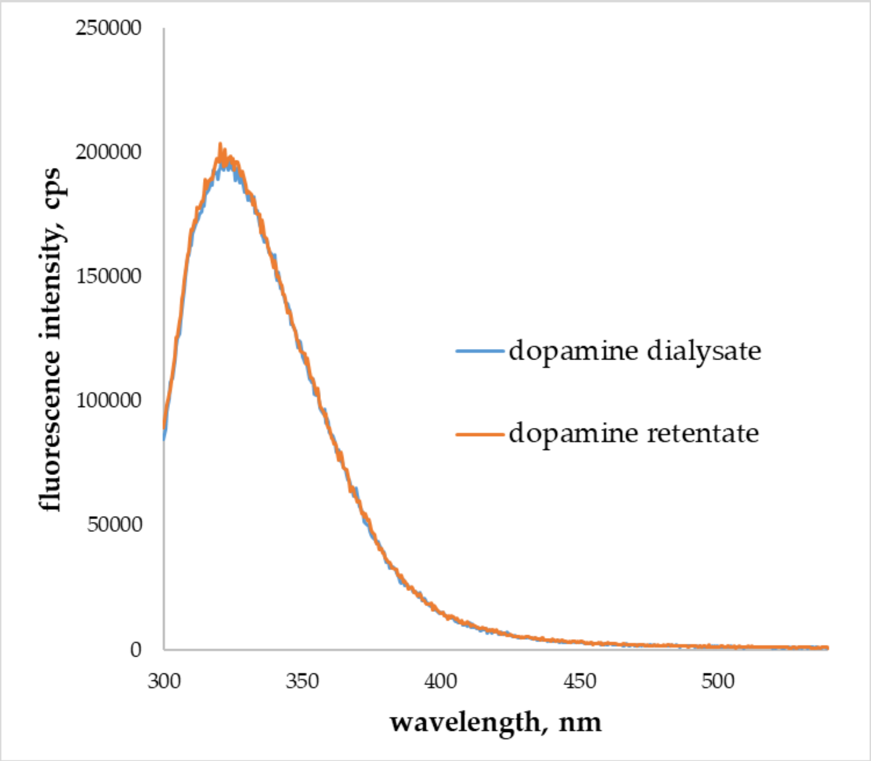

Figure S129. Fluorescence spectra of dialysate and retentate of pure dopamine after 24 h in phosphate buffer, pH = 7.4, 50 mM.

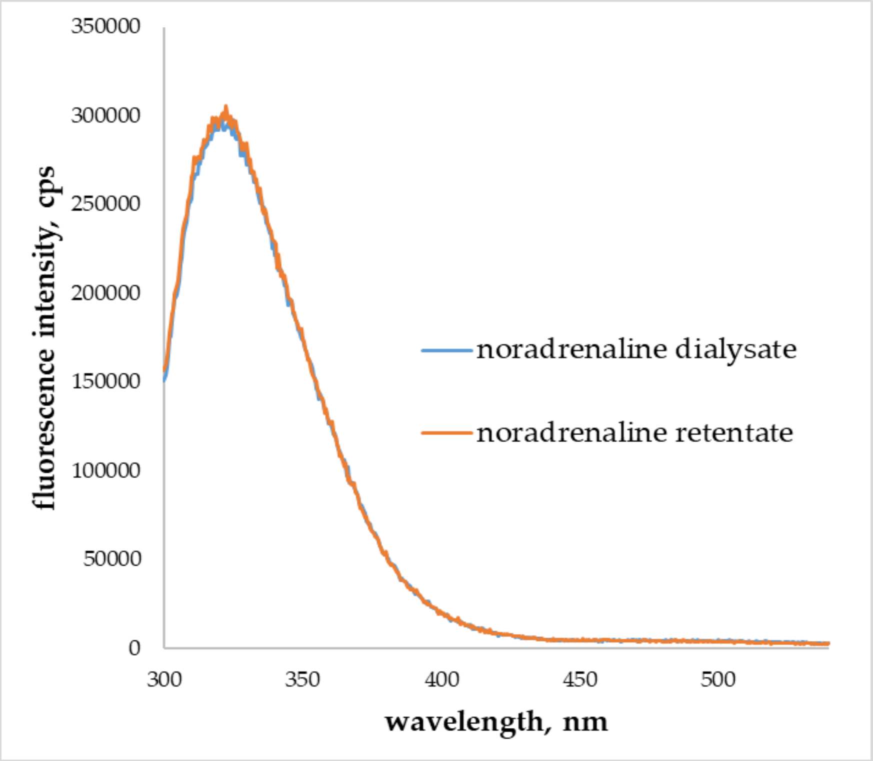

Figure S130. Fluorescence spectra of dialysate and retentate of pure noradrenaline after 24 h in phosphate buffer, pH = 7.4, 50 mM.

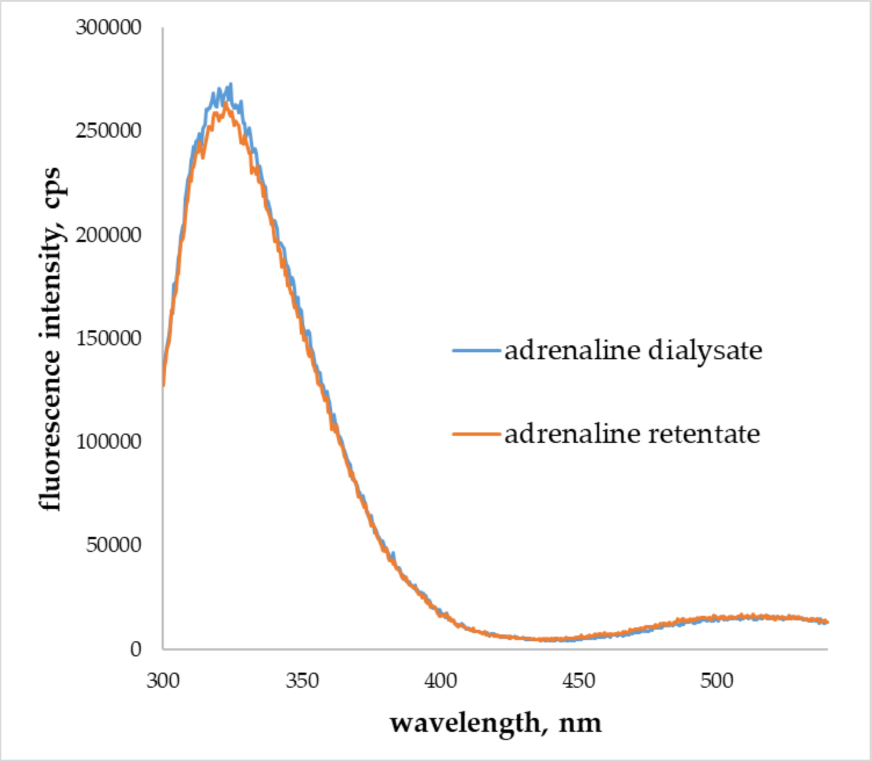

Figure S131. Fluorescence spectra of dialysate and retentate of pure adrenaline after 24 h in phosphate buffer, pH = 7.4, 50 mM.

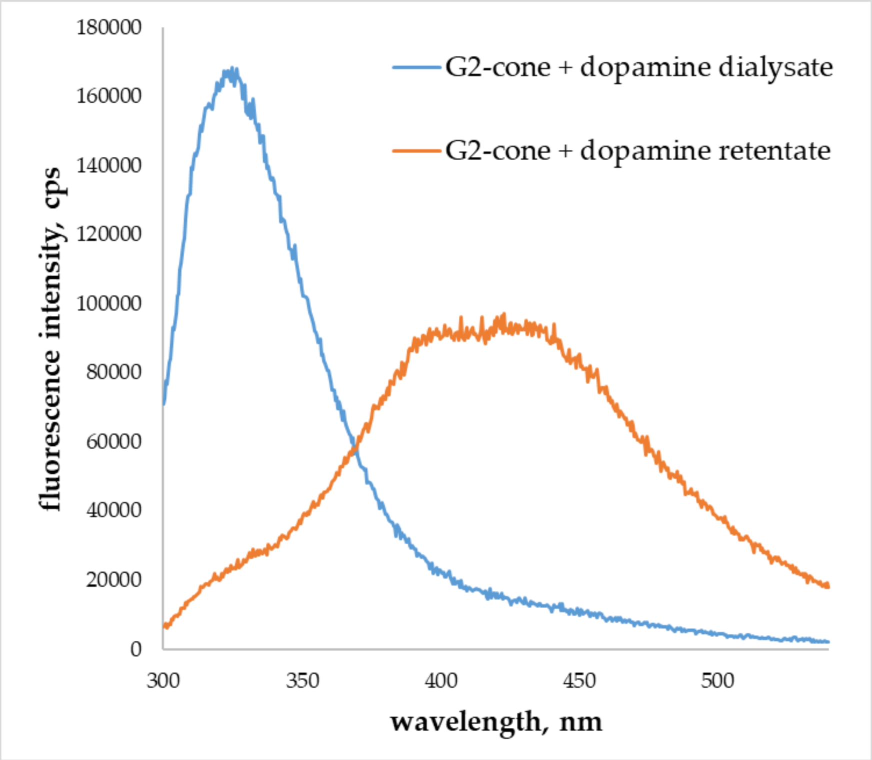

Figure S132. Fluorescence spectra of dialysate and retentate of **G2-cone**/dopamine mixture after 24 h in phosphate buffer, pH = 7.4, 50 mM.

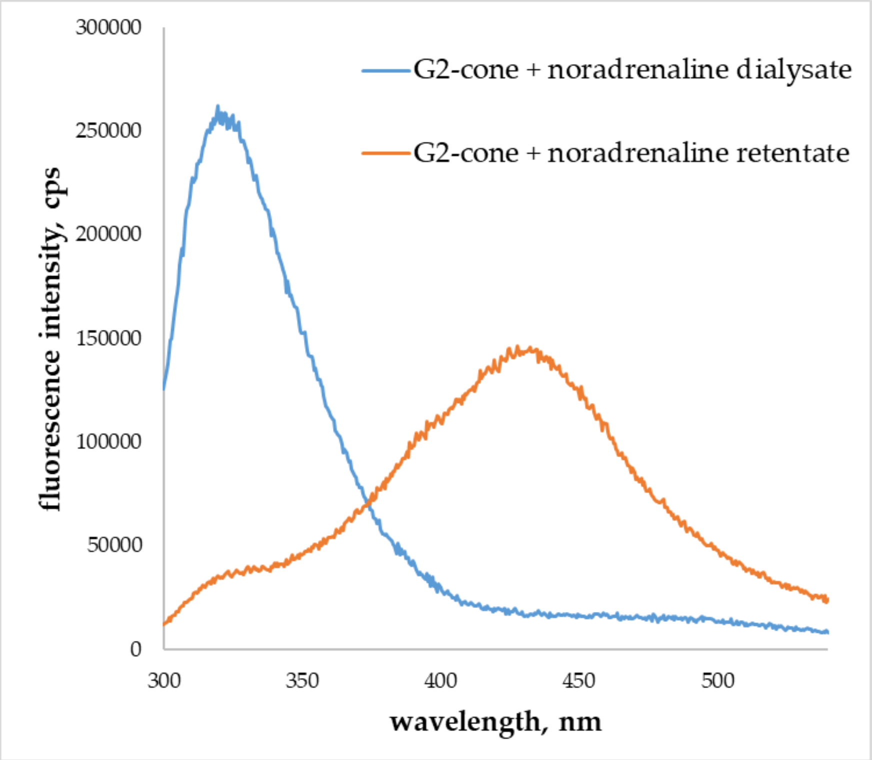

Figure S133. Fluorescence spectra of dialysate and retentate of **G2-cone**/noradrenaline mixture after 24 h in phosphate buffer, pH = 7.4, 50 mM.

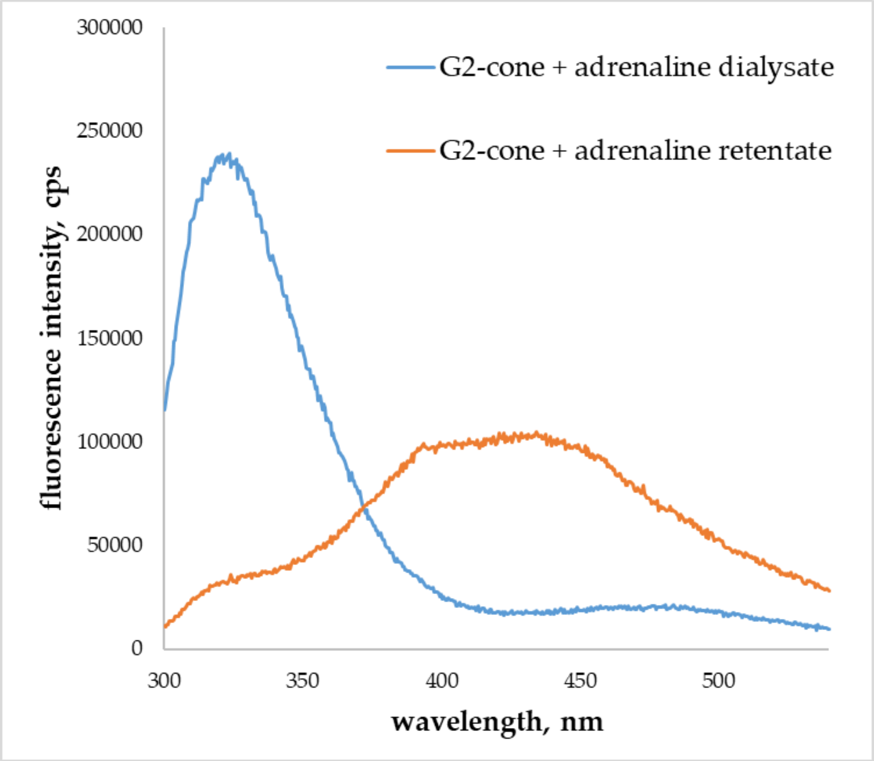

Figure S134. Fluorescence spectra of dialysate and retentate of **G2-cone**/adrenaline mixture after 24 h in phosphate buffer, pH = 7.4, 50 mM.
